# *DEBATE-*statistical analysis plans for observational studies

**DOI:** 10.1186/s12874-019-0879-5

**Published:** 2019-12-09

**Authors:** Bart Hiemstra, Frederik Keus, Jørn Wetterslev, Christian Gluud, Iwan C. C. van der Horst

**Affiliations:** 1 Department of Anesthesiology, University of Groningen, University Medical Center Groningen, Hanzeplein 1, PO Box 30 001, 9700 RB Groningen, The Netherlands; 2 Department of Critical Care, University of Groningen, University Medical Center Groningen, Groningen, The Netherlands; 3 The Copenhagen Trial Unit (CTU), Centre for Clinical Intervention Research, Rigshospitalet, Copenhagen University Hospital, Copenhagen, Denmark; 4 Department of Intensive Care, University of Maastricht, Maastricht University Medical Center+, Maastricht, the Netherlands

## Abstract

**Background:**

All clinical research benefits from transparency and validity. Transparency and validity of studies may increase by prospective registration of protocols and by publication of statistical analysis plans (SAPs) before data have been accessed to discern data-driven analyses from pre-planned analyses.

**Main message:**

Like clinical trials, recommendations for SAPs for observational studies increase the transparency and validity of findings. We appraised the applicability of recently developed guidelines for the content of SAPs for clinical trials to SAPs for observational studies. Of the 32 items recommended for a SAP for a clinical trial, 30 items (94%) were identically applicable to a SAP for our observational study. Power estimations and adjustments for multiplicity are equally important in observational studies and clinical trials as both types of studies usually address multiple hypotheses. Only two clinical trial items (6%) regarding issues of randomisation and definition of adherence to the intervention did not seem applicable to observational studies. We suggest to include one new item specifically applicable to observational studies to be addressed in a SAP, describing how adjustment for possible confounders will be handled in the analyses.

**Conclusion:**

With only few amendments, the guidelines for SAP of a clinical trial can be applied to a SAP for an observational study. We suggest SAPs should be equally required for observational studies and clinical trials to increase their transparency and validity.

## Background

Transparency is considered fundamental for the reproducibility of any research finding [1]. Initiatives such as SPIRIT, CONSORT, PRISMA, and PROSPERO have contributed to transparent reporting of protocols and findings of randomised clinical trials and systematic reviews [2–5]. Still, the multitude of decisions taken during the statistical analysis phase of any study have been shown to impact on results and conclusions, irrespective of pre-published protocols [6]. While any protocol for a clinical study should include the principle features of the statistical analysis of the data, a statistical analysis plan (SAP) should fully outline the details of all planned analyses, including any additional analyses. Recently, Gamble and colleagues used a Delphi survey to reach consensus and provide recommendations for a minimum set of items that should be addressed in a SAP for a randomised clinical trial [7].

Observational studies are frequently the source for multiple statistical analyses and reports. Guidelines for reporting such as STROBE, TRIPOD or STARD are key to transparent reporting of findings of observational studies [8–10], but these do not reduce the number of possible decisions taken during the analysis phase of such studies. Like randomised clinical trials, the validity of conclusions of cohort studies is likely to improve by use of published SAPs to distinguish pre-planned analyses from data-driven exercises [1, 11]. Journals now encourage researchers to preregister observational studies and SAPs [11–15], but there are no guidelines on the required content of the latter.

Therefore, we argue that SAP guidelines should also be developed for observational studies. In the absence of such a guideline, we appraised and modified recently developed recommendations for the content of SAPs for clinical trials to be used for observational studies. This paper reports the applicability of SAP guidelines for clinical trials to our single-centre observational study, of which the study design is described elsewhere [16].

## Main text: recommended content of SAPs in observational studies

We have appraised the recommendations for the content of SAPs for clinical trials and assessed the applicability of each section to be used for an observational study (Table 1). We added the item ‘confounding’ to the recommended list for observational studies. Compared to clinical trials, confounding is an even more pronounced issue in observational studies and should be considered during model building.

**Table 1:** Applicability of recommend content of statistical analysis plans for clinical trials to observational studies

Section/Item	Index	Description for clinical trials	Description for observational studies
Section 1: Administrative information			
Title and *study* registration	1a	Descriptive title that matches the protocol, with SAP either as a forerunner or subtitle, and trial acronym	Descriptive title that matches the protocol, with SAP either as a forerunner or subtitle, and *study* acronym
	1b	Trial registration number	*Study* registration number
SAP version	2	SAP version number with dates	Unchanged
Protocol version	3	Reference to version of protocol being used	Unchanged
SAP revisions	4a	SAP revision history	Unchanged
	4b	Justification for each SAP revision	Unchanged
	4c	Timing of SAP revisions in relation to interim analyses, etc.	Timing of SAP revisions in relation to *planned repetitive* analyses
Roles and responsibility	5	Names, affiliations, and roles of SAP contributors	Unchanged
Signatures of:	6a	Person writing the SAP	Unchanged
	6b	Senior statistician responsible	Unchanged
	6c	Chief investigator/clinical lead	Unchanged
Section 2: Introduction			
Background and rationale	7	Synopsis of trial background and rationale including a brief description of research question and brief justification for undertaking the trial	Synopsis of *study* background and rationale including a brief description of research question and brief justification for undertaking the *study*
Objectives	8	Description of specific objectives and hypotheses	Description of specific objectives and hypotheses, *including secondary objectives*
Section 3: Study methods			
*Study* design	9	Brief description of trial design including type of trial (e.g. parallel group, multi-arm, crossover, factorial and allocation ratio and may include brief description of interventions)	Brief description of *study* design including type of study *(*e.g. *case-control, cross-sectional or cohort study)*
*Randomization*	10	*Randomization details,* e.g.*, whether any minimization or stratification occurred (including stratifying factors used or the location of that information if it is not held within the SAP)*	Not applicable
*Power considerations*	11	Full sample size calculation or reference to sample size calculation in protocol (instead of replication in SAP)	In case of an unspecified sample size, provide power calculations for (at least) the primary analysis or present a detectable difference with a specified power*
Framework	12	Superiority, equivalence, or noninferiority hypothesis testing framework, including which comparisons will be presented on this basis	Unchanged*
Statistical *repetitive* analyses and stopping guidance	13a	Information on interim analyses specifying what interim analyses will be carried out and listing of time points	Information on *repetitive* analyses specifying what *repetitive* analyses will be carried out and listing of time points*
	13b	Any planned adjustment of the significance level due to interim analysis	Any planned adjustment of the significance level due to *repetitive analyses*
	13c	Details of guidelines for stopping the trial early	Details of guidelines for stopping the *study* early
Timing of final analysis	14	Timing of final analysis, e.g., all outcomes analysed collectively or timing stratified by planned length of follow-up	Unchanged*
Timing of outcome assessments	15	Time points at which the outcomes are measured including visit “windows”	Unchanged
Section 4: Statistical principles			
Confidence intervals and *P*-values	16	Level of statistical significance	Unchanged*
	17	Description and rationale for any adjustment for multiplicity and, if so, detailing how the type 1 error is to be controlled	Unchanged*
	18	Confidence interval to be reported	Unchanged
Adherence and protocol deviations	*19a*	*Definition of adherence to the intervention and how this is assessed including extent of exposure*	Not applicable
	*19b*	*Description of how adherence to the intervention will be presented*	Not applicable
	19c	Definition of protocol deviations for the trial	Definition of protocol deviations for the *study*
	19d	Description of which protocol deviations will be summarized	Unchanged
Analysis populations	20	Definition of analysis populations, e.g., intention to treat, per protocol, complete case, safety	Definition of analysis populations, e.g., per protocol, complete case, safety
Section 5: Study Population			
Screening data	21	Reporting of screening data (if collected) to describe representativeness of trial sample	Reporting of screening data (if collected) to describe representativeness of study sample
Eligibility	22	Summary of eligibility criteria	Unchanged
Recruitment	23	Information to be included in the CONSORT flow diagram	Information to be included in the *STROBE* flow diagram
Withdrawal/follow-up	24a	Level of withdrawal, e.g., from intervention and/or from follow-up	Level of withdrawal, e.g., *dropouts after inclusion or refusal to be contacted for additional information*
	24b	Timing of withdrawal/lost to follow-up data	Unchanged
	24c	Reasons and details of how withdrawal/lost to follow-up data will be presented	Unchanged
Baseline patient characteristics	25a	List of baseline characteristics to be summarized	Unchanged
	25b	Details of how baseline characteristics will be descriptively summarized	Unchanged
*Potential confounding covariates*	–	–	A description of potential confounding covariates and how these will be dealt with*
Section 6: Analysis			
Outcome definitions		List and describe each primary and secondary outcome including details of:	
	26a	Specification of outcomes and timings. If applicable include the order of importance of primary or key secondary end points (e.g., order in which they will be tested)	Unchanged
	26b	Specific measurement and units (e.g., glucose control, HbA_1c_ [mmol/mol or %])	Unchanged
	26c	Any calculation or transformation used to derive the outcome (e.g., change from baseline, QoL score, time to event, logarithm, etc)	Unchanged
Analysis methods	27a	What analysis method will be used and how the treatment effects will be presented	Unchanged*
	27b	Any adjustment for covariates	Unchanged
	27c	Methods used for assumptions to be checked for statistical methods	Unchanged
	27d	Details of alternative methods to be used if distributional assumptions do not hold, e.g., normality, proportional hazards, etc	Unchanged
	27e	Any planned sensitivity analyses for each outcome where applicable	Unchanged*
	27f	Any planned subgroup analyses for each outcome including how subgroups are defined	Unchanged*
Missing data	28	Reporting and assumptions/statistical methods to handle missing data (e.g., multiple imputation)	Unchanged*
Additional analyses	29	Details of any additional statistical analyses required, e.g. complier-average causal effect analysis	Unchanged
Harms	30	Sufficient detail on summarizing safety data, e.g. information on severity, expectedness, and causality; details of how adverse events are coded or categorized; how adverse event data will be analysed, i.e. grade ¾ only, incidence case analysis, intervention emergent analysis	*Only applies when interventions are studied.* Sufficient detail on summarizing safety data, e.g. information on severity, expectedness, and *associations*; details of how adverse events are *scored*; how adverse event data will be analysed and the follow-up time.*
Statistical software	31	Details of statistical packages to be used to carry out analysis	Unchanged
References	32a	References to be provided for nonstandard statistical methods	Unchanged
	32b	Reference to Data Management Plan	Unchanged
	32c	Reference to the Trial Master File and Statistical Master File	Reference to the *Study* Master File and Statistical Master File
	32d	Reference to other standard operation procedures to be adhered to	Unchanged

This table was adapted with permission from Gamble et al. [7]. *Italic* text highlights a rephrased word/sentence in the modified description for observational studies. An asterisk (*) indicates that a more elaborate description is present in our manuscript

The SAP of our observational study, the Simple Intensive Care Studies (SICS)-I, is presented as an example document (Additional file 1). This SAP was written as an add-on document to a pre-published protocol on clinicaltrials.gov [NCT02912624]. In absence of guidelines for observational study protocols, we used the first 20 items from the SPIRIT as a backbone for our observational study protocol (Additional file 2).

### Section 1: administrative information

The administrative information section in a SAP for an observational study is equally applicable to the content of a SAP for a randomised clinical trial. Item 1a and 1b were renamed while the content remained the same. For item 1b; a protocol of an observational study can be registered in a dedicated database (e.g. clinicaltrials.gov, researchregistry.com) alike randomised clinical trials [14, 17]. The description of item 4 was rephrased since in observational studies usually no interim analyses are planned (Table 1). All other items, names, and descriptions were left unchanged.

### Section 2: introduction

The introduction section in a SAP for an observational study is equal to the content of a SAP for a randomised clinical trial.

### Section 3: study methods

#### Sample size

Unlike randomised clinical trials that calculate a sample size to study an intervention effect taking power into consideration, the sample sizes of most observational studies are influenced by other factors (e.g. resources, time restrictions, convenience). Accordingly, most observational studies will have a given sample size and, if sufficiently large, affording enough power. The STROBE guidelines only expect authors to explain how the study size was arrived at [8], which may reduce the incentive to conduct sample size calculations for observational studies.

When there is a given sample size or if a sample size was not specified in the protocol, we suggest providing power considerations for the primary analysis of the observational study to limit random errors. The power considerations necessitate a definition of a minimally important difference or intervention effect in the presence of a given sample size. Any power calculation provides the chance of a type-II error (false negative findings), while a detectable difference may be clinically more informative. For example, it shows the minimal relative risk that can be detected with the specified power and sample size given a type I error probability α.

#### Framework

While causality can never be proven in observational studies, observed associations may fuel hypotheses that later can be tested in randomised clinical trials [18]. Although the vast majority of observational studies test for superiority, there are some that address equivalence and non-inferiority hypotheses [19–22]. Of course, confounding will always be present in any of these frameworks. Nevertheless, a SAP should describe whether the relevant hypothesis was assessed for superiority, equivalence or non-inferiority.

#### Statistical interim analyses and stopping guidance

Interim analyses are typically known to guide randomised clinical trials for early stopping due to benefit, harm or futility of tested interventions. Investigators are ethically obliged to conduct interim analyses to reduce study patients’ exposure to an inferior intervention. While there is usually no intervention component in observational studies which can be halted, there may be incentives to perform interim analyses for early stopping of continued (costly) data collections due to already clear observed associations or futility. Furthermore, observational studies may be subject to repeated testing of accumulating data, which needs adjustment of significance levels to reduce inflated type-I errors (false positive findings), such as those described by O’Brien & Fleming [23]. Such methods should be described in the SAP.

#### Timing of final analysis

A SAP for a blinded clinical trial should be published prior to unblinding the data or prior to the randomisation of the first participant in case of an open clinical trial. Likewise, a SAP for prospective observational studies should also be published before the first participant is included or at least all access to the database should be restricted. Randomised clinical trials that include blinding have a natural advantage that interventions can be coded during the statistical analyses. Such coding of interventions is usually not in question in observational studies, but it should be possible to mask the statistician by using coding for several covariates (at least dichotomous and categorical). Except for the study monitors, researchers should be unable to read the database before the study is finished or a SAP is written. If all study data were accessible to the researchers, a detailed SAP may still provide transparency on the intended analytic steps and may prevent ‘fishing’ for statistically significant predictors in analyses or other manipulations of the data. Any analysis that was not prespecified in the protocol and/or the SAP can only be explorative in nature, which should be described accordingly (i.e. exploratory or post-hoc analysis).

### Section 4: statistical principles

#### Multiplicity and type I errors

Multiplicity issues are similar in randomised clinical trials and in observational studies, but rarely addressed in the latter. Most observational studies ignore multiplicity issues by testing in multiple analyses at the same conventional *P* < 0.05 significance level. This increases the risks of a family wise error rate (FWER), that is the type I error of at least one false positive finding. Several methods have been suggested to adjust for multiplicity, such as those according to Bonferroni or Šidák [24, 25]. Even though International Conference on Harmonization of Good Clinical Practice guidelines recommend full Bonferroni adjustment [26], such an adjustment may be too conservative in correlated outcomes of observational studies [27].

For example, the SICS-I addresses six different primary outcomes spread out across 13 hypotheses [16]. Our outcomes cardiac output, acute kidney injury, and mortality are all affected by a patient’s haemodynamic status, so that most outcomes will probably be positively correlated. Since the Bonferroni adjustment assumes that outcomes are unrelated, we used an adjustment of our significance level that was pragmatic and probably more accurate. For more details we refer to the paper by Jakobsen and colleagues [28].

### Section 5: study population

#### Recruitment

It is necessary to elucidate the numbers of eligible and included patients of an observational study in a flow diagram, preferably according to the STROBE recommendations [29].

#### Potential confounding covariates

Results of observational studies can be seriously biased by confounding covariates. The randomisation procedure is used in randomised clinical trials to reduce the imbalance in observed and unobserved confounders between the allocated groups, although success can never be guaranteed [30]. The STROBE guidelines advocate to address the rate of confounding; however, it was recently shown that adherence to this statement is suboptimal [31]. A SAP could serve to predefine confounders, and how to address the expected rate of residual confounding by adjustment, or stratification.

Confounding variables are key important to address in observational studies. Usually, datasets of observational studies include large amounts of variables with many inevitably correlated to each other. For example, the SICS-I database contained 19 clinical examination findings which all reflected (a part of) the haemodynamic status of a patient. Next to expected confounding factors, the values of the variables can also be confounded by unmeasured factors such as environmental, genetic, or psychological influences. Therefore, we suggest to provide an a priori list of potentially confounding variables (both ‘measured’ and ‘unmeasured’) so that the reader is better able to assess the degree of residual confounding. Prelisting all potential variables and the approach to model building should be a main concern, if not the most important issue, in the SAP of observational studies.

### Section 6: analysis

#### Analysis methods

Analysis methods of clinical trials and observational studies are different, yet both study types are suspicious of selective reporting when no SAP is written [32]. Many decisions are needed during the analysis phase of an observational study and all that can be foreseen should be prespecified. An extensive description of the planned statistical analyses, all covariates, and all considerations need to be prespecified and detailed, which can only be done in a SAP. The usually short statistical analysis section of a manuscript does not allow a detailed explanation, nor can it guarantee the prespecified status of the analysis.

#### Sensitivity and subgroup analyses

The cost- and time-intensive nature of a randomised clinical trial necessitates a strict protocol in which all sensitivity and subgroup analyses are (usually) specified. In observational studies, these additional analyses are seldomly specified beforehand. A SAP is an opportunity for authors to prove that they had prespecified intentions of their sensitivity and subgroup analyses.

#### Missing data

Observational studies are particularly prone to missing data, but often do not address the mechanism of missing values. Complete case analyses in the presence of missing data are associated with bias, when data are not missing completely at random [33, 34]. Tests to identify the patterns and type of missing data, and the statistical methods to handle missing values should be described in a SAP. Examples include multiple imputations for data missing at random or worst-best and best-worst case scenarios for data missing not at random [34, 35].

#### Harms

Randomised clinical trials are costly and therefore often limited in size and length of follow-up, so that rare harms or late harms (e.g. after decades) remain undetected. Observational studies and post-marketing phase IV randomised clinical trials are much more suitable for detection of rare or late harms [35], of which the cardiovascular harms of clarithromycin in patients with stable coronary heart disease or cyclooxygenase-2 (cox-2) inhibitors are good examples [36, 37]. This item only applies to observational studies with a research questions focusing on an intervention effect. Our SICS-I cohort, for example, was not designed to study such associations.

### Applicability of SAP guidelines developed for randomised clinical trials to observational studies

Of the 32 proposed items by Gamble and colleagues (Table 1) [7], 30 items (94%) were also more or less directly applicable to a SAP for an observational study (Table 2). Some of these 30 items differ between trials and observational studies, mainly from a methodological point of view. We enclosed our SAP and study protocol in the supplements for illustrative purposes, so that it may serve as an example document for developing SAPs for other observational studies.

**Table 2:** Recommended content of statistical analysis plans for observational studies

Section/Item	Index	Description for observational studies
Section 1: Administrative information		
Title and study registration	1a	Descriptive title that matches the protocol, with SAP either as a forerunner or subtitle, and study acronym
	1b	Study registration number
SAP version	2	SAP version number with dates
Protocol version	3	Reference to version of protocol being used
SAP revisions	4a	SAP revision history
	4b	Justification for each SAP revision
	4c	Timing of SAP revisions in relation to planned repetitive analyses
Roles and responsibility	5	Names, affiliations, and roles of SAP contributors
Signatures of:	6a	Person writing the SAP
	6b	Senior statistician responsible
	6c	Chief investigator/clinical lead
Section 2: Introduction		
Background and rationale	7	Synopsis of study background and rationale including a brief description of research question and brief justification for undertaking the study
Objectives	8	Description of specific objectives and hypotheses, including secondary objectives
Section 3: Study methods		
Study design	9	Brief description of study design including type of study (e.g. case-control, cross-sectional or cohort study)
Power considerations	10	In case of an unspecified sample size, provide power calculations for (at least) the primary analysis or present a detectable difference with a specified power*
Framework	11	Superiority, equivalence, or noninferiority hypothesis testing framework, including which comparisons will be presented on this basis
Statistical interim analyses and stopping guidance	12a	Information on repetitive analyses specifying what repetitive analyses will be carried out and listing of time points
	12b	Any planned adjustment of the significance level due to repetitive analyses
	12c	Details of guidelines for stopping the study early
Timing of final analysis	13	Timing of final analysis, e.g., all outcomes analysed collectively or timing stratified by planned length of follow-up*
Timing of outcome assessments	14	Time points at which the outcomes are measured including visit “windows”
Section 4: Statistical principles		
Confidence intervals and P-values	15	Level of statistical significance*
	16	Description and rationale for any adjustment for multiplicity and, if so, detailing how the type 1 error is to be controlled*
	17	Confidence interval to be reported
Adherence and protocol deviations	18a	Definition of protocol deviations for the trial
	18b	Description of which protocol deviations will be summarized
Analysis populations	19	Definition of analysis populations, e.g., intention to treat, per protocol, complete case, safety
Section 5: Study Population		
Screening data	20	Reporting of screening data (if collected) to describe representativeness of study sample
Eligibility	21	Summary of eligibility criteria
Recruitment	22	Information to be included in the STROBE flow diagram*
Withdrawal/follow-up	23a	Level of withdrawal, e.g., dropouts after inclusion or refusal to be contacted for additional information
	23b	Timing of withdrawal/lost to follow-up data
	23c	Reasons and details of how withdrawal/lost to follow-up data will be presented
Baseline patient characteristics	24a	List of baseline characteristics to be summarized
	24b	Details of how baseline characteristics will be descriptively summarized
Potential confounding covariates	25	A description of potential confounding covariates and how these will be dealt with*
Section 6: Analysis		
Outcome definitions		List and describe each primary and secondary outcome including details of:
	26a	Specification of outcomes and timings. If applicable include the order of importance of primary or key secondary end points (e.g., order in which they will be tested)
	26b	Specific measurement and units (e.g., glucose control, HbA_1c_ [mmol/mol or %])
	26c	Any calculation or transformation used to derive the outcome (e.g., change from baseline, QoL score, time to event, logarithm, etc)
Analysis methods	27a	What analysis method will be used and how the treatment effects will be presented*
	27b	Any adjustment for covariates
	27c	Methods used for assumptions to be checked for statistical methods
	27d	Details of alternative methods to be used if distributional assumptions do not hold, e.g., normality, proportional hazards, etc
	27e	Any planned sensitivity analyses for each outcome where applicable*
	27f	Any planned subgroup analyses for each outcome including how subgroups are defined*
Missing data	28	Reporting and assumptions/statistical methods to handle missing data (e.g., multiple imputation)*
Additional analyses	29	Details of any additional statistical analyses required, e.g. complier-average causal effect analysis
Harms	30	Only applies when intervention effects are studied. Sufficient detail on summarizing safety data, e.g. information on severity, expectedness, and associations; details of how adverse events are scored; how adverse event data will be analysed and the follow-up time.
Statistical software	31	Details of statistical packages to be used to carry out analysis
References	32a	References to be provided for nonstandard statistical methods
	32b	Reference to Data Management Plan
	32c	Reference to the Study Master File and Statistical Master File
	32d	Reference to other standard operation procedures to be adhered to

This table was adopted from and created with permission from Gamble et al. [7]. An asterisk (*) indicates that a more elaborate description is present in our manuscript

Main reasons for ignoring two items (6%) were that these recommendations were specifically limited to trials, that is descriptions on randomisation and definition of adherence to the intervention.

## Discussion

Preregistration of protocols and SAPs for observational studies has been intensely debated [12–15, 38–48]. Opposing authors state that preregistration creates the false assumption that data are of high quality, would discourage publication of important accidental findings, and would delay these publications due to bureaucratic procedures [38–44]. Authors in favour argue that preregistration of protocols and SAPs distinguishes prespecified hypotheses from data dredging expeditions, ensures that methods can be replicated and findings confirmed, and reduces selective outcome reporting and publication bias [45–49]. Our present recommendations show the large similarities between SAPs for randomised clinical trials and observational studies and are parallel to our previous recommendations to publicly and transparently communicate all aspects of randomised clinical trials as well as observational studies from protocol to final results [1].

Observational studies are prone to confounding by indication, residual confounding, and flaws in data collection [50]. We argue that publication of a SAP increases the chance that hypotheses are adequately powered and investigated in the appropriate study population in which also all known confounders, mediators, and covariates are measured [46, 51]. Since credibility and replicability of findings in observational studies are a concern to many [11–15, 46, 52], the publication of a SAP allows better validation of findings in independent cohorts in an identical methodological and statistical manner. Furthermore, the concern that important findings will remain unpublished is less worrying than a lot of accidental findings getting published, creating confusion by researchers hunting hypothesis without real content. For the credibility of an ‘eye-catching’ finding to prevail, it still has to be replicated in a methodological sound study with an a priori hypothesis and an adequate statistical power. Irrespective of its potential benefits, publishing a SAP would at least do no harm and may be seen as an independent transparent determinant of validity.

## Conclusions

Both a protocol and a SAP in the public domain are equally helpful for both observational studies and randomised clinical trials [45]. By applying the guideline for the content of SAPs for clinical trials to our observational study we can conclude that more than 90% of the recommended content based on an extensive Delphi survey suits an observational study as well. There are only few adjustments needed for guidance of a SAP for observational studies when compared to a SAP for randomised clinical trials. In absence of SAP guidelines, we think that current recommend contents of SAPs for clinical trials could serve as a structure for SAPs of observational studies.

## Supplementary information

**Additional file 1:** statistical analysis plan of the Simple Intensive Care Studies-I.

**Additional file 2:** study protocol of the Simple Intensive Care Studies-I.

## Data Availability

Not applicable.

## Abbreviations

CONSORT: Consolidated Standards of Reporting Trials
FWER: Family wise error rate
PRISMA: Preferred Reporting Items for Systematic Reviews and Meta-Analyses
SAP: Statistical analysis plan
SPIRIT: Standard Protocol Items: Recommendations for Interventional Trials
STARD: Standards for the Reporting of Diagnostic Accuracy Studies
STROBE: Strengthening the Reporting of Observational Studies in Epidemiology
TRIPOD: Transparent reporting of a multivariable prediction model for individual prognosis or diagnosis
