# Supplementary material for: DEBATE-statistical analysis plans for observational studies
Source: BMC Med Res Methodol. 2019 Dec 9;19:233. doi: 10.1186/s12874-019-0879-5 (PMC6902479; doi:10.1186/s12874-019-0879-5)
Supplement: Supplementary file 1 — Additional file 1: statistical analysis plan of the Simple Intensive Care Studies-I. [file 12874_2019_879_MOESM1_ESM.pdf]

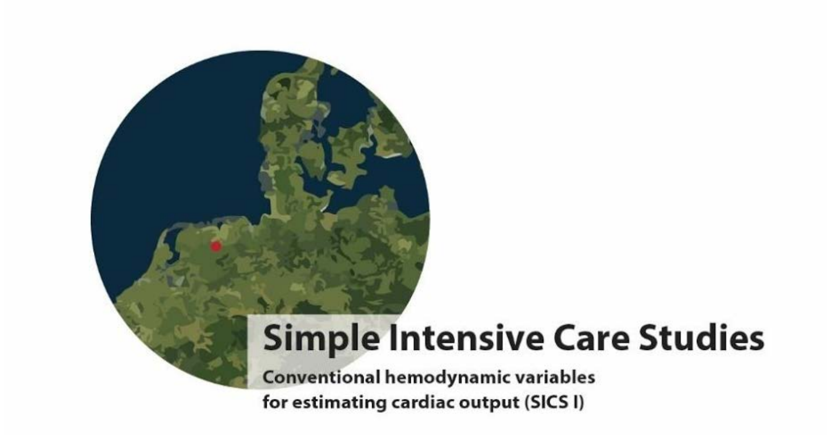

DETAILED STATISTICAL ANALYSIS PLAN (SAP)

1. Administrative information

1.1. Title, registration, versions and revisions

Full study title	A prospective observational study on the value of conventional hemodynamic parameters in estimating cardiac output and predicting mortality in critically ill patients
Acronym	Simple Intensive Care Studies-I (SICS-I)
Local project number	201500144
Clinicaltrials.gov number	NCT02912624
Study protocol version	1.1 (23 September 2016)
SAP version	1.0 (3 April 2018)
SAP revision history	None
SAP revision justification	-
SAP revision timing	Revision will be conducted after publishing the two main manuscripts. This revision will concern detailed SAPs for each sub-study.

1.2. Roles and responsibility

Author	Bart Hiemstra[1]
Statistician	Ilja M. Nolte[2]
Principle investigator	Iwan C.C. van der Horst[1]
Contributors and roles	Pim van der Harst[3]: revised the SAP<br>Frederik Keus[1]: contributed to the design and revised the SAP<br>Harold Snieder[2]: revised the SAP<br>Chris HL Tio[2]: main contributor to the section ‘missing data’<br>José N Alves Castela Cardoso Forte[1]: drafted the section ‘additional analyses: machine learning’<br>Jørn Wetterslev[4]: contributed to the design and revised the SAP. Main contributor to the sections ‘sample size, power, and detectable difference’ and ‘statistical significance and multiple testing’
Affiliations	1. Department of Critical Care, University of Groningen, University Medical Center Groningen, Groningen, The Netherlands<br>2. Department of Epidemiology, University of Groningen, University Medical Center Groningen, Groningen, The Netherlands<br>3. Department of Cardiology, University of Groningen, University Medical Center Groningen, Groningen, The Netherlands<br>4. The Copenhagen Trial Unit (CTU), Centre for Clinical Intervention Research, Copenhagen, Denmark

1.3. Signatures

We the undersigned, certify that we read this SAP and approve it as adequate in scope of the main-analyses of the SICS-I.

1.3.1. Author

Name:

………………………………………………………………………………

Date:

1.3.2. Statistician

Name:

………………………………………………………………………………

Date:

1.3.3. Principle investigator

Name:

………………………………………………………………………………

Date:

2. Introduction

2.1. Background and rationale

About one-third of all critically ill patients suffer from circulatory shock, which places them at increased risks of multi-organ failure, long-term morbidity, and mortality (1,2). Combinations of clinical, haemodynamic and biochemical variables are recommended for establishing the diagnosis and instigation of treatment (3,4). If necessary, more advanced and sequential haemodynamic assessments using critical care ultrasound (CCUS) as preferred modality are recommended (3-6).

Clinical examination in the critically ill comprises frequent measurement of heart rate, blood pressure, body temperature, skin perfusion, urine output and mental status (3). Daily use of clinical examination (in any patient) for diagnostic purposes contrasts with the limited number and quality of studies, so that the level of evidence for use of clinical examination in the critically ill is considered best practice (3). Previous studies have suggested different prognostic or diagnostic variables and many studies have analysed single or dual variable associations, while no research has evaluated their additional value on top of the accepted predictors (7). The reason for inconsistency of results in these studies potentially originate from several methodological flaws, including improper research design, lack of confirmation cohorts, and power and sample size issues.

The additive diagnostic and prognostic value of combinations of clinical, biochemical and haemodynamic variables remains to be established with a higher quality of evidence. These variables have never been evaluated collectively in a large, broad, prospective cohort of critically ill patients. Therefore, we established the Simple Intensive Care Studies I (SICS-I) with the aim to evaluate the diagnostic and prognostic value of a comprehensive selection of clinical and haemodynamic variables in the critically ill (7).

Prospective registration of protocols of observational studies are promoted to prevent outcome reporting bias (8,9). Likewise, prospective publication of a detailed statistical analysis plan (SAP) is encouraged to prevent data-driven analyses (9-11).

2.2. Objectives

2.2.1. Objectives and research questions

The objective of the SICS-I study was to establish a cohort with a dual aim: to evaluate the (1) diagnostic and (2) prognostic value of a comprehensive selection of clinical examination, haemodynamic and biochemical variables in the critically ill. More specific, the two research questions of the basic study were (1): which combination of clinical examination findings is associated with cardiac index measured

with CCUS? And (2): which combination of clinical examination, haemodynamic and biochemical variables is associated with 90-day mortality?

In the basic study of the SICS we collected a broad number of clinical examination, haemodynamic and biochemical variables, and used CCUS to only measure cardiac output. The infrastructure and design enabled (temporarily) addition of sub-studies in which additional variables were collected. Research questions of the sub-studies all address the overall aim of the SICS-I cohort (**table 1**).

2.2.2. Hypotheses

The hypothesis of research question 1, i.e. the diagnostic study, are:

- Null hypothesis: there is no true correlation between any single or a combination of clinical examination findings and cardiac index measured with CCUS
- Alternative hypothesis: cardiac index measured with CCUS is associated with one or a combination of clinical examination findings

The hypothesis of research question 2, i.e. the prognostic study, are:

- Null hypothesis: clinical examination, biochemical and haemodynamic variables are not associated with 90-day mortality
- Alternative hypothesis: clinical examination, haemodynamic and biochemical variables are associated with 90-day mortality

2.2.3. Scope

This SAP will be the guiding document for the analyses that will be conducted in the basic study. We intend to present the results of the two primary aims in separate manuscripts. All the aims and research questions of the sub-studies will be included in the appendix of this SAP, and we aim to present the SAPs of the sub-studies as an addendum in the future.

3. Study methods

3.1. General study design and plan

The SICS-I is a prospective cohort study which is conducted in the department of critical care of the University Medical Center Groningen (UMCG). The entire study was purely observational in design; no interventions were applied as part of the study protocol.

The protocol of this study was published on the website of the department of critical care of our hospital before the start of the study (Project number: 201500144) and registered at clinicaltrials.gov (NCT02912624). This analysis plan has been written while data collection was ongoing, but before full access to the study database. For our design paper, we only extracted baseline data and we did not have access to the validated outcome data.

3.2. Sample size, power and detectable difference

There are no previous studies which included combinations of clinical examination, haemodynamic and biochemical variables into one model for estimation of cardiac output and mortality. This makes it difficult to calculate sample size based on previous literature. Alternatively, we made an estimation of the power of our multivariable models given the set sample size of our cohort.

3.2.1. Diagnostic study

We are planning our main analysis with 1075 patients in which we will regress their values of cardiac index against clinical examination findings. For this power calculation, we used the clinical examination variables urine output and capillary refill time an example (7). In our design paper, the SD of cardiac index was 0.99 and the SD of urine output was 0.98, with a slope estimate of 0.040 obtained when cardiac index was regressed against urine output. The SD of capillary refill time was 2.4, with a slope estimate of -0.045 obtained when cardiac index was regressed against capillary refill time. If the true slope of the line obtained by regressing cardiac index against urine output or capillary refill time is 0.10, we will be able to reject the null hypothesis that this slope equals zero with probability (power) 0.81 for urine output, and 1.00 for capillary refill time. The type I error probability associated with this test of this null hypothesis is 0.015 (see paragraph 6.6 below).

3.2.2. Prognostic study

We are planning our main analysis with 1075 patients and take skin mottling as example for our power calculation: in our design paper, 46% of the patients has skin mottling. If we assume a similar proportion in our total cohort, we have 495 patients with skin mottling and 580 without. Pilot data from our design paper indicate that the 30-day mortality proportion among controls is 0.18 (7). If the true mortality proportion for patients with skin mottling is 0.27, we will be able to reject the null

hypothesis that the mortality proportion for patients with skin mottling and control patients are equal with probability (power) 0.84. The type I error probability associated with this test of this null hypothesis is 0.015. We will use an uncorrected chi-squared statistic to evaluate this null hypothesis.

3.3. Timing of final analysis

Data cleansing and CCUS image validation will be performed upon completion of the 90-day follow-up of the last patient included in the study. The final analysis will be conducted hereafter.

This statistical analysis plan was added to the study protocol at clinicaltrials.gov, before closure of the database and before any analyses had been conducted. Independent study monitoring was conducted in adherence to the Good Clinical Practice guidelines (10).

3.4. Timing of outcome assessments

Follow-up on all-cause mortality was conducted at 1 November 2017, i.e. 90-days after the inclusion of the last patient.

4. Statistical principles

4.1. Multiplicity

The diagnostic and prognostic basic study and each sub-study consist of one primary outcome and one or more secondary, exploratory outcomes. We will encounter multiplicity issues due to the multiple primary outcomes that are tested for significance in the same cohort. The SICS-I cohort addresses six different primary outcomes (table 2, last column); two primary outcomes of the basic study (diagnostic and prognostic), and four additional outcomes in the nine sub-studies.

We will apply an adjustment for multiplicity based on the total numbers of different primary outcomes tested. Our cohort mainly addresses haemodynamic research questions, so that most outcomes will probably be positively correlated. Therefore, a Bonferroni adjustment of the P-value might be too conservative. We chose for multiplicity adjusted thresholds by following the pragmatic approach stated by Jakobsen et al. (12). The authors suggested that the 'true' threshold lies somewhere between the unadjusted threshold (most often 0.05) and the Bonferroni adjusted threshold. Where in this interval the 'true' threshold is placed is dependent on the correlation between the outcomes, if two outcomes are perfectly correlated (a correlation coefficient of 1) no adjustment of the threshold for statistical significance is needed, if two outcomes are totally independent (a correlation coefficient of 0) a full Bonferroni adjustment is needed. Therefore, Jakobsen et al suggest "dividing the pre-specified P-value threshold with the value halfway between 1 (no adjustment) and the number of primary outcome comparisons (full Bonferroni adjustment)" as such an adjustment will come closer to the 'true' statistical significance level than the 'extreme thresholds' in a majority of situations (12). The corresponding formula is:

$$Threshold\ for\ significance = \frac{\alpha}{\left(\frac{m+1}{2}\right)}$$

Wherein:

- α is the unadjusted threshold for significance, usually 0.05
- m is the number of primary outcomes or tests (used) in the same cohort (in this case: six)

The threshold for significance in the SICS-I cohort will be:

$$Threshold\ for\ significance = \frac{0.05}{\left(\frac{6+1}{2}\right)} = 0.0143\ \approx 0.015$$

4.2. Statistical significance and confidence interval

As calculated in 4.1, we will consider a $P < 0.015$ as statistically significant for our primary outcomes. When we find a $0.015 \leq P \leq 0.05$, we will consider this association of dubious significance and will emphasise the increased chance of a type I error. Results will be presented with their values (e.g. regression coefficients, odds ratios, etc.) with 98.5% confidence intervals.

4.3. Adherence and protocol deviations

4.3.1. Definitions of protocol deviations

Protocol deviations are defined when the activities on a study diverge from the local institutional review board-approved protocol, however without significant consequences (13).

4.3.2. Protocol deviations to be summarised

We opted for the following subgroup analyses in our study protocol: different types of shock (distributive, obstructive, hypovolemic, cardiogenic), CVVH, heart failure by any cause, myocardial infarction, atrial fibrillation or surgery versus no-surgery patient groups.

We decided to replace these proposed subgroup analyses by the subgroups described in section 6.3. We believe that these subgroups agree better with the perspective from which a physician approaches a critically ill patient.

5. Study population

5.1. Screening data

Eligible patients who were not included will be compared to included patients by comparing their general characteristics (age, sex), and SAPS-II and APACHE-IV scores.

5.2. Eligibility

All eligible patients were included on their first day of ICU admission. Inclusion in the basic study consisted of a protocolised clinical examination and subsequent CCUS. The attending ICU physician estimated the expected duration of ICU treatment. Patients expected to stay beyond 24 hours who were eventually discharged within 24 hours were included in our main analyses.

5.2.1. Inclusion criteria

- Emergency admission
- Expected stay > 24 hours

5.2.2. Exclusion criteria

- Age < 18 years
- Planned admission (either after surgery or for other reasons)
- Unable to obtain informed consent, e.g. refusal, acute psychiatric disorders, mental retardation, serious language barriers
- Continuous resuscitation efforts or mechanical circulatory support

5.3. Recruitment

A flow diagram will be used to visualise the flow of patients. In this flow diagram, we will report the population from which the eligible patients were selected, reasons for exclusion of eligible patients, and how many CCUS images and measurements were validated (diagnostic study) or how many patients died (prognostic study). See **figure 1** for an example of our flow diagram.

5.4. Withdrawal/follow-up

5.4.1. Level and timing of withdrawal

The withdrawal rate of the SICS-I is below 2%, since it was an observational study in which no interventions were applied.

Following hospital regulations, patients or their legal representatives were informed and were excluded if they refused to participate. Withdrawal from our study occurred when informed consent was obtained from the patient’s legal representative, but the patient refused at a later time.

5.4.2. Reasons and details of withdrawal

Reasons for withdrawal or lost to follow-up will be reported in the manuscript and/or flow diagram. Our observational study consists of a one-time measurement (snapshot), so that drop-outs or lost to follow-up reasons are unrelated to the study.

Patients who were lost to follow-up are considered alive until their last outpatient visit or hospital discharge, and were censored thereafter.

5.5. Baseline patient characteristics

5.5.1. Collected baseline patient characteristics

The cohort study was designed to register a set of clinical examination, biochemical and haemodynamic variables in each included patient. We extracted baseline demographic data from the Dutch National Intensive Care Evaluation (NICE) registry and collected clinical data by protocolised clinical examination and CCUS. We obtained the biochemical values from arterial blood gas analyses closest to study inclusion. **Table 2** provides an overview of all collected variables and indicates for each variable whether it is categorised as a clinical examination, haemodynamic, or biochemical variable.

5.5.2. Descriptive summarization of baseline patient characteristics

We will list general patient characteristics in a baseline characteristics table. Data will be presented as mean with standard deviation (SD) when normally distributed or as median with interquartile range in case of skewed data. Dichotomous and categorical data will be presented in proportions. Normality of the data will be assessed using P-P plots, Q-Q plots, and histograms. Linearity will be assessed using scatter plots. Differences between continuous variables will be assessed using Student’s t-tests or Mann-Whitney-U test, depending on normality, whereas the Chi-squared test will be used for categorical values. For repeated measurements, we will use the paired t-test for normally distributed continuous data, the Wilcoxon signed-rank test for skewed continuous data, and the McNemar test for dichotomous data.

5.6. Assumed confounding covariates

The majority of variables measured in our study are inevitably correlated, as most relate to the haemodynamic status of a patient. While definitions have been recorded in the protocol, the values of the variables can be confounded by unmeasured factors, such as environmental, genetic, or psychological influences. Therefore, we provide an example of possible confounding variables and categorise these into ‘measured’ and ‘unmeasured’.

- Cardiac output and clinical examination of the central haemodynamics (i.e. heart rate, blood pressures, central venous pressure) are assumed to be confounded by:
    - Measured: body surface area (therefore we will use cardiac index), quality of measurements (therefore data will be validated), distributive shock as the underlying pathology, administration of inotropes and/or vasopressors, administration of propofol (negative inotropic effect), mechanical ventilation including ventilation pressures
    - Unmeasured: stress, pain, anxiety
- Urine production is assumed to be confounded by:
    - Measured: history of chronic renal failure, distributive shock as the underlying pathology
    - Unmeasured: total amounts of fluids administered
- AVPU score is assumed to be confounded by
    - Measured: sedation (propofol, midazolam)
- Clinical examination of peripheral perfusion (i.e. mottling, peripheral capillary refill times, peripheral temperatures) are assumed to be confounded by:
    - Measured: heating blankets, distributive shock as the underlying pathology, cardiac output, administration of inotropes and/or vasopressors, administration of propofol (vasodilation)
    - Unmeasured: regular blankets, environmental temperature, peripheral arterial disease.
- Mortality proportion is assumed to be confounded by:
    - Measured: age, comorbidities, and several other variables that are all embedded in the simplified acute physiology score II (SAPS-II) score
    - Unmeasured: cause of mortality (e.g., death due to multi-organ failure or failure to wean, a patient's or family's personal wishes regarding the extent of ICU treatment. This will, however, always be a mix of causes).

We acknowledge that there will be residual confounding in our dataset due to the presence of unmeasured confounding, some of which is listed above. However, the actual measured variables reflect daily practice and so, is assumed to reflect similar confounding in daily assessments of the haemodynamic status of ICU patients.

6. Analysis

6.1. Outcome definitions

6.1.1. Primary and secondary outcomes

The research questions and the design of the study have been published (7). We elaborate on the outcomes of the basic study below and described the primary outcomes of each sub-study in appendix 1.

The outcomes of research question 1, i.e. the diagnostic study, are:

- Primary: the association of a single or combination of clinical examination findings with cardiac index measured by CCUS
- Secondary: the diagnostic test accuracy of a single or a combination of clinical examination findings to diagnose a low, normal and high cardiac index
- Secondary: the association and diagnostic test accuracy of a single or combination of clinical examination findings with cardiac index in clinically different patient subgroups

The outcomes of research question 2, i.e. the prognostic study, are:

- Primary: the association of all measured clinical examination, biochemical and haemodynamic variables with 90-day mortality
- Secondary: the association of clinical examination, biochemical and haemodynamic variables with 7-day and 30-day mortality
- Secondary: the association of clinical examination, biochemical and haemodynamic variables that are not visible to caregivers with 90-day mortality
- Secondary: the association of clinical examination, biochemical and haemodynamic variables with 90-day mortality in clinically different patient subgroups

6.1.2. Measurement and calculation of outcomes

For the diagnostic study we calculated cardiac index, which was derived from cardiac output. Cardiac output has been measured with the cardiac probe M3S of M4S with default cardiac imaging setting of the General Electric Vivid-S6 mobile ultrasound machine. Two views were obtained: the parasternal long axis (PLAX) and the apical five chamber view (AP5CH). The PLAX was used as the primary view to measure the left ventricular outflow tract (LVOT) diameter. The AP5CH view was used to measure the velocity time integral (VTI) using the pulse wave Doppler signal in the LVOT. Cardiac output was calculated on the ultrasound machine according to the formula:

$$Cardiac\ output\ (\frac{L}{min}) = heart\ rate\ \cdot VTI \cdot \pi \cdot \left(\frac{1}{2} \cdot LVOT\right)^2$$

At a later time, the images and measurements were validated by technicians from an independent core laboratory, whom were blinded for all other measurements and outcomes.

We used cardiac index instead of cardiac output for interindividual comparisons. Cardiac index is the cardiac output adjusted for body surface area:

$$Cardiac\ index\ (\frac{\frac{L}{min}}{m^2}) = \frac{Cardiac\ output}{Body\ surface\ area}$$

Where body surface area was calculated with the DuBois formula (14):

$$Body\ surface\ area = 0.007184\ \cdot Weight^{0.425}\ \cdot\ Height^{0.725}$$

Cut-offs for a low cardiac index for critically ill patients are inconsistent (15). Haemodynamic criteria to diagnose cardiogenic shock vary from a cardiac index of 1.8 to 2.5 L/min/m^2 (16-19). A cardiac index below 2.2 L/min/m^2 is often used to diagnose a low cardiac output syndrome after cardiac surgery (20), whereas a large clinical trial used a cut off below 2.5 L/min/m^2 in patients with acute lung injury (19). These criteria, however, apply to patients with heart failure or after cardiac surgery. There currently is no consensus on how much cardiac index is low, normal or high for the critically ill patient. In the secondary outcome of our diagnostic study, we will both use a cut-off of 2.2 and 2.5 L/min/m^2 for a low, and a cut-off of 4.0 and 4.5 L/min/m^2 for a high cardiac index.

For the prognostic study we obtained follow-up on all-cause mortality from the municipal personal records database. Analysis of mortality will be performed using time-to-event data (patients were censored at 90-days of follow-up).

6.2. Analysis methods

6.2.1. Correlations

We will use the Pearson *r* correlation and Spearman correlation coefficient rho (*r*) to evaluate the degree of relationship between variables. For normally distributed variables, we will use the Pearson *r* correlation with 98.5% confidence interval upon checking for linearity and homoscedasticity, while in case of skewed, ordinal data, the degree of association between variables will be quantified using Spearman correlation coefficient rho (*r*). We will use Cohen's d to evaluate the correlation coefficient and assess the strength (or effect size) of the relationships, where a correlation coefficient between

0.10 and 0.29 will represent a small association, between 0.30 and 0.49 a medium association, and a coefficient of 0.50 and above a large association.

6.2.2. Continuous and dichotomous outcomes (diagnostic study)

We will conduct a least-squares linear regression analysis for continuous dependent variables and a logistic regression for dichotomous dependent variables. A univariable regression analysis will be conducted on 17 clinical examination findings with cardiac index as the dependent variable. A univariable regression analysis will be conducted on all variables and a $p < 0.25$ will be used for inclusion in the multivariable model. As there are no previous studies that include (a combination of) all available clinical examination variables into one model estimating cardiac index, we will not include any variable on a theory driven basis. We will construct the multivariable model using forward stepwise regression by adding blocks of variables. In case of a multivariable linear regression model, we will construct a kernel density plot to assess normality of the residuals and check the homogeneity of variance by plotting the residuals versus the fitted values. We will use the variance inflation factor (VIF) to check for multicollinearity; as a rule of thumb, we will assume multicollinearity when a variable has a VIF-value greater than 10. If the assumptions are not met, we will use an ordinal regression analysis.

6.2.3. Time-to-event data (prognostic study)

Analysis of mortality will be performed using time-to-event data (patients were censored at 90-days of follow-up). Categorical variables will be analysed using the log-rank test and continuous variables will be assessed using a univariable Cox proportional hazard regression analysis. Analysis of mortality proportion will be presented by Kaplan-Meier survival curves when independent variables are dichotomous or categorical. A univariable Cox regression analysis will be conducted on 22 clinical examination, haemodynamic and biochemical variables with 90-day mortality as the dependent variable. Covariates with a of $p < 0.25$ in the univariable analysis will be included in the multivariable model. We will include the SAPS-II score in our multivariable Cox regression model of 90-day mortality. The SAPS-II is a predictive score for in-hospital mortality and includes seventeen covariates among which age, haemodynamic and biochemical variables, and presence of metastatic or haematological cancer (21). The proportionality assumption will be tested by using the Schoenfeld and scaled Schoenfeld residuals. We will test the proportionality of the model as a whole and the proportionality for each predictor and reject proportionality in case of significant test findings ($p<0.05$). We will plot graphs of the scaled Schoenfeld assumption, where horizontal lines in the graph indicate that the proportionality assumption is not violated. We will also construct log-log plots, where two parallel lines indicate that proportionality was not violated. The goodness of fit of the final model will be evaluated

by using the Cox-Snell residuals. In our Cox regression models, at least 15 events are necessary for each variable included in the final model (22,23).

6.2.4. Validation (both)

Because of the fixed sample size of our study, we will use bootstrapping validation to assess the accuracy of our model. We will randomly select 1075 individuals from the dataset with replacement, build our model on the bootstrapped sample and validate it on the original data. We will repeat this process 100 times and include a variable into our final model if it was significant in at least 80 models. We will still emphasise the hypothesis-generating aspect of our findings. We aim to validate our findings in an independent cohort in the future.

6.3. Sensitivity and subgroup analyses

In the diagnostic study, we will conduct a sensitivity analyses on the subgroup of patients in which the quality of the cardiac output measurements by CCUS by the core laboratory is considered 'good'.

In the prognostic study, we will conduct a sensitivity analyses on different follow-up times of mortality: we will also use mortality at 7 and 30 days.

If the sample size permits, we will conduct subgroup analysis in different subpopulations. We will create the following subgroups in the basic study and test both our prognostic and diagnostic hypotheses on:

- Subgroup 1: subdivide the population into three groups: no shock, shock associated with a low cardiac output, shock associated with a high cardiac output.
- Subgroup 2: subdivide the population by underlying pathologies that could influence the haemodynamic measurements in a patient: Patients admitted due to cardiac arrest, heart failure, after liver transplantation or liver failure, central nervous system pathologies, and septic shock. We will identify clinical subgroups by using variables such as APACHE-IV admission diagnoses, confirmed infection, cardiac arrest from the Dutch National Intensive Care Evaluation (NICE) registry. The NICE registry contains good quality and complete data due to several assurance quality procedures (24). Patients with septic shock are identified using the quick Sequential Organ Failure Assessment (qSOFA) score combined with a confirmed infection according to the latest definition (25).

6.4. Missing data

6.4.1. Reasons for missing data

In our design paper, we extracted invalidated baseline data and obtained some insight in the missingness of our variables (7). We expect to have no missing data for the variables blood pressure, heart rate, urine output, central temperature, arterial haemoglobin and lactate levels. Some data will be unobtainable for the variables mottling score, capillary refill times and peripheral temperatures. Reasons for unobtainability included a dark or icteric skin colour (mottling and capillary refill times) and compression stockings (capillary refill time at the knee and peripheral temperature at the dorsum of the foot). Cardiac output measurements by CCUS were performed in all included patients. CCUS could not be performed in some patients due to various reasons obstructing the echocardiographic window, such as thoracic drains, post-surgical incisions, wounds or (subcutaneous) emphysema. Most probably, the missing values depend on other observed data (e.g. peripheral temperature could not be measured in patients under a warming blanket), and we consider these missing values as missing at random (MAR). If there is no correlation between the missing values and other observed data, i.e. Little's test is not statistically significant (P>0.05), missing values are considered missing completely at random (MCAR) (26).

6.4.2. Imputation method

If our missing values are missing at random, primary analyses will be performed with imputation for missing data using multiple imputations (MI). A threshold of up to 50% missing data will be considered acceptable for use of MI. Robustness of conclusions will be checked by secondary sensitivity analyses including available data and imputation of worst-best and best-worst case scenarios covering also missing not at random (MNAR) scenarios. We will use multiple imputation using the MI impute chained equation command in SPSS. We will compare the imputed values with the observed values to establish the validity of the imputed data; we will check whether the imputed values are realistic or if they require a cut-off to avoid unrealistic negative values. The imputation will be repeated 20 times and Rubin's rule will be used to combine variable estimates and standard errors (27).

If our missing values are MCAR or missingness is confined to the outcome variable, we will use complete case analysis for our primary analyses.

6.5. Additional analyses: diagnostic test accuracy

6.5.1. Relationship between clinical examination signs and cardiac index

There is currently no consensus on how much cardiac index is sufficient for the critically ill patient. Different studies used differing cut-offs for a low cardiac index (15). Therefore, we will use 2 cut-offs for both a low and high cardiac index:

1. Cut-offs for a low cardiac index: 2.2 and 2.5 L/min/m^2
2. Cut-offs for a high cardiac index: 4.0 and 4.5 L/min/m^2

We will conduct a logistic regression with the dichotomised cardiac index and an ordinal logistic regression with a categorical cardiac index as the dependent variable. A univariable analysis will be conducted on all dependent (clinical examination) variables and a $p < 0.25$ will be used for inclusion in a multivariable model. Calibration of our multivariable model will be checked with a Hosmer-Lemeshow test and by plotting observed proportions of cardiac index against the predicted risks of 10 equally sized groups. We will identify the cardiac index cut-off(s) where clinical examination has the best discriminative value based on the area under the receiver operating characteristic (ROC)-curves.

Based on the multivariable logistic regression findings, we will construct a risk score for cardiac index based on the coefficients in the model. Scores will be calculated by dividing all coefficients by the lowest coefficient or by transforming all coefficients so that the score is easy to count.

6.5.2. Diagnostic test evaluation

Depending on the optimal and number of cut-offs (i.e. low or normal cardiac index or low, normal and high cardiac index), we will display our diagnostic test in a 2 x 2 table, 2 x 3, or a 3 x 3 table (28,29). An example table can be found below. In a 3 x 3 table, data will be ordinal and we will give an indication of the agreement with the weighted kappa (30).

		**Cardiac index cut-offs**		
		Low (< 2.5)	*Normal (2.5 to 4.5)*	*High (> 4.5)*
**EXAMPLE: Capillary refill time**	*Prolonged*	True positive (a)	False positive (d)	False positive (d)
	Normal	False normal (b)	True normal (d)	False normal (b)
	Shortened	False negative (c)	False negative (c)	True negative (d)

Subsequently, we will calculate sensitivity, specificity, positive and negative predictive values and positive and negative likelihood ratios. Because this can only be calculated in 2 x 2 tables, we will cut the 3 x 3 table in low/normal and normal/high cut-offs:

		**Cardiac index below/above *x* L/min/m^2**	
		Yes	*No*
**Clinical examination finding(s) present**	*Yes*	True positive (a)	False positive (c)
	No	False negative (b)	True negative (d)

Explanation: *x* can be 2.2, 2.5, 4.0 or 4.5 L/min/m^2.

In addition, we will also calculate the 98.5% confidence intervals. Below we interpret each diagnostic test parameter for our diagnostic study.

- True positive: the number of patients with a low cardiac index who also had clinical examination signs indicating *hypo*perfusion.
- False positive: the number of patients without a low cardiac index, but with clinical examination signs indicating *hypo*perfusion.
- True negative: the number of patients without a low cardiac index and with clinical examination signs indicating *a normal* perfusion.
- False negative: the number of patients with a low cardiac index, but with clinical examination signs indicating *a normal* perfusion.
- Sensitivity: the probability of the presence of a low cardiac index when there were clinical examination signs indicating *hypo*perfusion.
- Specificity: the probability of the absence of a low cardiac index when clinical examination signs indicating *a normal* perfusion.
- Positive predictive value: the probability that a low cardiac index is present given the clinical examination signs indicate *hypo*perfusion.
- Negative predictive value: the probability that a low cardiac index is absent given the clinical examination signs indicate *a normal* perfusion.
- Positive likelihood ratio: the ratio between the probability of clinical examination signs indicating *hypo*perfusion among the patients with a low cardiac index, relative to the patients with the same test result but a normal cardiac index.

- Negative likelihood ratio: the ratio between the probability of clinical examination signs indicating *normal* perfusion among the patients with a low cardiac index, relative to the patients with the same test result but a normal cardiac index.
- ROC curve: "The receiver operating characteristic" curve. Grayscale image sensitivity as a function of (1 - specificity) for each possible cut-off. Most useful for comparison of two methods.
- Area under the ROC curve: displays the accuracy of the test and will be classified according to the following point system:
    - 0.90 to 1.0 = excellent
    - 0.80 to 0.90 = good
    - 0.70 to 0.80 = fair
    - 0.60 to 0.70 = poor
    - 0.50 to 0.60 = fail

6.6. Additional analyses: machine learning

6.6.1. Model development: algorithms, training and testing

We will use machine learning (ML) algorithms to generate hypotheses, validate observations of conventional models, and to unravel heterogeneity. Predictive modelling using ML algorithms requires the original data be split into two smaller sets, one for training and one for testing. We randomly split the original data into two groups with, respectively, 70 to 80% and 20 to 30% of the individuals. The split before further division of the training data into several folds for cross-validation virtually assures no information leakage is possible, making the training method virtually completely unbiased.

The training set will then be further divided into *k* similarly sized partitions (k-folds). By doing this, we divide the training set each time into *k* parts and then use each of these *k* parts once as testing dataset for the model trained on the other *k-1*. During this process, the hyper-parameters defining the model are optimized, with the performance measures (AUROC, accuracy, or a measurement of error, depending on whether the analysis is regression or classification) being presented as the average of those *k* runs of the algorithm. Additionally, this process can be repeated *r* number of times, where the average of *r* error terms obtained after performing *k*-fold cross validation *r* times is calculated.

This will be done for each of the algorithms, so as to determine the optimal values of all modifiable hyper-parameters of each model and maximize the models' performance metrics (usually the Area Under the Receiver Operating Characteristic curve (AUROC) for classification). When deemed

necessary, an additional post-hoc sensitivity analysis will be done to refine the parameters beyond what the tuning functions in R allow.

Three algorithms will primarily be used to develop the models: Gradient Boosted Machine, Support Vector Machine, and Random Forest. A summary of the necessary hyper-parameters for each of the algorithms is provided in table 3. Finally, an Ensemble Model will be built consisting of the best models build with each of the three algorithms. The choice for these three models is based on them having previously been shown to have similar, high performances in datasets with sparse data, despite the different structure of each algorithm (31). All three algorithms are not very sensitive to over-fitting (i.e. tend not to over-fit), but achieve this in different ways, where SVM is a disadvantage compared to the other two, since it attempts to minimize over-fitting for each kernel (i.e. type of model), but the user is still left to determine which kernel best fits the data, which can be error prone (32). In addition, both GBM and Random Forest are tree-based algorithms which allow for almost unprocessed data to be analysed. While SVM requires significant pre-processing, and is computationally slower than the other two, its high performance and the possibility to adapt a Radial Basis Function to almost all high-dimensionality problems makes it an easy-to-use, rather interpretable algorithm.

6.6.2. *Gradient Boosting Machine*

The Gradient Boosting Machine (GBM) algorithm is perhaps the strongest and the one with potentially the most interesting clinically-oriented properties. In GBM, new models are consecutively fitted to the training data set in order to provide a more accurate estimate of the outcome variable (33). By combining multiple decision trees, and increasingly weighting the “difficult to predict” events to a greater degree, GBM will fit *k* models (one per fold of the cross-validation process we defined earlier) to compute the error estimate, before making a final model using all of the data.

Our GBM model was tuned during training for four hyper-parameters: n.trees (the number of iterations to be generated by the algorithm), n.minobsinnode (the minimum number of observations in the terminal nodes of a tree (see Fig. 5)), interaction depth (defines the number of terminal nodes or leaves of a tree), and shrinkage (34). This parameter controls the learning rate of the algorithm by controlling the rate at which the boosting algorithm descends the error surface (35,36) .

6.6.3. *Support Vector Machine*

A Support Vector Machine (SVM) is a class of supervised learning algorithms often used in classification problems such as this. It classifies data points into two different classes (e.g. “Alive” or “Deceased”) by taking these points in a multidimensional space and separating them by means of the hyperplane that best differentiates between the two groups (37). Due to the great variation in range of our numeric

predictor variables in datasets with patient data, the data will be scaled and centred, to prevent attributes with a greater range dominating those with smaller ranges. Since SVMs don't allow for categorical variables, further processing is needed to encode all categorical variables into dummy variables (binary variables (0 or 1) which indicate the absence or presence of the effect of some parameter). After this, variables with zero or near-zero variance are excluded.

The SVM model will be tuned during training for two hyper-parameters: sigma (σ) and cost (C) (34). The best values for various pairs of exponentially growing C and sigma values will be determined during training by means of a "grid-search" using cross-validation (37,38). In addition, due to the expected dimensionality of the classification problem, we will use a Radial Basis Function (RBF) kernel to allow the hyperplane boundary between classes to be non-linear. Tuning of sigma is required in this context to determine the influence of a single training example on the overall prediction. For instance, an excessively large value would constrain the model back to linearity (37). Similarly, cost will control the misclassification tolerance by forcing the SVM towards a harder margin or allowing a smoother (i.e. softer) decision boundary and an increased probability of misclassification.

6.6.4. Random Forest (figure 3)

A Random Forest (RF) is an ensemble-based technique that attempts to minimise the limitations of classical decision trees by building multiple trees from a random subset of the original training data and considering only a random number of predictor variables at each split, instead of trying all the variables at every split, before aggregating their results (39-41).

As a learning method, it is more robust to overfitting than normal decision trees, which is especially important in relatively small training sets, and shows good predictive performance despite considerable noise (37). Furthermore, it requires virtually no pre-processing, running efficiently even on datasets with a large number of input variables, categorical and continuous.

Hyper-parameter optimization is done by defining a parameter-value grid with a wide range of trees and multiple values for *mtry*. The best model is selected either by comparison of the AUROCs obtained for each combination of these two parameters, or according to Kuhn and Johnson's threshold-based approach for datasets with imbalanced outcome classes (42).

6.6.5. Ensemble models

Lastly, an ensemble model will be built that combined the best model of each of the three base learner algorithms. Using an ensemble model is expected to increase model robustness, when compared to using individual models, which is achieved by incorporating the predictions from all the base learners.

The final predictions are then given twice, one label per type of ensemble: for the weighted ensemble, based on the weights defined for each model, the weighted predicted probabilities are calculated, and a label (e.g. "Alive" or "Deceased") is given to a case based on the defined threshold (of 0.50 in this case); for the majority vote ensemble, the label, and not the predicted probabilities, of each model are taken, and the class with at least two-our-of-three votes is attributed to a certain patient.

6.6.6. Model testing

All final models (for each algorithm or for the ensemble model) will be fitted to the testing dataset, resulting in a prediction of each patient's individual probabilities of belonging to either of the outcome classes ("Alive" or "Deceased"). Based on a defined threshold, these probabilities will then be converted to a binary label, and the AUROC and other additional performance measures for the models will be calculated for the testing dataset.

6.6.7. Principal Component Analysis

The robust implementation of all three algorithms used to model ICU mortality prediction in the SICS-I database in the caret package used in R, allows us to obtain an importance-based ranking of the variables, with respect to their ability to predict the outcome variable. This feature is potential applicable in clinical practice and provides an intuitive way to reduce the dimensionality (i.e. number of input variables). However, Principal Component Analysis (PCA) can be an even better tool to shed light on parameter prioritization for data-driven studies in the ICU.

PCA is an adaptive data analysis technique for reducing the dimensionality of large datasets, thus increasing interpretability, while simultaneously minimizing information loss. It identifies the largest sources of variation in a dataset and constructs a lower dimensional subspace of the data by creating new uncorrelated variables, or principal components (PCs), that successively maximize variance (43). To form each additional PC, it seeks a second linear combination that can explain the maximum of the remaining variance. For each PC, variable loadings are then provided, which represent the contribution of each original variable in explaining the variance in each of the PCs. Unlike predictive modelling, PCA is a descriptive tool, rather than inferential or predictive, which can partly substantiate or explain the predicted findings.

With iterative PCA, a variation on normal PCA, scores for both variables and individuals of complete or incomplete datasets are returned, providing an idea of the contribution of a certain variable, or a certain individual, to the total variance in that dataset (44). PPCA and BPCA are both iterative techniques with a probabilistic basis, which have been shown to perform better than traditional PCA when applied to data with missing values, especially as the missingness level increases (43). Using the

The R packages factoextra (45) and factoMineR (46), the PCA results are presented graphically by means of a graphic showing the distribution of individuals across a plane containing two PCs, a correlation circle graphic with the most explanatory variables or factors for each PC, and the PCA biplot showing a combination of both for clarity. The squared cosine (cos2) of the most relevant individuals and variables is also given, as is their percentual contribution (47).

Due to the heterogeneity in variable scaling (for example, the maximal urine per hour was 600 ml/hour, the highest value for haematocrit being 0.53, and 11.5 mmol/L for haemoglobin), all variables were centred and scaled (i.e. subtracting the variable mean, and dividing the value by the standard deviation), so as to standardise the variance of the dataset, allowing for comparability between variables with different scales and units (48).

6.7. Statistical software

Except for the predictive and PC analyses, statistical analyses will be performed using SPSS version 23 (IBM Corp. Released 2015. IBM SPSS Statistics for Windows, Version 23.0. Armonk, New York: IBM Corp) and Stata version 15.1 (StataCorp. 2017. College Station, Texas: StataCorp LLC).

All predictive and PC analyses were performed using R versions 3.3.3 and 3.5.0 Pre-Release (The R Foundation for Statistical Computing; Vienna, Austria).

7. Discussion

The overall aims of the SICS-I study are to address the diagnostic and prognostic value of clinical examination findings, haemodynamic variables, and biochemical values. This broad aim resulted in multiple research questions and hypotheses, which results in many variables to be tested repetitively. We drafted this SAP to avoid outcome reporting bias and data-driven results. This plan only addresses the hypotheses of the basic study which is, according to our sample size calculations, able to detect a 5% difference for the primary outcomes. Results of the sub-studies will be published as separate manuscripts and SAPs will be written before analysing their hypotheses.

We strive to eliminate inflated type I error rates by adjusting our primary outcomes for different confounders. We also strive to reduce the chance of a type I error due to multiplicity by pragmatically adjusting the P-value as proposed by Jakobsen et al. (12). This adjustment for multiplicity is based on the number of different primary outcomes of the basic study and sub-studies. In each manuscript, we will identify and discuss dubious significant findings. Although we will adjust for multiple testing, we will emphasise the hypothesis-generating aspect of results.

Our diagnostic hypotheses will be tested in two steps: first, we will investigate the association of cardiac index with clinical examination variables and second, we will investigate at which cardiac index the clinical examination findings of hypoperfusion become apparent. We aim to present a cut-off for low cardiac index relevant to critically ill patients in our secondary outcome, as current cut-offs for low cardiac index are based on patients with acute lung injury, heart failure or after cardiac surgery (16-20). Diagnostic test accuracies will be described for each finding or combinations of findings for our proposed cut-off of cardiac index. This secondary analysis, as well as the predefined subgroup analyses, will be considered exploratory and we will emphasise the need for validation in an independent cohort.

8. Conclusion

This SAP presents the principles of analysis of the SICS-I cohort and discusses its major methodologic and statistical concerns. We hope that the results of the SICS-I will be as transparent and robust as possible, so that we minimised the risk of outcome reporting bias and data-driven results.

9. Tables and figures

Figure 1. Flow diagram (example)

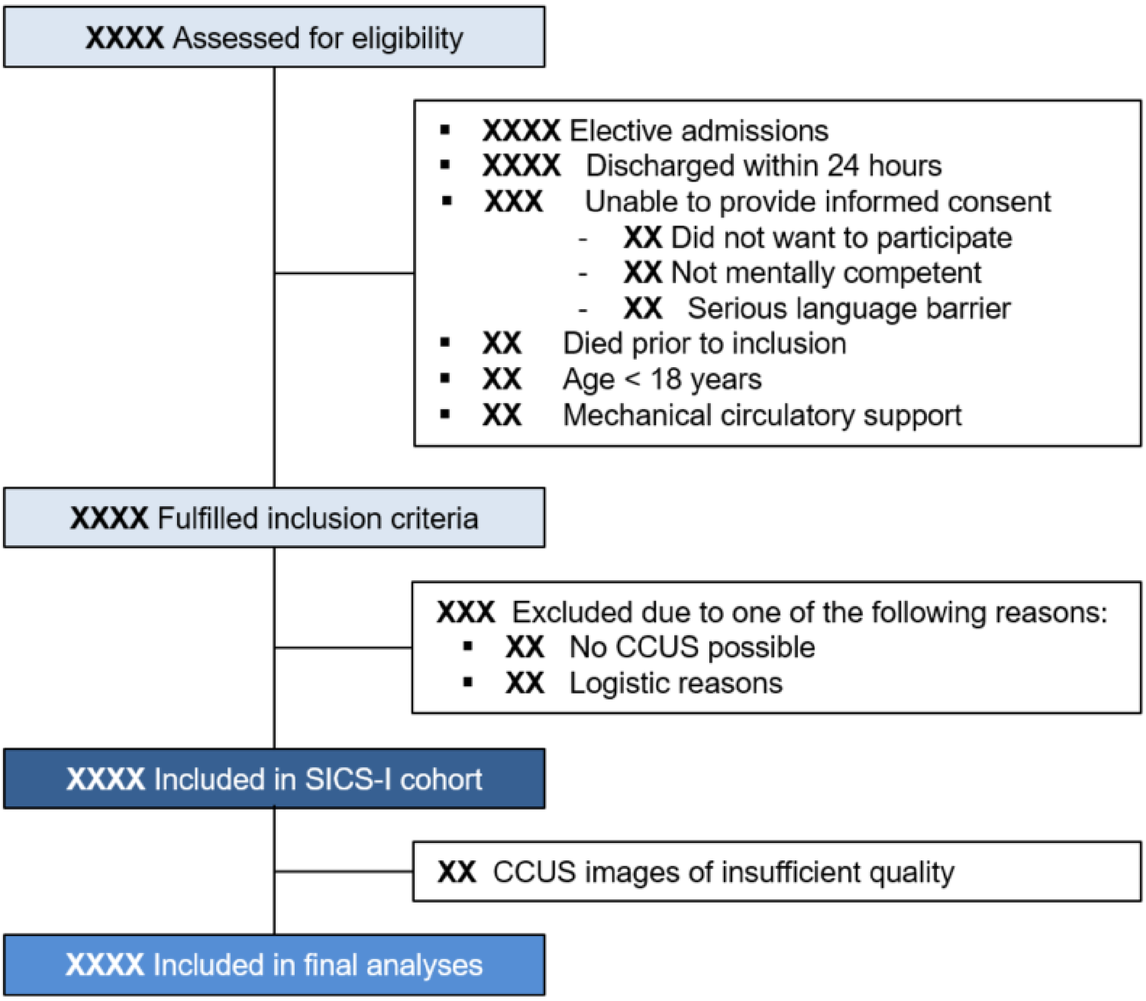

Figure 2. Representation of a linear and a radial hyperplane in a Support Vector Machine classifier.

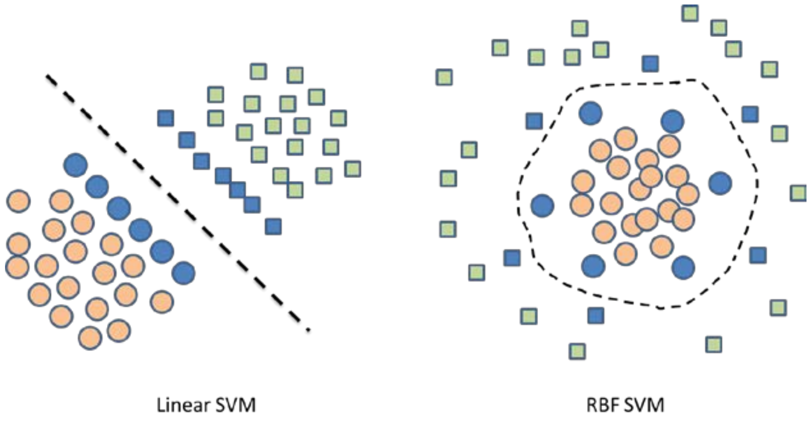

Class 1: squares, class 2: circles, and in blue the boundary cases.

Figure 3. Representation of a Random Forest classifier, with n trees

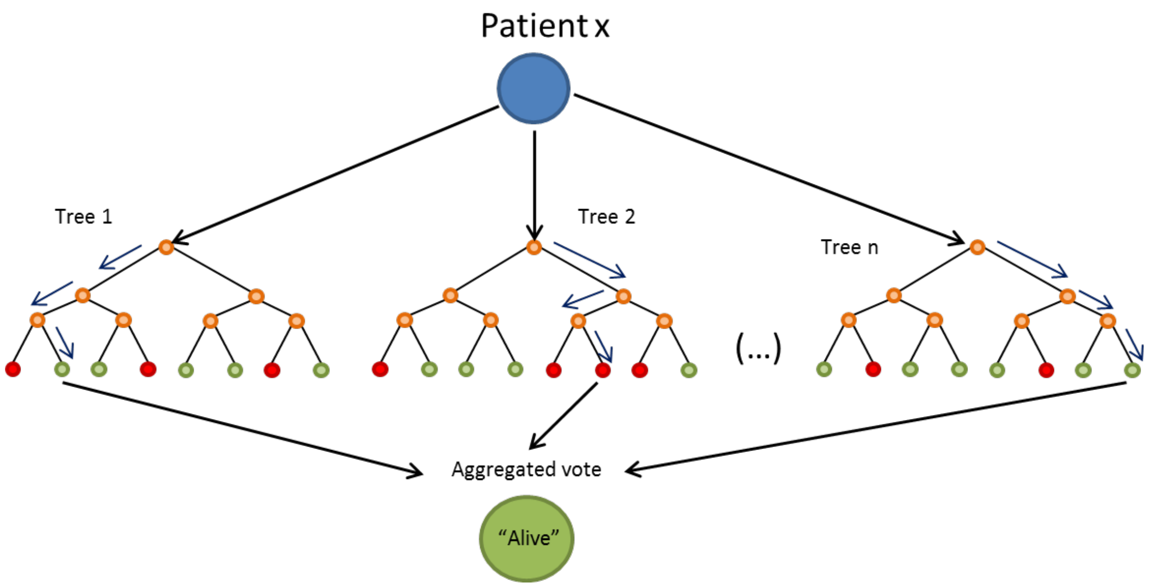

The circles represent the nodes, with the red and green circles signalling the terminal nodes, and the arrows show the "optimal split" computed for each node of each tree. The aggregated vote of all (n) trees is then combined and expressed as a final classification, for example, "alive".

Table 1. Specific research questions with add-on measurements

Short title	Research questions	Primary outcome
Basic study	Which combination of clinical variables obtainable through physical examination is associated with cardiac output measured by critical care ultrasonography (CCUS)?<br>Which combination of clinical and haemodynamic variables is associated with 7-day, 30-day and 90-day mortality?	Cardiac output<br>Mortality
**1.** NIRS	Which clinical examination, biochemical, and haemodynamic variables are associated with tissue (muscle) oxygen saturation (StO_2) measured by near-infrared spectroscopy (NIRS)?<br>Does the NIRS measurement at the knee have a better association with the clinical and haemodynamic variables compared to the NIRS measurement at the thenar muscle?<br>Is StO_2 measured by NIRS associated with 90-day mortality?	StO_2
**2.** Pulmonary ultrasound	What is the diagnostic accuracy of pulmonary oedema measured with pulmonary ultrasonography and auscultation for pulmonary crackles compared to pulmonary oedema diagnosed on a chest radiograph?<br>Is there a statistically and clinically significant difference in cardiac output between patients with and without a B-profile?	Pulmonary edema
**3.** PEEP-challenge	Is an increase in positive end-expiratory pressure (PEEP) associated with a decrease in cardiac output?	Cardiac output
**4.** RV-function & mortality	Is right ventricular (RV)-function measured by the tricuspid annular plane systolic excursion (TAPSE) and peak tissue Doppler systolic velocity in the tricuspid annulus (RV s’) associated with 90-day mortality?<br>Is RV-function measured by TAPSE or RV s’ associated with clinical examination and cardiac output?	Mortality
**5.** Abdominal flow	Is peripheral blood flow measured with CCUS associated with cardiac output?<br>Is a proxy for abdominal organ blood flow associated with acute kidney injury (AKI) or 90-day mortality?	Cardiac output
**6.** FloTrac**	What is the level of agreement between cardiac output measured by the FloTrac compared to cardiac output measured with CCUS?<br>Do the levels of agreement change when factors that might influence FloTrac measurements are present?	Cardiac output
**7.** Repeated measurements	Are changes in clinical examination findings over 24 hours associated with changes in cardiac output?<br>Are changes in clinical examination, haemodynamic and biochemical variables over 24 hours associated with 90-day mortality?	Cardiac output

**8.**	RV-function & AKI	Is RV-volume overload measured by tricuspid insufficiency and RV-diameters associated with acute kidney injury?<br><br>Are clinical examination, biochemical, and haemodynamic variables associated with the development of AKI in patients without known pre-existent chronic kidney disease?	Acute kidney injury
**9.**	Fluid responsiveness	What is the diagnostic accuracy of fluid responsiveness assessed by changes in end-tidal carbon dioxide (EtCO2), heart rate and blood pressure compared to fluid responsiveness assessed by the passive leg raising (PLR) test?<br><br>What is the diagnostic accuracy of fluid responsiveness assessed by a PLR test without lowering the head of the bed compared to fluid responsiveness assessed by the standard PLR test?	Cardiac output
**10.**	ARDS	What is the diagnostic accuracy of a B-profile measured with pulmonary ultrasonography compared to bilateral consolidations assessed on chest radiography for the diagnosis of acute respiratory distress syndrome (ARDS)?<br><br>Are B-lines measured with pulmonary ultrasonography associated with clinical examination, biochemical, and haemodynamic variables?	ARDS
**11.**	Myocardial strain	Is left ventricular (LV) and RV-myocardial strain measured with tissue Doppler imaging associated with 90-day mortality?<br><br>Is left ventricular (LV) and RV-myocardial strain associated with and conventional CCUS measurements?<br><br>What is the level of agreement between myocardial strain measured with tissue Doppler imaging and myocardial strain rate measured with speckle tracking?	Mortality

* B-profile: A B-profile is a strong indicator of pulmonary oedema and is present when three or more B lines are seen in at least three of the six BLUE points, or in two of the four lower BLUE points.

** FloTrac: the FloTrac (Edwards Lifesciences, Irvine, California, USA) is a pulse contour technique which analyses the arterial pressure waveform to compute stroke volume and cardiac output. The technique consists a dedicated pressure sensor (FloTrac) and a monitor to compute stroke volume and cardiac output (Vigileo).

Table 2. Overview of all variables measured in the basic study and the sub-studies.

**Variable**	**Basic study or substudy #**	**Baseline**	**After 24 hours**	**After 3 days**	**At 90 days**	**Retrospec-tive**
**Clinical examination**						
Examination date	B	X	X			
Examination time	B	X	X			
Gender	B	X				
Age	B	X				
Height	B	X				
Weight	B	X				
Respiratory rate	B, 7	X	X			
Heart rate and rhythm	B, 3, 6, 7, 9	X	X			
Intra-arterial blood pressures	B, 3, 6, 7, 9	X	X			
Non-invasive blood pressures	B	X				
Central venous pressure	B, 3, 6, 7, 9	X	X			
Urine output in 1 and 6 hours	B, 7	X	X			
Inotropic type, dose, speed	B, 6, 7	X	X			
Mechanical ventilation, ventilator settings	B, 3, 7, 9, 10	X	X			
AVPU score	B	X	X			
Sedative type, dose, speed	B	X	X			
Auscultation: souffles	B, 2, 7, 10	X	X			
Auscultation: crepitations	B, 2, 7, 10	X	X			
Auscultation: rhonchi	B, 2, 10	X	X			
Capillary refill: sternum	B, 7	X	X			
Capillary refill: index finger	B, 7	X	X			
Capillary refill: knee	B, 1, 7	X	X			
Temperature: subjective	B, 7	X	X			
Temperature: central	B, 7	X	X			

Temperature: peripheral at big toe and dorsum of foot	B, 7	X	X			
ΔTemp: central-to-peripheral	B, 7	X	X			
Mottling score at the knee	B, 1, 6, 7	X	X			
Peripheral circulation *estimation*	B, 7	X	X			
Pump function *estimation*	B, 7	X	X			
Degree of training	B, 7	X	X			
**Haemodynamic variables measured with CCUS**						
LVOT diameter	B, 6	X				X (validation in Corelab)
Peak flow velocity	B, 6, 7	X	X			X (validation in Corelab)
Velocity time integral	B, 6, 7	X	X			X (validation in Corelab)
Heart rate (echo)	B, 6, 7	X	X			X (validation in Corelab)
Cardiac output	B, 2, 3, 6, 7, 9, 10	X	X			X (validation in Corelab)
Cardiac output, every hour	6	X (during 6 hours)				
B-lines on 6 locations	2, 4, 7, 10	X	X			
TAPSE	2, 7, 8	X	X			
RV S’	2, 7, 8	X	X			
Common carotid artery diameter, flow	5	X				
Subclavian artery diameter, flow	5	X				
Common femoral artery, diameter, flow	5	X				
Abdominal flow	5	X				
RV basal diameter	8	X				
RV mid cavity diameter	8	X				
RV longitudinal diameter	8	X				
Tricuspid regurgitation peak velocity	8	X				
Left myocardial strain (rate)	11	X				

Septal myocardial strain (rate)	11	X				
Right myocardial strain (rate)	11	X				
**Other haemodynamic measurement devices**						
NIRS: StO_2 thenar & knee	1	X				
FloTrac: inclusion date and time	6, 7	X (during 6 hours)	X			
FloTrac: cardiac output every hour	6, 7	X (during 6 hours)	X (once)			
FloTrac: noradrenaline dose	6, 7	X (during 6 hours)	X (once)			
FloTrac: inotropic type, dose, and speed	6, 7	X (during 6 hours)	X (once)			
FloTrac: mottling score	6, 7	X (during 6 hours)	X (once)			
**Additional clinical variables**						
ICU admission date	B			X		
ICU admission time	B			X		
EMV score	B			X		
Admission reason	B			X		
Patient admission specifics	B			X		
Shock type	B			X		
Final diagnosis	B			X		
X-Ray measurements	B			X		X
ECG measurements	B			X		X
Respiratory distress	10			X		
Direct ARDS risk factors	10			X		
Indirect ARDS risk factors	10			X		
SOFA score						X
SAPS II score						X
APACHE II score						X

APACHE IV score						X
Total ICU stay						X
Medical history						X
**Biochemical variables**						
Arterial blood gas variables	B			X		
Serum leucocytes	B			X		
Serum hematocrit	B			X		
Serum thrombocytes	B			X		
Serum NT-proBNP	B			X		
Serum hs-troponin T	B			X		
Serum ASAT, ALAT	B			X		
Serum total and direct bilirubin	B			X		
Serum and urine creatinine	B, 8			X		
Serum and urine urea	B			X		
Serum and urine albumin	B			X		
Urine total volume and hours of collection	B, 8			X		
**Follow-up**						
Mortality in-ICU	All				X	
Mortality reason	All				X	
Mortality 7 day	All				X	
Mortality 30 day	All				X	
Mortality 90 day	All				X	

Table 3. Summary of the hyper-parameters of the three algorithms used for predictive modeling

Algorithm	Parameters	Definition
Gradient Boosted Machine	**n.trees**	Determines the number of trees (iterations) generated by the algorithm.
	**n.minobsnode**	Defines the minimum number of observations in the terminal nodes of a tree.
	**interaction depth**	Defines the number of terminal nodes or leaves of a tree.
	**shrinkage**	Controls the learning rate of the algorithm.
Random Forest	**ntree**	Defines the number of trees built by the algorithm.
	**mtry**	Defines the number of candidate variables randomly selected and tried at each split of a tree.
Support Vector Machine	**cost (C)**	Controls the misclassification tolerance.
	**sigma (σ)**	Defines how much a single training example influences the model (a higher sigma constrains the model towards linear).

10. References

(1) De Backer D, Biston P, Devriendt J, Madl C, Chochrad D, Aldecoa C, et al. Comparison of dopamine and norepinephrine in the treatment of shock. N Engl J Med 2010 Mar 4;362(9):779-789.

(2) Vincent JL, Marshall JC, Namendys-Silva SA, Francois B, Martin-Loeches I, Lipman J, et al. Assessment of the worldwide burden of critical illness: the intensive care over nations (ICON) audit. Lancet Respir Med 2014 May;2(5):380-386.

(3) Cecconi M, De Backer D, Antonelli M, Beale R, Bakker J, Hofer C, et al. Consensus on circulatory shock and hemodynamic monitoring. Task force of the European Society of Intensive Care Medicine. Intensive Care Med 2014 Dec;40(12):1795-1815.

(4) Vincent JL, De Backer D. Circulatory shock. N Engl J Med 2013 Oct 31;369(18):1726-1734.

(5) Cholley BP, Vieillard-Baron A, Mebazaa A. Echocardiography in the ICU: time for widespread use! Intensive Care Med 2006 Jan;32(1):9-10.

(6) Salem R, Vallee F, Rusca M, Mebazaa A. Hemodynamic monitoring by echocardiography in the ICU: the role of the new echo techniques. Curr Opin Crit Care 2008 Oct;14(5):561-568.

(7) Hiemstra B, Eck RJ, Koster G, Wetterslev J, Perner A, Pettilä V, et al. Clinical examination, critical care ultrasonography and outcomes in the critically ill: cohort profile of the Simple Intensive Care Studies-I. BMJ Open 2017;7(9):e017170.

(8) Williams RJ, Tse T, Harlan WR, Zarin DA. Registration of observational studies: is it time? CMAJ 2010 October 19;182(15):1638-1642.

(9) PLOS Medicine Editors. Observational studies: getting clear about transparency. PLoS Med 2014 August 26;11(8):e1001711.

(10) ICH Harmonised Tripartite Guideline: Guideline for Good Clinical Practice. J Postgrad Med 2001;47(3):199-203.

(11) Dwan K, Gamble C, Williamson PR, Altman DG. Reporting of clinical trials: a review of research funders' guidelines. Trials 2008 November 25;9:66.

(12) Jakobsen JC, Wetterslev J, Winkel P, Lange T, Gluud C. Thresholds for statistical and clinical significance in systematic reviews with meta-analytic methods. BMC Med Res Methodol 2014 Nov 21;14:120.

(13) Bhatt A. Protocol deviation and violation. Perspect Clin Res 2012 July 01;3(3):3485.100663.

(14) Du Bois D, Du Bois EF. A formula to estimate the approximate surface area if height and weight be known. 1916. Nutrition 1989 October 01;5(5):3.

(15) Hiemstra B, Eck RJ, Keus F, van der Horst, I C. Clinical examination for diagnosing circulatory shock. Curr Opin Crit Care 2017 Aug;23:article in press.

(16) Forrester JS, Diamond G, Chatterjee K, Swan HJ. Medical therapy of acute myocardial infarction by application of hemodynamic subsets (first of two parts). N Engl J Med 1976 December 09;295(24):1356-1362.

(17) Forrester JS, Diamond G, Chatterjee K, Swan HJ. Medical therapy of acute myocardial infarction by application of hemodynamic subsets (second of two parts). N Engl J Med 1976 December 16;295(25):1404-1413.

(18) Hochman JS, Sleeper LA, Webb JG, Sanborn TA, White HD, Talley JD, et al. Early revascularization in acute myocardial infarction complicated by cardiogenic shock. SHOCK Investigators. Should We Emergently Revascularize Occluded Coronaries for Cardiogenic Shock. N Engl J Med 1999 August 26;341(9):625-634.

(19) National Heart L, Wheeler AP, Bernard GR, Thompson BT, Schoenfeld D, Wiedemann HP, et al. Pulmonary-artery versus central venous catheter to guide treatment of acute lung injury. N Engl J Med 2006 May 25;354(21):2213-2224.

(20) Rao V, Ivanov J, Weisel RD, Ikonomidis JS, Christakis GT, David TE. Predictors of low cardiac output syndrome after coronary artery bypass. J Thorac Cardiovasc Surg 1996 Jul;112(1):38-51.

(21) Le Gall JR, Lemeshow S, Saulnier F. A new Simplified Acute Physiology Score (SAPS II) based on a European/North American multicenter study. JAMA 1993;270(24):2957-2963.

(22) Harrell FE,Jr, Lee KL, Matchar DB, Reichert TA. Regression models for prognostic prediction: advantages, problems, and suggested solutions. Cancer Treat Rep 1985 Oct;69(10):1071-1077.

(23) Concato J, Feinstein AR, Holford TR. The risk of determining risk with multivariable models. Ann Intern Med 1993 Feb 1;118(3):201-210.

(24) Arts D, de Keizer N, Scheffer GJ, de Jonge E. Quality of data collected for severity of illness scores in the Dutch National Intensive Care Evaluation (NICE) registry. Intensive Care Med 2002 May;28(5):656-659.

(25) Singer M, Deutschman CS, Seymour CW, Shankar-Hari M, Annane D, Bauer M, et al. The Third International Consensus Definitions for Sepsis and Septic Shock (Sepsis-3). JAMA 2016 Feb 23;315(8):801-810.

(26) Jakobsen JC, Gluud C, Wetterslev J, Winkel P. When and how should multiple imputation be used for handling missing data in randomised clinical trials - a practical guide with flowcharts. BMC Med Res Methodol 2017 December 06;17(1):1.

(27) White IR, Royston P, Wood AM. Multiple imputation using chained equations: Issues and guidance for practice. Stat Med 2011 February 20;30(4):377-399.

(28) Sappenfield RW, Beeler MF, Catrou PG, Boudreau DA. Nine-cell diagnostic decision matrix. A model of the diagnostic process; a framework for evaluating diagnostic protocols. Am J Clin Pathol 1981 June 01;75(6):769-772.

(29) Simel DL, Feussner JR, DeLong ER, Matchar DB. Intermediate, indeterminate, and uninterpretable diagnostic test results. Med Decis Making 1987 June 01;7(2):107-114.

(30) Watson PF, Petrie A. Method agreement analysis: a review of correct methodology. Theriogenology 2010 June 01;73(9):1167-1179.

(31) Jose´ Castela Forte J. Predicting long-term mortality with first week post-operative data after Coronary Artery Bypass Grafting using Machine Learning models. Machine Learning for Healthcare Conference 2017 /11/06;68:39-58.

(32) Gavin C. Cawley GCCCMPUEAACUK Nicola L. C. Talbot NLCTCMPUEAACUK School of Computing Sciences University of East Anglia Norwich, United Kingdom N7. On Over-fitting in Model Selection and Subsequent Selection Bias in Performance Evaluation. 2010;11(Jul):2079-2107.

(33) Friedman JH. Greedy function approximation: A gradient boosting machine. Ann Statist 2001 10;29(5):1189-1232.

(34) Kuhn M. Caret: Classification and regression training. ; 2013.

(35) Churpek MM, Yuen TC, Winslow C, Meltzer DO, Kattan MW, Edelson DP. Multicenter Comparison of Machine Learning Methods and Conventional Regression for Predicting Clinical Deterioration on the Wards. Crit Care Med 2016 February 01;44(2):368-374.

(36) Natekin A, Knoll A. Gradient boosting machines, a tutorial. Front Neurorobot 2013 December 04;7:21.

(37) Hastie T, Tibshirani R, Friedman JH. The Elements of Statistical Learning. 2nd ed. New York, NY: Springer-Verlag New York Inc.

(38) Hsu C-, Chang C-, Lin C-. A Practical Guide to Support Vector Classification. ; 2003.

(39) Liaw A, Wiener M. Classification and Regression by RandomForest. ; 2001.

(40) Breiman L. Random Forests. Machine Learning 2001 Oct;45(1):5-32.

(41) Boulesteix A, Janitza S, Kruppa J, König IR. Overview of random forest methodology and practical guidance with emphasis on computational biology and bioinformatics. WIREs Data Mining Knowl Discov 2012 November 1,;2(6):493-507.

(42) Optimizing Probability Thresholds for Class Imbalances. Available at: http://appliedpredictivemodeling.com/blog/2014/2/1/lw6har9oewknvus176q4o41alqw2ow. Accessed Mar 13, 2018.

(43) Kristen A Severson, Mark C Molaro, Richard D Braatz. Principal Component Analysis of Process Datasets with Missing Values. Processes 2017 Jan 1,;5(3):38.

(44) Dray S, Josse J. Principal component analysis with missing values: a comparative survey of methods. Plant Ecol 2015 May;216(5):657-667.

(45) Kassambara A, Mundt F. factoextra: Extract and Visualize the Results of Multivariate Data Analyses. 2017 -08-22;1.0.5.

(46) Lê S, Josse J, Husson F. FactoMineR: An R package for multivariate analysis. Journal of Statistical Software 2008;25(1):1-18.

(47) Abdi H, Williams LJ. Principal component analysis. WIREs Comp Stat 2010 July 1,;2(4):433-459.

(48) Zhang Z, Castello A. Principal components analysis in clinical studies. Ann Transl Med 2017 September 01;5(17):351.

(49) Hajian-Tilaki K. Sample size estimation in diagnostic test studies of biomedical informatics. J Biomed Inform 2014 April 01;48:193-204.

(50) Simel DL, Samsa GP, Matchar DB. Likelihood ratios with confidence: sample size estimation for diagnostic test studies. J Clin Epidemiol 1991;44(8):763-770.

(51) Flahault A, Cadilhac M, Thomas G. Sample size calculation should be performed for design accuracy in diagnostic test studies. J Clin Epidemiol 2005 August 01;58(8):859-862.

APPENDIX 1: sub-studies

This appendix elaborates on the study methods of the sub-studies of the Simple Intensive Care Studies-I (SICS-I). Per sub-study, we elaborate on the research question, variable of interest, timing of the measurement in the basic study, additional exclusion criteria, sample size calculations, and the analyses. on the sub-study procedure, additional exclusion criteria, and primary and secondary outcomes.

The conclusions based on the analyses in the sub-studies are explorative in nature and must all be confirmed in separate (external) validation cohorts.

1. Near infra-red spectroscopy

1.1. Research question

Which clinical examination, biochemical, and haemodynamic variables are associated with tissue (muscle) oxygen saturation (StO_2) measured by near-infrared spectroscopy (NIRS)?

1.2. Variable of interest

StO_2 was measured by NIRS with the Inspectra StO_2 tissue oxygenation monitor, model 650 (Hutchinson Technology, Inc., Hutchinson, Minnesota, USA). We used a 15-mm probe to measure the StO_2 at a depth of 14mm at two sites: the thenar eminence and the distal end of the vastus medialis muscle. The average StO_2 value was calculated over 30 seconds after one minute of signal stabilisation.

1.3. Timing of measurement

During inclusion in the basic study (one-time snapshot).

1.4. Additional exclusion criteria

Liver failure (increased bilirubin levels cause interferences with StO_2 measurements), patients with dark skin complexion (large quantities of melanin interfere with the near-infrared light emanated by the probe).

1.5. Sample size, power and detectable difference

We will conduct analysis of this sub-study with 32 patients, in which we will regress their values of NIRS against clinical examination findings such as central-to-toe temperature difference. Prior data indicate that the standard deviation of central-to-toe temperature is 4.1 with and estimated standard deviation of the regression errors of 23. If the true slope of the line obtained by regressing NIRS against central-to-toe temperature is 3.406, we will be able to reject the null hypothesis that this slope equals

zero with probability (power) 80%. The type I error probability associated with this test of this null hypothesis is 0.015.

1.6. Runtime

1 April 2015 until 5 August 2015.

1.7. Primary analysis

The association between clinical, biochemical and haemodynamic variables and tissue (muscle) StO_2 measured by NIRS at the knee.

1.8. Secondary analyses

The association between clinical, biochemical and haemodynamic variables and tissue (muscle) StO_2 measured at the thenar muscle.

The association between tissue (muscle) StO_2 measured by NIRS and 90-day mortality.

2. Pulmonary ultrasound

2.1. Research question

What is the diagnostic accuracy of pulmonary oedema measured with pulmonary ultrasonography and auscultation for pulmonary crackles compared to pulmonary oedema diagnosed on a chest radiograph?

2.2. Variables of interest

Pulmonary ultrasound was conducted with the cardiac probe M3S of M4S with default cardiac imaging and maximal frequency (3.6 MHz) setting of the General Electric Vivid-S6 mobile ultrasound machine. We measured the presence or absence of B-lines at the six locations specified in the BLUE-protocol. The presence of a B-profile was defined by three or more B lines observed in at least three of the six BLUE points, or in two of the four lower BLUE points.

Pulmonary oedema was diagnosed by the radiologist who reviewed chest radiographs as part of daily care. The radiologist was blinded for the variables collected in our study.

2.3. Timing of measurement

During inclusion in the basic study (one-time snapshot).

2.4. Additional exclusion criteria

Patients after pulmonary transplantation.

2.5. Sample size, power and detectable difference

Due to a set sample size we were able to estimate the precision of our estimate (i.e. the maximum marginal error). We calculated the maximum marginal error with the formulas of Hajian-Tilaki (49) and information from Simel et al. (50) and Flauhault et al (51). In our pilot data, the prevalence of pulmonary oedema diagnosed by chest radiography was 18.6%, and a B-profile measured with pulmonary ultrasonography had a sensitivity of 54.5% and a specificity of 93.8%. Based on 446 validated chest radiography images, a type-I error of 0.015 (with a corresponding Z-value of 2.17), and the abovementioned prevalence, sensitivity and specificity, the estimated maximal marginal error for sensitivity is 11.9% and 2.7% for specificity.

2.6. Runtime

Since 1 September 2015.

2.7. Primary analysis

The diagnostic test accuracy of a B-profile measured with pulmonary ultrasonography compared to pulmonary oedema diagnosed by chest radiography.

The diagnostic test accuracy of pulmonary crackles assessed with auscultation compared to pulmonary oedema diagnosed by chest radiography.

2.8. Secondary analysis

The statistically and clinically significant difference in CO between patients with and without a B-profile.

3. Positive end-expiratory pressure challenge

3.1. Research question

Is an increase in positive end-expiratory pressure (PEEP) associated with a decrease in cardiac output?

3.2. Variable of interest

During the PEEP-challenge, an additional 10 cm H_2O of PEEP was temporarily applied when supervised by the treating ICU physician. The PEEP was elevated for a maximum duration of 5 minutes during which the changes in cardiac output, heart rate, blood pressures and central venous pressure were recorded.

3.3. Timing of measurement

During inclusion in the basic study and after temporary PEEP application (repeated measurements).

3.4. Additional exclusion criteria

The attending ICU physician was not interested in fluid responsiveness at time of inclusion.

3.5. Sample size, power and detectable difference

We are planning a study with paired measurements in 25 patients. Prior pilot data indicate that the difference in the cardiac output of matched pairs is normally distributed with standard deviation 0.60. If the true difference in the cardiac output before and after application of PEEP is 0.42, we will be able to reject the null hypothesis that this response difference is zero with probability (power) 80%. The type I error probability associated with this test of this null hypothesis is 0.015.

3.6. Runtime

1 September 2015 until 3 January 2016.

3.7. Primary analysis

The association between PEEP increase and cardiac output.

3.8. Secondary analysis

None.

4. Right ventricular function and mortality

4.1. Research question

Is right ventricular (RV)-function measured by the tricuspid annular plane systolic excursion (TAPSE) and peak tissue Doppler systolic velocity in the tricuspid annulus (RV s') independently associated with 90-day mortality?

4.2. Measurement method

TAPSE and RV s' have been measured with the cardiac probe M3S of M4S with default cardiac imaging setting of the General Electric Vivid-S6 mobile ultrasound machine. Both measurements were obtained in the AP4CH view. TAPSE was assessed in M-mode, after placing the cursor on the junction of the tricuspid valve and the RV free wall. RV s' was assessed in the tissue velocity imaging mode highlighting the area of interest. The pulsed Doppler sample volume was placed at the tricuspid level of the RV free (i.e. lateral) wall and the longitudinal velocity of excursion was measured.

4.3. Timing of measurement

During inclusion in the basic study (one-time snapshot).

4.4. Additional exclusion criteria

None.

4.5. Sample size, power and detectable difference

In our design paper, we have 112 patients with an impaired TAPSE and 279 patients with a normal TAPSE (controls). Pilot data indicate that the 90-day mortality proportion among controls is 0.28 [7]. We will be able to detect true relative risks of 0.46 or 1.62 in patients with impaired TAPSE relative to controls with probability (power) 80%. The type I error probability associated with this test of the null hypothesis that this relative risk equals 1 is 0.015. We will use an uncorrected chi-squared statistic to evaluate this null hypothesis.

4.6. Runtime

Since 10 February 2016.

4.7. Primary analysis

The association between RV-function measured by TAPSE or RV s’ and 90-day mortality.

4.8. Secondary analysis

The association between RV-function measured by TAPSE or RV s’ and clinical examination findings and cardiac output.

5. Abdominal flow

5.1. Research question

Is peripheral blood flow measured with CCUS associated with cardiac output?

5.2. Variables of interest

Common carotid artery, subclavian artery, and common femoral artery flows have been measured with the linear probe 8L or 9L and default carotid setting of the General Electric Vivid-S6 mobile ultrasound machine. A proxy for abdominal flow was calculated by subtracting flow over both left and right carotid, subclavian and femoral arteries from the cardiac output.

5.3. Timing of measurement

During inclusion in the basic study (one-time snapshot).

5.4. Additional exclusion criteria

Obstruction of the desired window of peripheral artery flow (e.g. by a central venous or dialysis catheter, postsurgical incisions, wounds, etc.).

5.5. Sample size, power and detectable difference

We will conduct analyses of this sub-study with 59 patients, in which we will regress their values of cardiac output against peripheral flow measurements such as common carotid artery flow. Our pilot data indicate that the standard deviation of common carotid artery flow is 7.9, with an estimated standard deviation of the regression errors of 97. If the true slope of the line obtained by regressing cardiac output against common carotid artery flow is 5.4, we will be able to reject the null hypothesis that this slope equals zero with probability (power) 80%. The type I error probability associated with this test of this null hypothesis is 0.015.

5.6. Runtime

14 April 2016 until 17 August 2016.

5.7. Primary analysis

The association between peripheral blood flow measured at the common carotid, subclavian, and common femoral arteries and cardiac output.

5.8. Secondary analysis

The association between a proxy for abdominal organ blood flow and acute kidney injury (AKI) or 90-day mortality.

6. FloTrac

6.1. Research question

What is the level of agreement between cardiac output measured by the FloTrac compared to cardiac output measured with CCUS?

6.2. Variable of interest

Cardiac output has been estimated with the FloTrac (Edwards Lifesciences, Irvine, California, USA) and a monitor to compute stroke volume and cardiac output (Vigileo, Edwards Lifesciences, Irvine, California, USA). The FloTrac analyses the arterial pressure waveform to compute stroke volume and cardiac output. The estimated cardiac output was compared to the cardiac output measured with the General Electric Vivid-S6 mobile ultrasound machine.

6.3. Timing of measurement

Paired cardiac output measurements were conducted after admission every hour for 4 hours and once 24 hours after admission.

6.4. Additional exclusion criteria

Patients not requiring vasopressors and/or inotropes, an inadequate acoustic window for cardiac output measured by CCUS, absence of an arterial line, atrial fibrillation during inclusion, aortic and/or mitral valve disease.

6.5. Sample size, power and detectable difference

We are planning a study with paired measurements in 55 patients. Prior pilot data indicate that the difference in the cardiac output of both measurements devices is normally distributed with standard deviation 0.86. If the true difference in the cardiac output of both measurements devices is 0.39, we will be able to reject the null hypothesis that this response difference is zero with probability (power) 80%. The type I error probability associated with this test of this null hypothesis is 0.015.

6.6. Runtime

Since June 1st, 2016.

6.7. Primary analysis

The level of agreement between cardiac output measured by the FloTrac and cardiac output measured with CCUS.

6.8. Secondary analysis

The changes in levels of agreement when factors that might influence FloTrac measurements are present.

7. Repeated measurements

7.1. Research question

Are changes in clinical examination findings over 24 hours associated with changes in cardiac output?

7.2. Variable of interest

We repeated the measurements of the variables collected in the basic study, sub-study 2, and sub-study 4. We performed these measurements 24 hours (minimum 22 to maximum 26 hours) after the first measurement and calculated differences. The sign of the variable indicates whether a variable has either increased (positive number) or decreased (negative number).

7.3. Timing of measurement

During inclusion in basic study and 24 hours (minimum 22 hours to maximum 26 hours) thereafter.

7.4. Additional exclusion criteria

Patients not requiring vasopressors and/or inotropes, expected stay less than 48 hours.

7.5. Sample size, power and detectable difference

We are planning to conduct this sub-study with 100 patients and we will regress their values of difference in cardiac output changes over 24 hours against clinical examination findings such as central-to-toe temperature difference. Prior data indicate that the standard deviation of central-to-toe temperature is 4.1 with an estimated standard deviation of the regression errors of 1.35. If the true slope of the line obtained by regressing changes in cardiac output against central-to-toe temperature differences is 0.16, we will be able to reject the null hypothesis that this slope equals zero with probability (power) 80%. The type I error probability associated with this test of this null hypothesis is 0.015.

7.6. Runtime

Since 12 August 2016.

7.7. Primary analysis

The association between changes in clinical examination findings over 24 hours and changes in cardiac output.

7.8. Secondary analysis

The association between changes in clinical examination, biochemical, and haemodynamic variables over 24 hours and 90-day mortality.

8. Right ventricular function and acute kidney injury

8.1. Research questions

Is RV-volume overload measured by tricuspid insufficiency and RV-diameters associated with acute kidney injury (AKI)?

Are clinical examination, biochemical, and haemodynamic variables associated with the development of AKI in patients without known pre-existent chronic kidney disease?

8.2. Variable of interest

Right ventricle diameters and tricuspid regurgitation velocity have been measured with the cardiac probe M3S of M4S with default cardiac imaging setting of the General Electric Vivid-S6 mobile ultrasound machine. The measurements were obtained in the AP4CH view with a right ventricle centred view. AKI was established and classified following the kidney disease: improving global outcomes (KDIGO) criteria. Urine output and serum creatinine measurements from the first 72 hours of inclusion were analysed to establish and classify AKI for each patient.

8.3. Timing of measurement

During inclusion in basic study (one-time snapshot).

8.4. Additional exclusion criteria

None.

8.5. Sample size, power and detectable difference

In our design paper, we have 489 patients who developed AKI and 418 patients with a normal renal function (controls). Pilot data from our design paper indicate that the AKI proportion among controls is 0.54. We will be able to detect true relative risks of 0.80 or 1.20 in exposed patients relative to unexposed patients with probability (power) 0.80. The type I error probability associated with this test of the null hypothesis that this relative risk equals 1 is 0.015.

8.6. Runtime

Since 25 October 2016.

8.7. Primary analyses

The association between RV-volume overload measured by tricuspid insufficiency and RV-diameters and AKI.

The association between clinical, biochemical, and haemodynamic variables and the development of AKI.

8.8. Secondary analyses

The association between RV-volume overload measured by tricuspid insufficiency and RV diameters and 90-day mortality.

The association between clinical, biochemical, and haemodynamic variables and the development of AKI regardless of the presence of pre-existent chronic kidney disease.

9. Fluid responsiveness

9.1. Research question

What is the diagnostic accuracy of fluid responsiveness assessed by changes in end-tidal carbon dioxide ($EtCO_2$), heart rate and blood pressure compared to fluid responsiveness assessed by the passive leg raising (PLR) test?

What is the diagnostic accuracy of fluid responsiveness assessed by a PLR test without lowering the head of the bed compared to fluid responsiveness assessed by the standard PLR test?

9.2. Variable of interest

During the fluid responsiveness study, two different PLR tests were applied when supervised by the treating ICU physician. Every passive leg raising manoeuvre was conducted for a maximum duration of 60 seconds during which the changes in cardiac output, heart rate, blood pressures, central venous pressure, and $EtCO_2$ were recorded. Fluid responsiveness was diagnosed when cardiac output increased with 15% after the PLR-test. The PEEP-challenge was conducted in a similar manner as described in sub-study 3.

9.3. Timing of measurement

During inclusion in basic study (one-time snapshot).

9.4. Additional exclusion criteria

The attending ICU physician was not interested in fluid responsiveness at time of inclusion.

9.5. Sample size, power and detectable difference

Due to a set sample size we were able to estimate the precision of our estimate (i.e. the maximum marginal error). In our pilot data, the prevalence of fluid responsiveness was 45%, and $EtCO_2$ increase of 0.3 had a sensitivity of 83.3% and a specificity of 71.4%. Based on 20 included patients who underwent the PLR-test, a type-I error of 0.015 (with a corresponding Z-value of 2.17), and the abovementioned prevalence, sensitivity and specificity, the estimated maximal marginal error for sensitivity is 11.9% and 2.7% for specificity.

9.6. Runtime

Since 20 January 2017.

9.7. Primary analyses

The diagnostic accuracy of fluid responsiveness assessed by changes in $EtCO_2$, heart rate and blood pressure compared to the PLR test.

The diagnostic accuracy of fluid responsiveness assessed by a PLR test without lowering the head of the bed compared to the standard PLR test.

9.8. Secondary analyses

The association between a temporary PEEP-increase and cardiac output in fluid responders and fluid non-responders.

The diagnostic accuracy of a temporary PEEP-increase compared to the standard PLR test.

10. Acute respiratory distress syndrome

10.1. Research question

What is the diagnostic accuracy of a B-profile measured with pulmonary ultrasonography compared to bilateral consolidations assessed on chest radiography for the diagnosis of acute respiratory distress syndrome (ARDS)?

10.2. Variable of interest

ARDS will be defined according to the Berlin ARDS criteria: 1) presence of acute hypoxemic respiratory failure defined by a PaO2/FiO2 ratio < 300 mm Hg and PEEP ≥ 5 cm H_2O; 2) onset within one week of clinical insult or worsening respiratory symptoms; 3) bilateral consolidations on chest radiography or CT-thorax.

10.3. Timing of measurement

During inclusion in basic study (one-time snapshot).

10.4. Additional exclusion criteria

Included patients with no pulmonary ultrasound or chest radiography measurements, patients after pulmonary transplantation

10.5. Sample size, power and detectable difference

Due to a set sample size we were able to estimate the precision of our estimate (i.e. the maximum marginal error). In our pilot data, the diagnosis of ARDS was 5.8%, and the presence of a B-profile had a sensitivity of 88.5% and a specificity of 64.5%. Based on pilot data of 446 included, a type-I error of 0.015 (with a corresponding Z-value of 2.17), and the abovementioned prevalence, sensitivity and specificity, the estimated maximal marginal error for sensitivity is 13.6% and 20.4% for specificity.

10.6. Runtime

Since 1 September 2015. The research questions and hypotheses were incorporated at 1 February 2016.

10.7. Primary analysis

The diagnostic accuracy of a B-profile assessed with pulmonary ultrasonography compared to bilateral consolidations assessed on chest radiography for the diagnosis of ARDS.

10.8. Secondary analysis

The association between B-lines measured with pulmonary ultrasonography and clinical examination, biochemical and haemodynamic variables.

11. Myocardial strain

11.1. Research question

Are left ventricular (LV) and RV-myocardial strain measured with tissue Doppler imaging associated with 90-day mortality?

11.2. Variable of interest

Myocardial strain and myocardial strain rates have been measured with the cardiac probe M3S of M4S with default cardiac imaging setting of the General Electric Vivid-S6 mobile ultrasound machine. The measurements were obtained in the AP4CH window with a left ventricle and right ventricle centred view for left and right myocardial strain, respectively.

11.3. Timing of measurement

During inclusion in basic study (one-time snapshot).

11.4. Additional exclusion criteria

None.

11.5. Sample size, power and detectable difference

In a pilot study we included 51 patients of which 9 died. Pilot data from our design paper indicate that the 90-day mortality proportion among controls is 0.28 [7]. We will be able to detect a true relative risk of 3.0 in diseased patients subjects relative to patients who survived with probability (power) 0.80. The Type I error probability associated with this test of the null hypothesis that this relative risk equals 1 is 0.015.

11.6. Runtime

Since 18 March 2017.

11.7. Primary analysis

The association between LV- and RV-myocardial strain measured with tissue Doppler imaging and 90-day mortality.

11.8. Secondary analyses

The association between LV- and RV-myocardial strain imaging and conventional CCUS measurements.

The level of agreement between myocardial strain measured with tissue Doppler imaging and myocardial strain rate measured with speckle tracking.
