# Supplementary material for: DEBATE-statistical analysis plans for observational studies
Source: BMC Med Res Methodol. 2019 Dec 9;19:233. doi: 10.1186/s12874-019-0879-5 (PMC6902479; doi:10.1186/s12874-019-0879-5)
Supplement: Supplementary file 2 — Additional file 2: study protocol of the Simple Intensive Care Studies-I. [file 12874_2019_879_MOESM2_ESM.pdf]

Simple Intensive Care Studies

Conventional hemodynamic variables for estimating cardiac output (SICS I)

STUDY PROTOCOL

A prospective observational study on the value of conventional hemodynamic parameters in estimating cardiac output and predicting mortality in critically ill patients. Simple Intensive Care Studies I (SICS-I).

Protocol version 2.0, November 8th 2016

Short title: conventional hemodynamic parameters for estimating cardiac output

Acronym: SICS-I

Study coordinators:

Ruben Eck, M.D.

Bart Hiemstra, M.D.

Principal Investigator:

Iwan C.C. van der Horst

Department of Critical Care

University Medical Center Groningen

Hanzeplein 1, 9700 RB Groningen, The Netherlands

Groningen University, Groningen, The Netherlands

r.j.eck@umcg.nl

b.hiemstra01@umcg.nl

Table of content

[1-5] Administrative information 4
1 Title 4
2 Trial registration 4
3 Protocol version 4
4 Funding 4
5 Roles and responsibilities 4
a) Contributorship 4
b) Sponsor contact information 5
c) Sponsor and funder 5
[6-8] Introduction 6
6 Background and rationale 6
7 Purpose and objectives 7
8 Design 7
[9-14] Methods: Participants, interventions, outcomes 8
9 Study setting 8
10 Eligibility criteria 8
11 Outcomes 8
12 Basic study line and add-ons 9
13 Participant timeline 10
14 Sample size 10
15 Recruitment 11
16 Informed consent 11
[17] Assignment of interventions 12
17 Blinding (masking) 12
[18-20] Methods: Data collection, management, analysis 13
18 Data collection methods 13

19 Data management 19

20 Statistical methods 19

a) Outcomes 19

b) Additional analyses 20

c) Analysis population and missing data 20

d) Subgroup analyses 20

[21] Appendices 21

Appendix 1A: Overview of the add-ons and their starting date 21

Appendix 1B: Table of add-ons and their starting date 21

Appendix 2: Flow chart of data collection moments 22

Appendix 3: Variables 23

Appendix 3: Echocardiography protocol 31

Appendix 4: NIRS-protocol 35

Appendix 4: Pulmonary ultrasound 36

Appendix 5: Inclusion procedure (Dutch) 38

[20] References 45

[1-5] Administrative information

1 Title

A prospective observational study on the value of conventional hemodynamic parameters in estimating cardiac output and predicting mortality in critically ill patients.

2 Trial registration

Not applicable

3 Protocol version

09-12-2014	Version 1.0 (Original)
18-12-2014	Version 1.1
07-01-2015	Version 1.2
08-01-2015	Version 1.3
09-01-2015	Version 1.4
06-02-2015	Version 1.5
19-03-2015	Version 1.6
31-03-2015	Version 1.7
15-04-2015	Version 1.8
08-11-2016	Version 2.0

4 Funding

There will be no funding from third parties.

5 Roles and responsibilities

a) Contributorship

B. Hiemstra (study coordinator)
University of Groningen,
Department of Critical Care, University Medical Center Groningen
Tel: +31-6-16069231
Email: b.hiemstra01@umcg.nl

R.J. Eck (study coordinator)
University of Groningen,
Department of Critical Care, University Medical Center Groningen
Tel: +31-6-42514894
Email: r.j.eck@umcg.nl

I.C.C. van der Horst (PI),
University of Groningen,
Department of Critical Care, University Medical Center Groningen
Tel: +31-50-3610147 / +31-6-14698780
Email: i.c.c.van.der.horst@umcg.nl

F. Keus,
University of Groningen,
Department of Critical Care, University Medical Center Groningen
Tel: +31-50-3610147 / +31-6-52724683
Email: f.keus@umcg.nl

All authors contributed to the study protocol and approved the final protocol.

b) Sponsor contact information

University Medical Center Groningen
Hanzeplein 1, 9700 RB Groningen, Nederland
General phone number: +31 50 361 6161

c) Sponsor and funder

Not applicable

[6-8] Introduction

6 Background and rationale

Circulatory shock is a condition of generalized inadequate blood flow through the body, leading to insufficient tissue perfusion and inadequate delivery of oxygen and other nutrients, to the extent that tissues are damaged (1). We can distinguish four basic mechanisms of circulatory failure, caused by a scale of underlying illnesses: distributive, hypovolemic, obstructive and cardiogenic shock. The last three types are characterized by a low cardiac output and hypovolemia. Distributive shock is characterized by peripheral circulation failure, with a low systemic vascular resistance, a disturbed microcirculation and a high cardiac output. Frequently, these forms overlap (2).

Shock is a common problem in the intensive care unit (ICU) as it affects about one third of the patients (3). Septic shock appears to be the most common type, followed by cardiogenic and hypovolemic shock. The diagnosis of shock is based on clinical examination with use of well-known circulatory parameters such as blood pressure and heart rate; biochemical parameters such as lactate and direct (semi-)invasive measurement of cardiac output and other variables (2).

Since cardiac output is an important determinant of oxygen delivery, many different methods of measuring cardiac output have been suggested. These methods range from non-invasive to invasive measurements with central lining. The most invasive method, the pulmonary artery catheter (PAC) has long been considered the optimal form of monitoring cardiac output by using thermodilution. However, this technique is associated with adverse events, such as bleeding, and there is no clear evidence of improved outcome (4). Therefore, numerous other techniques have been proposed, ranging from systems that use the dilution technique but only require central venous and peripheral artery lines; to less invasive tools that estimate cardiac output based on the arterial pressure waveform; and to non-invasive echocardiography (5-7).

Despite technical advances, much remains unknown about the value of conventionally used hemodynamic parameters for estimating cardiac output. A distinction between macro- and microcirculatory parameters can be made (8,9). Commonly used macro-circulatory parameters are heart rate, systolic and diastolic blood pressure, mean arterial pressure and central venous pressure. Lactate is used as a proxy for microcirculatory status. Over the years several other measurements have been suggested to improve insight in the hemodynamics of a certain patient or a group of patients. Skin temperature, capillary refill, mottling score and urinary output are used for

hemodynamic assessment of the peripheral circulation and tissue perfusion (10-15). Most of these parameters have not been evaluated in a large prospective study and especially a combination of all these parameters has not directly been correlated to cardiac output.

More knowledge on the predictive value of all hemodynamic parameters in estimating cardiac output could assist physicians in earlier detection of impaired hemodynamics without the need for invasive or advanced methods. In this study we aim to evaluate all hemodynamic parameters in a large unselected population of critically ill patients and to correlate them to cardiac output.

7 Purpose and objectives

The purpose of this study is to create an infrastructure for a registry flexible to incorporate temporarily added specific research questions on the outcome of critically ill patients.

- The primary objective is the association between cardiac output (measured by transthoracic echocardiography) and hemodynamic parameters, such as heart rate, systolic blood pressure, diastolic blood pressure, mean arterial pressure, central venous pressure, skin temperature (and gradients), capillary refill time, mottling score, etc.
- The secondary objective is the association between hemodynamic parameters and 7 day, 30 day, and 90 day mortality.

8 Design

This study has a single-centre prospective observational design.

[9-14] Methods: Participants, interventions, outcomes

9 Study setting

The study will be conducted in the adult Intensive Care of the University Medical Center Groningen, a tertiary teaching hospital in the Northern part of the Netherlands. The UMCG has 4 ICU's to which all types of patients are admitted, however each ICU has their own interest: surgical, cardiothoracic, internal, and neurological. We unrolled our study at the surgical ICU ("ICV 4") only to detect start-up problems and assess the feasibility and burden of our research on patients. We included our first patient at March 2015. From May 2015 we started including all acutely admitted patients at the internal ICU ("ICV 3"). Acutely admitted patients at the cardiothoracic ("ICV 2") and neurologic ICU ("ICV 1") were added to our registry from February 2016 and March 2016, respectively.

10 Eligibility criteria

Inclusion criteria:

- Emergency admission
- Expected stay > 24 hours

Exclusion criteria:

- Age < 18 years
- Planned admission either after surgery or for other reasons
- Unable to obtain informed consent, e.g. refusal, suicide attempts due to acute psychiatric 'derailment', mental retardation or a language barrier

11 Outcomes

Primary outcome:

The primary outcome measurement is the association of hemodynamic variables with the cardiac output, measured by transthoracic echocardiography. If cardiac output cannot be estimated using transthoracic echocardiography for any reason whatsoever, then transoesophageal echocardiography can be an alternative method for estimating cardiac output. If transoesophageal echocardiography is considered contraindicated or does not result in an estimate for cardiac output, then that case is included, but the outcome is considered a missing data.

Secondary outcome:

The secondary outcome is the association of hemodynamic variables with mortality at day 7, day 30 and day 90.

12 Basic study line and add-ons

We constructed a basic hemodynamic profile of each included patient through a onetime physical examination combined with transthoracic echocardiography (TTE). The continuously running cohort study, i.e., registry, was designed with a basic set including all conventionally hemodynamic parameters. In addition, specific research questions necessitating more technically or advanced measurements could be temporarily incorporated on top of the continuously running set of parameters. This allowed for a flexible design in terms of specific research questions, planning, and efficiency of data collection. If a measurement proved to be feasible, i.e. seemed accurate and could be performed within a few minutes, the measurement was added to our basic research line.

To date, we initiated a total of ten add-ons of which five are currently still ongoing and two are being planned. Appendix 1 provides an illustrative overview of the current and planned add-on research questions.

Table 1. Overview of add-on studies

Sub study #	Research question(s)
**NIRS**<br>*Status: terminated*	What is the association of conventional hemodynamic variables to StO2 measured by NIRS?<br>Do kneecap NIRS-measurements associate better with the variables mentioned above than the standard thenar measurement?
**Pulmonary ultrasound**<br>*Status: ongoing*	What is the association of a B-profile measured with ultrasound and auscultating pulmonary crepitations with the diagnosis of interstitial lung edema by chest radiograph?<br>Is there a difference in cardiac output between the group with and without the presence of a B profile?
**PEEP-challenge**<br>*Status: terminated*	Does an increase in PEEP correlate with a decrease in cardiac output?
**RV function**<br>*Status: ongoing*	What is the association between RV-function assessed with TAPSE and RV S' of the tricuspid annulus and 90-day mortality?<br>What is the association between RV-function and conventional hemodynamic variables obtained from physical examination?
**Abdominal flow**	Is there a correlation between cardiac output and peripheral blood flow?

Status: terminated	Can we calculate a proxy for abdominal organ blood flow by subtraction of peripheral flow to head and extremities from the cardiac output?
**FloTrac**<br>*Status: ongoing*	What is the level of agreement between cardiac output measured by the FloTrac device compared to cardiac output measured with TTE?<br>Do the levels of agreement change when factors that might influence FloTrac measurements are present?
**Longitudinal**<br>*Status: ongoing*	What is the association of conventional hemodynamic variables with the cardiac output measured on two different points: within the first 24 hours of admission and 24 hours thereafter
**RV-dilatation & AKI**<br>*Status: ongoing*	Is right ventricular volume overload measured by tricuspid insufficiency and right ventricle diameter associated with acute kidney injury in ICU patients?
**Fluid responsiveness**<br>*Status: planned*	Do variations in end-tidal carbon dioxide and carotid peak systolic velocity induced by the PLR test predict fluid responsiveness?<br>Can continuous cardiac output monitoring through arterial pulse contour analysis be used in the evaluating the response to the PLR test?
**10. ARDS**<br>*Status: planned*	To follow.

Abbreviations: NIRS, near-infrared spectroscopy; StO2, peripheral tissue oxygen saturation; PEEP, peak end-expiratory pressure; RV, right ventricle; TAPSE, tricuspid annular plane systolic excursion, TTE, transthoracic echocardiography; AKI, acute kidney injury; ICU, intensive care unit; PLR, passive leg raising.

13 Participant timeline

Eligible patients will be included within 24 hours after their arrival on the Intensive Care Unit. After inclusion all study parameters will be obtained once through physical examination combined with transthoracic echocardiography. Mortality will be assessed at 7 days after admission. Additionally, mortality will be assessed at 30 and 90 days after admission. (See appendix 2)

14 Sample size

There are no previous studies with data on including a combination of all available hemodynamic variables into one model estimating cardiac output and mortality. This makes it difficult to calculate sample size. We will therefore make an estimation based on the number of ICU admissions per year. Each year 3000 patients are admitted to one of four ICU units. Approximately 1500 of these admissions are unplanned emergency admissions. We estimate that half of these unplanned admissions fulfill the inclusion criteria. This leaves 750 patients eligible for inclusion. However, we assume that we will not be able to include all eligible patients for logistic and practical reasons. Therefore we aim to include 400 patients per year. With emergency admission critical care mortality

approaching 25% this will enable us to include at least ten variables in the final model for predicting mortality (acknowledging that at least 10 events are necessary for each variable included in the final model (16)).

15 Recruitment

Inclusion of patients and measurements of variables (both conventional hemodynamic variables and ultrasound variables) will be performed by the study coordinator or a co-researcher under supervision and responsibility of the principle investigator. Informed consent will be obtained.

16 Informed consent

The local institutional review board (Medisch Ethische Toetsingscommissie, UMCG; METc M15.168207) approved the study. As part of our hospital research code the patient or legal representative was aware of the ongoing study and patients were asked to give informed consent to review their electronical patients charts for additional clinical data, such as laboratory measurements, discharge diagnoses and outcome. If the patient was (temporarily) incompetent, a legal representative was approached and the patients' consent was asked at a later time, once they had regained legal competence. If asking informed consent was deemed too burdensome for the legal representative, e.g. due to a worrisome prognosis or imminent death, we waived our inquiry for informed proxy consent. According to the local UMCG research code we were authorized to use anonymous observational data if obtaining informed consent is judged to be reasonably impossible to obtain. After January 1st, 2016, all observational anonymous patient data may be used for scientific purposes unless a patient explicitly objects to usage of his/her data.

[17] Assignment of interventions

No intervention is applied.

17 Blinding (masking)

Due to the observational nature of the study, neither the study coordinator or any of the co-researchers nor patients will be blinded to the measurements. All echocardiographic measurements will be validated by an echocardiography laboratory technician or cardiologist who will be blinded for all other measurements.

[18-20] Methods: Data collection, management, analysis

18 Data collection methods

Within 24 hours of ICU admission, all hemodynamic variables will be obtained through a onetime physical examination combined with transthoracic echocardiography. Other variables (i.e. lab values) will be obtained from the patient' file at a later moment. The rationale and specific details of measuring each variable are described extensively below.

For a flow-chart of data collection, see appendix 2. See appendix 3 for a complete overview of all variables. See appendix 4 for a detailed description of the echocardiography protocol. In the summation below, the '●' indicate that these measurements are part of the basic research line, whereas numbers indicate the sub-study/add-on number for which the new measurement was added.

Systemic circulatory variables:

- *Cardiac output (CO):* it will be measured by transthoracic echocardiography, performed by different trained researchers. Both cardiac output and cardiac index (i.e. cardiac output corrected for body surface area) will be calculated. (See appendix 4 for a detailed description of the echocardiography protocol).

- *Stroke volume (SV):* this will be automatically calculated by dividing cardiac output by heart rate, both measured by transthoracic echocardiography.

- *Heart rate (HR):* it will be recorded from the bedside electrocardiographic monitor. In case of an irregular rhythm (i.e. atrial fibrillation) we will use the mean heart rate over a minute. Apart from heart rate the presence of atrial fibrillation will be recorded.

- *Systolic blood pressure (SBP), diastolic blood pressure (DBP) and mean arterial pressure (MAP):* these will be obtained by intravascular measurement using an arterial line, which is part of usual care. To allow for comparison, these variables will also be measured using a sphygmomanometer. In the latter case, mean arterial pressure will be calculated using the following formula: MAP = (SBP + 2*DBP)/3

- *Central venous pressure (CVP):* this will be recorded in case a central venous line is present in the internal jugular or subclavian vene.

Micro- and peripheral circulatory variables:

- *Capillary refill time (CRT):* this will be measured after 15 seconds of exerting firm pressure preferably on the sternum, the distal phalanx of the index finger, and on the central part of the knee. The original upper limit of a normal CRT was considered to be 2 seconds by Champions' Trauma score (17). However Schriger and Baraff examined CRT in a healthy population and discovered it to be age and temperature dependent, with an upper limit for healthy older adults of 4.5 seconds (18). In a recent study Ait-Oufella et al found that an index CRT upper limit of 2.4 seconds is predictive of 14 day mortality in septic shock patients. (13) We will therefore both use a cut-off value of 4,5 seconds and a continuous measure of CRT.

- *Skin temperature (Tskin):* this will be measured subjectively and objectively. The subjective measure will be conducted by palpating the patient's extremities. A distinction between either 'warm' or 'cold' will be made using the dorsal surface of the hands of the examiner. Patients will be considered to have 'cold' skin extremities if all examined extremities are considered cool, or if only the lower extremities are cool despite warm upper extremities.

To objectify the skin temperature, the use of a central-to-peripheral and peripheral-to-ambient temperature difference (respectively dTc-p and dTp-a) or the forearm-to-finger skin-temperature gradient (Tskin-diff) have been proposed in literature (10,11,19,20). We will make use of:~~ two different measurements:~~

- *Central-to-peripheral temperature difference (dTc-p):* to measure this difference we will compare bladder temperature, as measured by a bladder thermistor catheter, with foot temperature, measured by a skin probe (DeRoyal Skin Temperature Sensor product nr 81-010400EU) on either the left or the right big toe, as well as the left or right dorsum of foot. We will use bladder temperature as a surrogate for central temperature, and toe temperature as a peripheral measure. In literature a temperature difference of either 5°C or 7 °C is generally used as an upper limit (11,21,22). We will therefore regard values higher than 7°C as abnormal.
- ~~*Forearm-to-finger gradient (Tskin-diff):* we will also measure this gradient, since it has been validated by Rubinstein et al as an accurate measure of thermoregulatory peripheral~~

vasoconstriction.(23) Tskin-diff will be measured using two skin probes, one attached to the index finger and the other to the radial side of the forearm, mid-way between elbow and wrist. Based on previous studies we choose a Tskin-diff of 0°C as cut-off point, larger values will be regarded as an indication of peripheral vasoconstriction. (11,24)

- *The mottling score:* this score was described by Ait-Oufella et al in 2011 (14). Mottling is the patchy discoloration of the skin caused by microcirculatory dysfunction. It usually involves the area around the knee. The Mottling score ranges from 0 -5, depending on the extensiveness of the mottled area. A score of 0-1 is regarded mild, 2-3 moderate and 4-5 severe (also see appendix 3).

- *Urine output (ml/kg/h):* this is also measured as part of regular care. We will use both the urine output over the hour before examination and the mean urine output per hour, calculated using the six hours prior to the physical examination. If these data are unavailable, the mean urine output of the previous hour(s) will be calculated depending on the available data. In patients with pre-existing renal failure the urinary output will not be used.

1. *Near infrared spectroscopy (NIRS) (April 1st, 2015 until August 5th, 2015):* NIRS measurements were made using InSpectra™ tissue oxygenation monitor, model 650 (Hutchinson Technology, Inc., Hutchinson, MN). Due to budgetary reasons purchasing an InSpectra 650 by the ICU-research department was impossible. The InSpectra 650 was borrowed from the department of Anesthesiology and inclusion of patients depended on availability at time of inclusion. In eligible patients, we evaluated StO2 measurements on both knee and thenar. The average StO2 value was calculated over 30 seconds after one minute of signal stabilization.

Additional ultrasonic measurements:

2. *Lung ultrasound (per September 1st, 2015):* we acquired lung ultrasound at six different positions according the BLUE-protocol (25). The presence of B-lines was noted according a grading system and a B-profile was established. A detailed protocol is provided in Appendix 5.
3. *Peak end-expiratory pressure (PEEP) challenge (September 1st, 2015 until January 3rd, 2016):* an additional 10 cm H_2O of PEEP was temporarily applied when supervised by the treating intensivist. PEEP was elevated for a maximum duration of 5 minutes during which the new

hemodynamic variables were recorded. This duration of elevation was chosen in accordance with the protocol for the PEEP challenge designed and used by Geerts et al (26). We recorded the hemodynamic variables at the new PEEP level: *heart rate (beats per minute), systolic blood pressure (mmHg), diastolic blood pressure (mmHg), mean arterial pressure (mmHg), central venous pressure (mmHg) and cardiac output (L/min).*

4. *Tricuspid annular plane systolic excursion (TAPSE)* (per February 10th, 2016): we assessed right ventricular function by measuring the TAPSE. In literature cutoffs for TAPSE differ from 13.5 mm to 18.5 mm. Following the American Society of Echocardiography guidelines we will regard TAPSE values lower than 16 mm as abnormal (27).
4. *Systolic excursion velocity (RV s')*: (per February 10th, 2016): According to Rudski et al measurements of both TAPSE and RV s' provide a reliable assessment of right ventricular function (27). Similar to TAPSE cut-offs for RV s' differ (7.3 cm/s to 12.3 cm/s for RV s'). According to the American Society of Echocardiography guidelines we will regard RV S' values of 10 cm/s or lower as abnormal (27).
5. *Common carotid artery, subclavian artery, and common femoral artery flow (April 14th, 2016 until August 17th, 2016)*: for sub-study 5 we aimed to measure a proxy for abdominal flow by substracting flow over both left and right carotid, subclavian and femoral arteries from the cardiac output. Flow over these arteries could be determined by measuring the *diameter of the artery (mm)* and the average *velocity of blood flow (ml/min)* in the abovementioned arteries.
6. *FloTrac (per June 1st, 2016)*: The FloTrac (Edwards Lifesciences) measures *cardiac output (L/min)* by analyzing the arterial pulse waveform. Its accuracy was validated by concurrently measuring the cardiac output from the FloTrac device at the same time the blood flow through the ventricular outflow tract was measured with echocardiography. Paired cardiac output measurements were performed hourly after admission for 4 hours and once 24 hours after admission. Factors that might influence FloTrac cardiac output measurements (28,29) were also recorded: *inotropics usage* and *echocardiography image quality (poor, suboptimal, optimal).* In addition, we also noted the *mottling scores* and dosages of *inotropics (ug/kg/min)*.
7. *Right ventricular volume status (per October 25th, 2016):* we will estimate right ventricular volume load by tricuspid insufficiency and right ventricular diameter according to Rudski et al (27).

8. *Tricuspid regurgitation velocity (per October 25th, 2016):* we will assess the presence of tricuspid regurgitation by measuring the velocity of retrograde flow in the tricuspid valve. The cut-offs for tricuspid valve insufficiency is set at 2.4 m/s according to Rudski et al (27).

Other variables recorded at bedside:

- *Respiratory rate:* this will be recorded of the bedside electrocardiographic monitor. If a patient is on mechanical ventilation, see below.
- *AVPU scale:* this can be used to obtain a quick impression of a patient's state of consciousness and consists of the options: 'Alert', 'responsive to Voice', 'responsive to Pain' and 'Unresponsive.' It is often applied in the emergency room and the general wards as part of the 'MEWS' (Modified Early Warning Score) (30,31). We will score the AVPU scale as a separate item.
- *Cardiac murmurs:* we will auscultate the heart for the presence of a murmur. The potential cause of a cardiac murmur ranges from completely innocent to advanced valvular disease, each with its own distinctive characteristics. For study purposes, we will score this item as: present or absent.
- *Crepitations:* we will auscultate the lungs for crepitations, or crackles. In general a distinction between in- or expiratory and fine or coarse crepitations is made (32). Crepitations can be a symptom of several diseases, ranging from pneumonia and pulmonary edema to interstitial lung fibrosis (32). For study purposes, we will only make a distinction in: present or absent.

2. *Rhonchi (per September 1st, 2015):* we will also auscultate the lungs for rhonchi or wheezes. A distinction between in- and/or expiratory rhonchi as well as high or low-frequent rhonchi can be made (32). Rhonchi or wheezes can be a symptom of asthma, chronic obstructive pulmonary disease or pulmonary secretions. For study purposes, we will score this item as: present or absent.

- *Mechanical ventilation:* data on the presence and type of mechanical ventilation will be gathered, as well as basic information on respiratory conditions (i.e. PEEP and respiratory rate). Note: in case of mechanical ventilation the value 'respiratory rate' will be filled in twice in the CRF. If both values are the same, it will be assumed that the patient breathes at a machine-set respiratory rate. If they differ, spontaneous breathing will be assumed. Tidal volume and inspiratory oxygen (percentage) were added *per September 1st, 2015*.
- *Inotrope and vasopressor use:* any inotrope or vasopressor requirement, type, dose and speed will be recorded.

- *Estimations of pump function and peripheral circulation:* an estimation will be made, either by a member of the treating team, or by the researcher.

Longitudinal assessment of above hemodynamic variables (per August 12th, 2015):

7. To evaluate the association of conventional hemodynamic variables with the cardiac output on two different points: within the first 24 hours of admission and 24 hours thereafter.

Laboratory values closest to examination:

- *Blood gas analysis: arterial pH, carbon dioxide, oxygen, bicarbonate, base excess, lactate and hemoglobin:* these are frequently determined as part of regular ICU physiologic monitoring. For study purposes, we will use the value closest to our examination. Arterial hemoglobin and lactate will serve as markers for macrocirculatory and microcirculatory status, respectively. Arterial oxygen levels will be used to determine the PaO2/FiO2 ratio closest to our examination.
- Serum leucocytes, hematocrit, thrombocytes, high-sensitive troponin-T, aspartate transaminase, alanine transaminase, total and direct bilirubin: these are determined as part of regular care. For study purposes, we will use the value closest to our examination. These lab values are added for determination of sequential organ failure assessment (SOFA)-scores, blood viscosity, cardiomyocyte damaging and congestion of liver flow.

8. *Serum creatinine, urea and albumin:* these are determined as part of regular care. For study purposes, we will use values at admission, closest to our examination and the determined values until 72 hours after examination.
8. *24-hour urine creatinine, urea and albumin:* these are determined as part of regular care. For study purposes. We will use values at admission, closest to our examination and the determined values until 72 hours after examination.

After the physical examination has been performed, information on the following general characteristics will be extracted from patient files: demographic data, diagnoses and severity of illness as evaluated by the APACHE II and IV scores, Simplified Acute Physiology Score II (SAPS) and the Sequential Organ Failure Assessment (SOFA) (33-35). Furthermore we will collect EMV scores, lab values (details are described above), urine output (details are described above), ~~routine admission ECG's and routine~~ and evaluate admission thorax photo's on the presence of consolidations, overfilling and pleural effusions. We will also score known risk factors of ARDS according to the Berlin

criteria (36). After 90 days we will assess the patient files again to gather information on total ICU stay in days and 7-, 30- and 90-day mortality.

19 Data management

Data will be recorded using OpenClinica and transferred for analysis. After transfer from OpenClinica, all data will be managed in a database created using Stata version 14.2 (StataCorp, College Station, TX).

All study subjects will receive a study subject ID, compiled of the study name and their inclusion number (table 1) This study subject ID will be used in both OpenClinica and Stata. Only a researcher with 'study director' account properties in OpenClinica will be able to link study subject ID to patient number.

Study name	Inclusion number	Study subject ID
SICS1	0001	SICS1_0001
SICS1	0002	SICS1_0002

Table 2. Compiling study subject ID's

~~Images will be saved anonymously and will be coded in a systematic fashion, using the study subject ID, session number, and image contents. An example is in table 2.~~

~~Study subject number~~	~~Session number~~	~~Image contents~~	~~Image name~~
~~SICS1_0001~~	~~01~~	~~LVOT~~	~~SICS1_0001_01_LVOT~~
~~SICS1_0001~~	~~01~~	~~ECG~~	~~SICS1_0001_01_ECG~~

~~**Table 2.** Naming of images~~

For a complete overview of naming images see appendix 4, 5 and 6. A standardized way of inclusion and adding subjects to the database is provided in appendix 6 (Dutch).

20 Statistical methods

a) Outcomes

We will use the general characteristics to create a baseline table. Statistical analyses will be performed using Stata version 14.2 (StataCorp, College Station, TX). Data will be presented as means with standard deviation if normally distributed or as medians with ranges in case of skewed data.

b) Additional analyses

Univariate analyses will be conducted and all variables with p<0.1 will be included in the multivariate models. Multivariate analyses will be conducted using a stepwise model. Cardiac output will be modeled using linear regression and mortality will be modeled using logistic regression. All analyses will be adjusted for age and gender; other general characteristics will not be added to the model standardly. All analyses will be tested two-sided and p-values of less than 0.05 will be considered statistical significant.

c) Analysis population and missing data

Primary analyses will be performed with imputation for missing data using multiple imputations. Robustness of conclusions will be checked by secondary sensitivity analyses only including available data.

d) Subgroup analyses

If sample size permits, we will conduct an analysis in different subpopulations. Examples of subpopulations that may be eligible for further analysis are those with different types of shock (distributive, obstructive, hypovolemic, cardiogenic), CVVH, heart failure by any cause, myocardial infarction, atrial fibrillation or surgery versus no-surgery patient groups.

[21] Appendices

Appendix 1A: Overview of the add-ons and their starting date

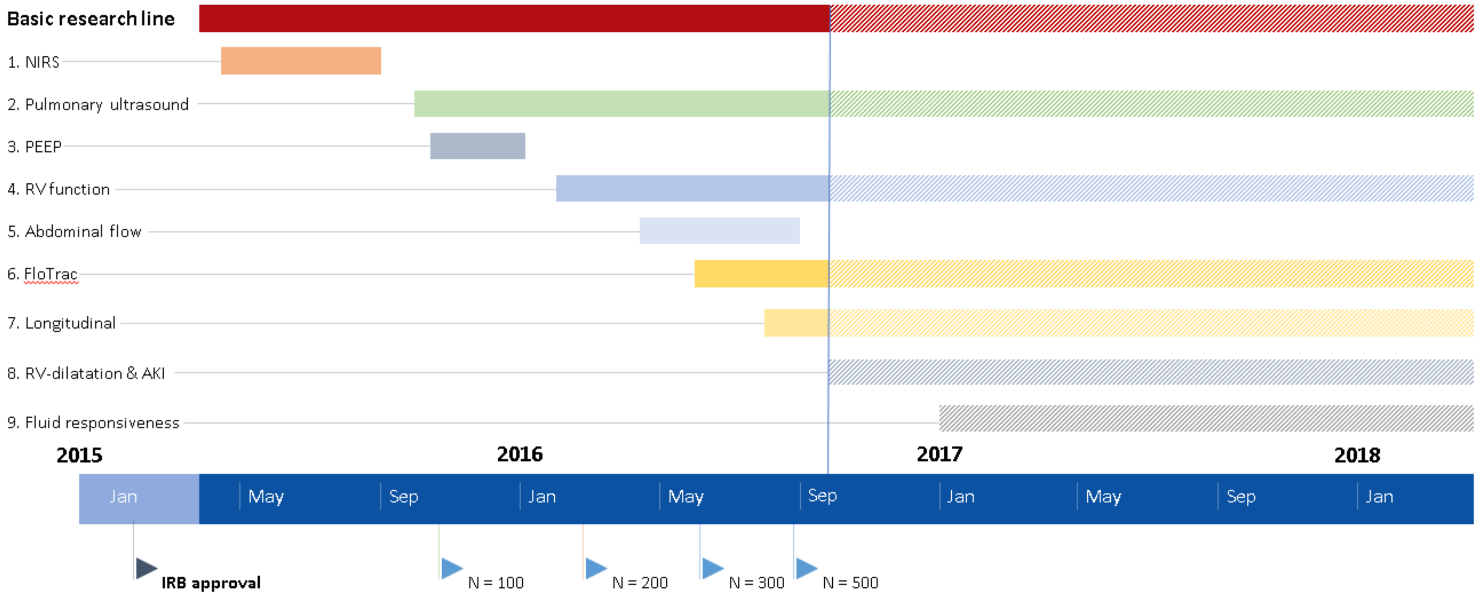

Figure 1. Overview of add-ons and their starting dates

Appendix 1B: Table of add-ons and their starting date

Add-on title	Dates	SICS nr	Notes
NIRS	01-04-'15 to 05-08-'15	5 to 85	N = 35
Pulmonary ultrasound	09-09-'15 to date	86 to date	
PEEP challenge	01-09-'15 to 03-01-'15	86 to 151	N = 8
RV-function	10-02-'16 to date	194 to date	
Abdominal flow	14-04-'16 to 17-08-'16	278 to 501	N = 59
FloTrac	01-06-'16 to date	378 to date	N = 13
Longitudinal	12-08-'16 to date	493 to date	N = 28
RV-dilatation & AKI	25-10-'16 to date	600 to date	
Fluid responsiveness	01-01-'17 (planned)	To follow	

Abbreviations: NIRS = near infrared spectroscopy; PEEP = peak end-expiratory pressure; RV = right ventricle; AKI = acute kidney injury.
Note: not all patients could be included in the add-on studies. Add-on 1: unavailability of the InSpectra (n = 29), cirrhosis (n = 8), no consent (n = 5) or a total hemoglobin index below 5 (n = 3). Add-on 3: no mechanical ventilation (n = 37), no intensivist available for supervision (n = 12), contraindication for challenge (n= 8). Add-on 5: flow measurements too time consuming (n = 153), inadequate flow measurements (n = 7), inadequate cardiac output measurements (n = 3), refusal of consent (n = 1). Add-on 6 and 7: only includes a subpopulation of patients in circulatory shock.

Appendix 2: Flow chart of data collection moments

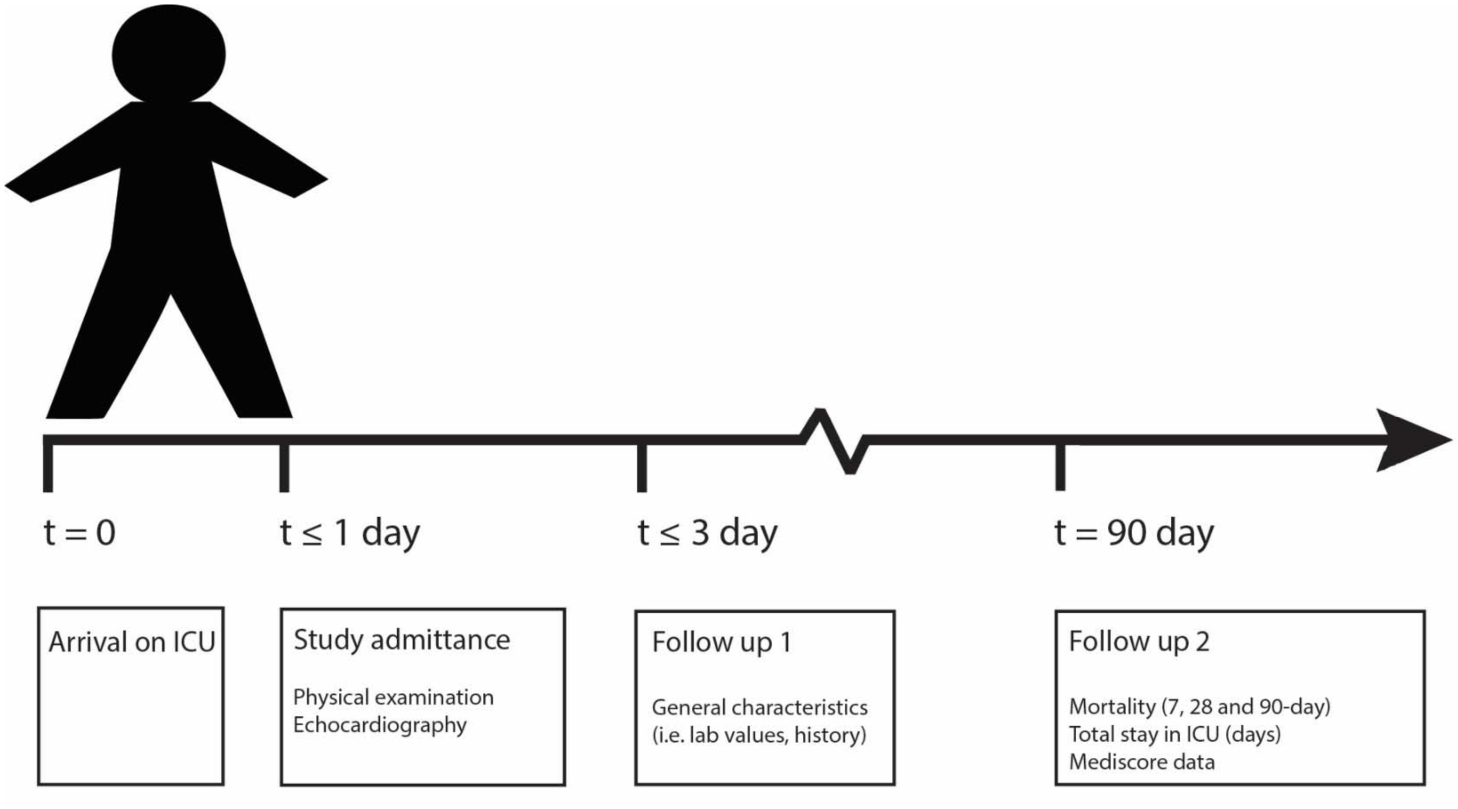

Figure 2. Flow chart of data collection moments

Appendix 3: Variables

Table 3. Overview of all variables. From left to right: the variables, their potential outcome, the method and timing of measurements, and how they will be recorded.

Variable	Value	How to measure	When to obtain	Database
Study subject ID	SICS_0001, etc.	Decoding file or poliplus	Admittance	OpenClinica
Examination date	-	-	Admittance	OpenClinica
Examination time	-	-	Admittance	OpenClinica
Gender	M/F	Poliplus	Admittance	OpenClinica
Age	-	Poliplus	Admittance	OpenClinica
Height	0 - 240	Patient file	Admittance	OpenClinica
Weight	0 - 300	Patient file	Admittance	OpenClinica
Respiratory rate	0 - 60	Observation	Admittance	OpenClinica
Heart rate	0 - 300	ECG monitor (ECG leads)	Admittance	OpenClinica
Heart rhythm	Reg/irr	ECG monitor (ECG leads)	Admittance	OpenClinica
Atrial fibrillation	Y/N	ECG monitor (ECG leads)	Admittance	OpenClinica
Systolic blood pressure 1	0 - 350	ECG monitor (artery line)	Admittance	OpenClinica
Diastolic blood pressure 1	0 - 200	ECG monitor (artery line)	Admittance	OpenClinica
Mean arterial pressure 1	0 - 150	ECG monitor (artery line)	Admittance	OpenClinica
Blood pressure 1 arm	Left/right	Observation	Admittance	OpenClinica
Central venous pressure	0 - 40	ECG monitor (central venous line)	Admittance	OpenClinica
Urine output one hour	0 - 1500	Poliplus / urine catheter	Admittance	OpenClinica
Urine output total	0 - 6000	Poliplus / urine catheter	Admittance	OpenClinica
Urine output ‘x’ hours	0 - 6	Poliplus / urine catheter	Admittance	
Inotropics type	-	Bedside pump	Admittance	OpenClinica
Inotropics dose	5-in-50, 10-in-50, 20-in-50, 50-in-50, 100-in-50, 200-in-50, 250-in-50, 500-in-50, 40-in-40	Bedside pump	Admittance	OpenClinica
Inotropics speed	0 - 50	Bedside pump	Admittance	OpenClinica
Mechanical ventilation	Y/N	Ventilation machine	Admittance	OpenClinica
PEEP	0 - 30	Ventilation machine	Admittance	OpenClinica
Respiratory rate machine (set)	0 - 40	Ventilation machine	Admittance	OpenClinica
Tidal volume	0 - 1000	Ventilation machine	Admittance	OpenClinica

Variable	Value	How to measure	When to obtain	Database
Inspiratory O_2 percentage	0 - 100	Ventilation machine	Admittance	OpenClinica
AVPU score	0 - 3	Observation	Admittance	OpenClinica
Systolic blood pressure 2	0 - 350	Sphygmomanometer (cuff)	Admittance	OpenClinica
Diastolic blood pressure 2	0 - 200	Sphygmomanometer (cuff)	Admittance	OpenClinica
Mean arterial pressure 2	0 - 150	Sphygmomanometer (cuff)	Admittance	OpenClinica
Blood pressure 2 arm	Left/right	Observation	Admittance	OpenClinica
Souffles	Y/N	Auscultation	Admittance	OpenClinica
Rales/crepitations	Y/N	Auscultation	Admittance	OpenClinica
Rhonchi	Y/N	Auscultation	Admittance	OpenClinica
Mottling score	0-5	Observation (see figure 2)	Admittance	OpenClinica
Capillary refill index sternum	0 - 25	Palpation	Admittance	OpenClinica
Capillary refill index finger	0 - 25	Palpation	Admittance	OpenClinica
Capillary refill knee	0 - 25	Palpation	Admittance	OpenClinica
Temperature subjective	Warm/cold	Palpation	Admittance	OpenClinica
Temperature forearm	0 - 45	Skin probes	Admittance	OpenClinica
~~Temperature index finger~~	~~0 - 45~~	~~Skin probes~~	~~Admittance~~	~~OpenClinica~~
Tskin-diff	0 - 45	Calculation	Admittance	OpenClinica
Temperature central	1 - 45	Bladder temp probes	Admittance	OpenClinica
Temperature peripheral big toe	2 - 45	Skin probes	Admittance	OpenClinica
Temperature peripheral dorsum of foot	2 - 45	Skin probes	Admittance	OpenClinica
dTc-p	3 - 45	Calculation	Admittance	OpenClinica
Peripheral circulation *estimation*	Poor/good perfusion	Question	Admittance	OpenClinica
Pump function *estimation*	Poor, moderate, reasonable, good	Question	Admittance	OpenClinica
Degree of training	Nurse to intensivist	Question	Admittance	OpenClinica
LVOT diameter	0 – 10	Echocardiography	Admittance	OpenClinica
Peak flow velocity	0 – 10	Echocardiography	Admittance	OpenClinica
Velocity time integral	0 – 100	Echocardiography	Admittance	OpenClinica
Heart rate (echo)	0 – 300	Echocardiography	Admittance	OpenClinica
Cardiac output	0 - 20	Echocardiography	Admittance	OpenClinica
Cardiac index	0 - 20	Echocardiography	Admittance	OpenClinica
Stroke volume	0 – 200	Echocardiography	Admittance	OpenClinica

Variable	Value	How to measure	When to obtain	Database
~~PLAX~~	~~SICS1_0001_01_ PLAX~~	~~Echocardiography~~	~~Admittance~~	~~OpenClinica~~
~~PLAX – LVOT~~	~~SICS1_0001_01_ LVOT~~	~~Echocardiography~~	~~Admittance~~	~~OpenClinica~~
~~AP5CH~~	~~SICS1_0001_01_ AP5CH~~	~~Echocardiography~~	~~Admittance~~	~~OpenClinica~~
~~AP5CH – VTI~~	~~SICS1_0001_01_ VTI~~	~~Echocardiography~~	~~Admittance~~	~~OpenClinica~~
StO_2 thenar	0 - 100	InSpectra™	Admittance	OpenClinica
StO_2 knee	0 - 100	InSpectra™	Admittance	OpenClinica
B-lines superior left	0 - 5	Pulmonary ultrasound	Admittance	OpenClinica
B-lines superior right	0 - 5	Pulmonary ultrasound	Admittance	OpenClinica
B-lines inferior left	0 - 5	Pulmonary ultrasound	Admittance	OpenClinica
B-lines inferior right	0 - 5	Pulmonary ultrasound	Admittance	OpenClinica
B-lines lateral lift	0 - 5	Pulmonary ultrasound	Admittance	OpenClinica
B-lines lateral right	0 - 5	Pulmonary ultrasound	Admittance	OpenClinica
PEEP - heart rate	0 - 300	Echocardiography	Admittance	OpenClinica
PEEP - systolic blood pressure	0 - 350	Echocardiography	Admittance	OpenClinica
PEEP - diastolic blood pressure	0 - 200	Echocardiography	Admittance	OpenClinica
PEEP – mean arterial pressure	0 - 150	Echocardiography	Admittance	OpenClinica
PEEP – Blood pressure arm	Left/right	Echocardiography	Admittance	OpenClinica
PEEP – Central venous pressure	0 - 40	Echocardiography	Admittance	OpenClinica
PEEP – Cardiac output	0 - 20	Echocardiography	Admittance	OpenClinica
TAPSE	0 - 50	Echocardiography	Admittance	OpenClinica
RV S’	0 - 40	Echocardiography	Admittance	OpenClinica
CCA flow left	0 - 3000	Vascular ultrasound	Admittance	OpenClinica
CCA flow right	0 - 3000	Vascular ultrasound	Admittance	OpenClinica
CCA TAmean left	0 - 100	Vascular ultrasound	Admittance	OpenClinica
CCA TAmean right	0 - 100	Vascular ultrasound	Admittance	OpenClinica
CCA diameter left	0 - 1	Vascular ultrasound	Admittance	OpenClinica
CCA diameter right	0 - 1	Vascular ultrasound	Admittance	OpenClinica
CCA flow left	0 - 2000	Vascular ultrasound	Admittance	OpenClinica
CCA flow right	0 - 2000	Vascular ultrasound	Admittance	OpenClinica
SCA TAmean left	0 - 100	Vascular ultrasound	Admittance	OpenClinica
SCA TAmean right	0 - 100	Vascular ultrasound	Admittance	OpenClinica

Variable	Value	How to measure	When to obtain	Database
SCA diameter left	0 - 1	Vascular ultrasound	Admittance	OpenClinica
SCA diameter right	0 - 1	Vascular ultrasound	Admittance	OpenClinica
SCA flow left	0 - 2000	Vascular ultrasound	Admittance	OpenClinica
SCA flow right	0 - 2000	Vascular ultrasound	Admittance	OpenClinica
CFA TAmean left	0 - 100	Vascular ultrasound	Admittance	OpenClinica
CFA TAmean right	0 - 100	Vascular ultrasound	Admittance	OpenClinica
CFA diameter left	0 - 1	Vascular ultrasound	Admittance	OpenClinica
CFA diameter right	0 - 1	Vascular ultrasound	Admittance	OpenClinica
CFA flow left	0 - 2000	Vascular ultrasound	Admittance	OpenClinica
CFA flow right	0 - 2000	Vascular ultrasound	Admittance	OpenClinica
RV basal diameter	0 – 99	Echocardiography	Admittance	OpenClinica
RV mid cavity diameter	0 – 99	Echocardiography	Admittance	OpenClinica
RV longitudinal diameter	0 - 99	Echocardiography	Admittance	OpenClinica
Tricuspid regurgitation peak velocity	0 - 30	Echocardiography	Admittance	OpenClinica
Examination date	-	-	Add-on: 1-day	OpenClinica
Examination time	-	-	Add-on: 1-day	OpenClinica
Height	0 - 240	Patient file	Add-on: 1-day	OpenClinica
Weight	0 - 300	Patient file	Add-on: 1-day	OpenClinica
Respiratory rate	0 - 60	Observation	Add-on: 1-day	OpenClinica
Heart rate	0 - 300	ECG monitor (ECG leads)	Add-on: 1-day	OpenClinica
Heart rhythm	Reg/irr	ECG monitor (ECG leads)	Add-on: 1-day	OpenClinica
Atrial fibrillation	Y/N	ECG monitor (ECG leads)	Add-on: 1-day	OpenClinica
Systolic blood pressure 1	0 - 350	ECG monitor (artery line)	Add-on: 1-day	OpenClinica
Diastolic blood pressure 1	0 - 200	ECG monitor (artery line)	Add-on: 1-day	OpenClinica
MAP 1	0 - 150	ECG monitor (artery line)	Add-on: 1-day	OpenClinica
Blood pressure 1 arm	Left/right	Observation	Add-on: 1-day	OpenClinica
CVP	0 - 40	ECG monitor (central venous line)	Add-on: 1-day	OpenClinica
Urine output one hour	0 - 1500	Poliplus / urine catheter	Add-on: 1-day	OpenClinica
Urine output total	0 - 6000	Poliplus / urine catheter	Add-on: 1-day	OpenClinica
Urine output 'x' hours	0 – 6	Poliplus / urine catheter	Add-on: 1-day	
Inotropics type	-	Bedside pump	Add-on: 1-day	OpenClinica
Inotropics dose	5-in-50, 10-in-50, 20-in-50, 50-in-	Bedside pump	Add-on: 1-day	OpenClinica

Variable	Value	How to measure	When to obtain	Database
	50, 100-in-50, 200-in-50, 250-in-50, 500-in-50, 40-in-40			
Inotropics speed	0 - 50	Bedside pump	Add-on: 1-day	OpenClinica
Mechanical ventilation	Y/N	Ventilation machine	Add-on: 1-day	OpenClinica
PEEP	0 - 30	Ventilation machine	Add-on: 1-day	OpenClinica
Respiratory rate machine (set)	0 - 40	Ventilation machine	Add-on: 1-day	OpenClinica
AVPU score	0 - 3	Observation	Add-on: 1-day	OpenClinica
Souffles	Y/N	Auscultation	Add-on: 1-day	OpenClinica
Rales/crepitations	Y/N	Auscultation	Add-on: 1-day	OpenClinica
Mottling score	0-5	Observation (see figure 2)	Add-on: 1-day	OpenClinica
Capillary refill index finger	0 - 25	Palpation	Add-on: 1-day	OpenClinica
Capillary refill knee	0 - 25	Palpation	Add-on: 1-day	OpenClinica
Temperature subjective	Warm/cold	Palpation	Add-on: 1-day	OpenClinica
Temperature index finger	0 - 45	Skin probes	Add-on: 1-day	OpenClinica
Tskin-diff	0 - 45	Calculation	Add-on: 1-day	OpenClinica
Temperature central	1 - 45	Skin probes	Add-on: 1-day	OpenClinica
Temperature peripheral	2 - 45	Skin probes	Add-on: 1-day	OpenClinica
dTc-p	3 - 45	Calculation	Add-on: 1-day	OpenClinica
Peripheral circulation *estimation*	Poor/good perfusion	Question	Add-on: 1-day	OpenClinica
Pump function *estimation*	Poor, moderate, reasonable, good	Question	Add-on: 1-day	OpenClinica
Degree of training	Nurse to intensivist	Question	Add-on: 1-day	OpenClinica
LVOT diameter	0 - 10	Echocardiography	Add-on: 1-day	OpenClinica
Peak flow velocity	0 - 10	Echocardiography	Add-on: 1-day	OpenClinica
Velocity time integral	0 - 100	Echocardiography	Add-on: 1-day	OpenClinica
Heart rate (echo)	0 - 300	Echocardiography	Add-on: 1-day	OpenClinica
Cardiac output	0 - 20	Echocardiography	Add-on: 1-day	OpenClinica
Cardiac index	0 - 20	Echocardiography	Add-on: 1-day	OpenClinica
Stroke volume	0 - 200	Echocardiography	Add-on: 1-day	OpenClinica
Tricuspid annular plane systolic	0 - 50	Echocardiography	Add-on: 1-day	OpenClinica

Variable	Value	How to measure	When to obtain	Database
excursion				
systolic excursion velocity	0 - 40	Echocardiography	Add-on: 1-day	OpenClinica
FloTrac inclusion	Y/N	Inclusion criteria met?	Add-on: FloTrac	OpenClinica
FloTrac date	-	-	Add-on: FloTrac	OpenClinica
FloTrac time	-	-	Add-on: FloTrac	OpenClinica
FloTrac – velocity time integral (echo)	0 - 40	Echocardiography	Add-on: FloTrac	OpenClinica
FloTrac – heart rate (echo)	0 - 200	Echocardiography	Add-on: FloTrac	OpenClinica
FloTrac – cardiac output (echo)	0 - 20	Echocardiography	Add-on: FloTrac	OpenClinica
FloTrac – quality (echo)	Poor, suboptimal, optimal	Echocardiography	Add-on: FloTrac	OpenClinica
FloTrac – cardiac output	0 - 20	EV1000® monitor	Add-on: FloTrac	OpenClinica
FloTrac – noradrenalin dose	0 - 1	Bedside pump	Add-on: FloTrac	OpenClinica
FloTrac – inotropic dose	0 - 1	Bedside pump	Add-on: FloTrac	OpenClinica
FloTrac – inotropic type	-	Bedside pump	Add-on: FloTrac	OpenClinica
FloTrac – mottling score	0 - 5	Observation (see figure 2)	Add-on: FloTrac	OpenClinica
ICU admission date	-	Poliplus	3-days: clinical	OpenClinica
ICU admission time	-	Poliplus	3-days: clinical	OpenClinica
EMV score	0 - 15	Poliplus	3-days: clinical	OpenClinica
Admission reason	-	Poliplus	3-days: clinical	OpenClinica
Patient specifics	Surgery, CVVH, MI, congestive heart failure	Poliplus	3-days: clinical	OpenClinica
Shock type	Distributive, obstructive, cardiogenic, hypovolemic, none	Poliplus	3-days: clinical	OpenClinica
Final diagnosis	-	Poliplus	3-days: clinical	OpenClinica
~~X-Ray~~	~~SICS1_0001_01_Xray~~	~~Poliplus~~	~~3-days~~	~~OpenClinica~~
~~ECG~~	~~SICS1_0001_01_ECG~~	~~Poliplus~~	~~3-days~~	~~OpenClinica~~
Respiratory distress	-	Poliplus	3-days: ARDS	OpenClinica
Direct ARDS risk factors	-	Poliplus	3-days: ARDS	OpenClinica

Variable	Value	How to measure	When to obtain	Database
Indirect ARDS risk factors	-	Poliplus	3-days: ARDS	OpenClinica
Arterial pH	6.75 - 7.75	Poliplus	3-days: biochemical	OpenClinica
Arterial CO_2 pressure	1.9 - 90	Poliplus	3-days: biochemical	OpenClinica
Arterial O_2 pressure	1.9 – 30	Poliplus	3-days: biochemical	OpenClinica
Arterial bicarbonate (HCO_3^-)	5 - 50	Poliplus	3-days: biochemical	OpenClinica
Arterial base excess	-25 – 25	Poliplus	3-days: biochemical	OpenClinica
Arterial lactate	0 - 30	Poliplus	3-days: biochemical	OpenClinica
Arterial Hb	0 - 15	Poliplus	3-days: biochemical	OpenClinica
Serum leucocytes	0 - 100	Poliplus	3-days: biochemical	OpenClinica
Serum hematocrit	0.1 – 0.8	Poliplus	3-days: biochemical	OpenClinica
Serum thrombocytes	0 - 800	Poliplus	3-days: biochemical	OpenClinica
Serum NT-proBNP	-	Poliplus	3-days: biochemical	OpenClinica
Serum hs-troponin T	-	Poliplus	3-days: biochemical	OpenClinica
Serum ASAT	-	Poliplus	3-days: biochemical	OpenClinica
Serum ALAT	-	Poliplus	3-days: biochemical	OpenClinica
Serum total bilirubin	0 - 2000	Poliplus	3-days: biochemical	OpenClinica
Serum direct bilirubin	0 - 1500	Poliplus	3-days: biochemical	OpenClinica
Serum creatinine	0.1 - 9999	Poliplus	3-days: biochemical	OpenClinica
Serum urea	0.1 - 999	Poliplus	3-days: biochemical	OpenClinica
Serum albumin	0.1 - 999	Poliplus	3-days: biochemical	OpenClinica
Urine creatinine	0.1 - 9999	Poliplus	3-days: biochemical	OpenClinica
Urine urea	0.1 - 999	Poliplus	3-days: biochemical	OpenClinica
Urine albumin	0.1 - 999	Poliplus	3-days: biochemical	OpenClinica
Urine total volume	0 - 20000	Poliplus	3-days: biochemical	OpenClinica
Urine hours of collection	0 - 24	Poliplus	3-days: biochemical	OpenClinica
~~SOFA~~	~~0 – 24~~	~~Mediscore~~	~~90-days~~	~~OpenClinica~~
~~APACHE II~~	~~0 – 71~~	~~Mediscore~~	~~90-days~~	~~OpenClinica~~
~~APACHE IV~~	~~0 - 286~~	~~Mediscore~~	~~90-days~~	~~OpenClinica~~
Mortality 7 day	Y/N	Poliplus	90-days	OpenClinica
Mortality 30 day	Y/N	Poliplus	90-days	OpenClinica
Mortality 90 day	Y/N	Poliplus	90-days	OpenClinica
~~Total ICU stay~~	~~days~~	~~Poliplus~~	~~90-days~~	~~OpenClinica~~
~~Medical history~~	~~-~~	~~Mediscore~~	~~90-days~~	~~OpenClinica~~
~~Other lab values~~	~~-~~	~~Poliplus~~	~~90-days~~	~~OpenClinica~~
Any missing value	-9999	-	-	OpenClinica

Variable	Value	How to measure	When to obtain	Database
Any indeterminable value	-8888	-	-	OpenClinica

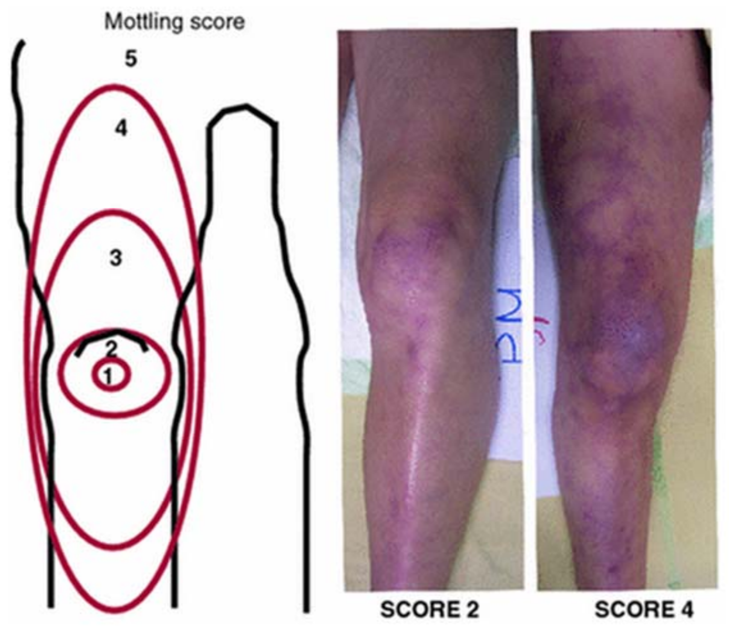

Figure 2. Mottling score. *Left:* the mottling score is based on a mottling area extension on the legs. Score 0 indicates no mottling ; score 1, a modest mottling area (coin size) localized to the center of the knee ; score 2, a moderate mottling area that does not exceed the superior edge of the kneecap ; score 3, a mild mottling area that does not exceed the middle thigh ; score 4, a severe mottling area that does not go beyond the fold of the groin ; score 5, an extremely severe mottling area that goes beyond the fold of the groin. *Right:* examples of the mottling score. Original illustration and text by Ait-Oufella et al (13)

Appendix 3: Echocardiography protocol

General outline

Cardiac output and cardiac index will be measured using transthoracic echocardiography. For study purposes different researchers will be trained in the basics of transthoracic echocardiography by a cardiologist. They will learn how to determine cardiac output by obtaining four different echocardiographic views and subsequent measurements.

Procedure

Transthoracic echocardiography will be performed at the bedside during the physical examination with a mobile ultrasonic machine ie General Electric Vivid-S6 with the use of the cardiac probe M3S of M4S with default cardiac imaging setting. The patient will be supine or in left lateral tilt (partly on the left). Physical assessment will be performed before examination. After the images have been acquired, cardiac output and cardiac index will be calculated and data will be saved on the hard disk. At a later time the images will be validated by an echocardiography technician or a cardiologist who will be blinded for all other measurements.

Views and images

Three or four standardized echographic views will be obtained in all patients:

1. Parasternal long axis view (PLAX);
2. parasternal short axis view (PSAX);
3. apical four chamber view (AP4CH);
4. apical five chamber view (AP5CH).

The PSAX view will only be obtained in case the PLAX does not provide a clear image of the aortic annulus. The views are described in more detail below.

Parasternal long axis (PLAX)

The parasternal window is located next to the sternum, between the 3rd and 5th intercostal space.

Criteria of quality for a good view (figure 3):

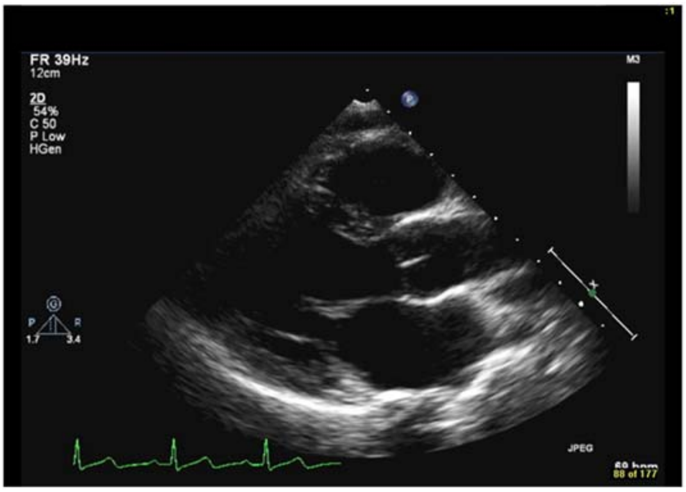

Figure 3. Parasternal long axis (PLAX)

- Minimized angle between ascending aorta and left ventricle;
- maximized width view of left ventricle;
- maximal opening of mitral valve (showing both anterior and posterior mitral valve leaflets, right- and noncoronary cusps of aortic valve;
- no papillary muscle in view.

The PLAX view is the primary view used to measure the left ventricular outflow tract (LVOT). An image will be saved for validation.

Parasternal short axis (PSAX)

This view will only be obtained in case the PLAX view does not provide a clear image of the LVOT. The PSAX view can be obtained on several levels. For study purposes it will be measured on the aortic, tricuspid and pulmonic valve level (figure 4). An image will be saved for validation.

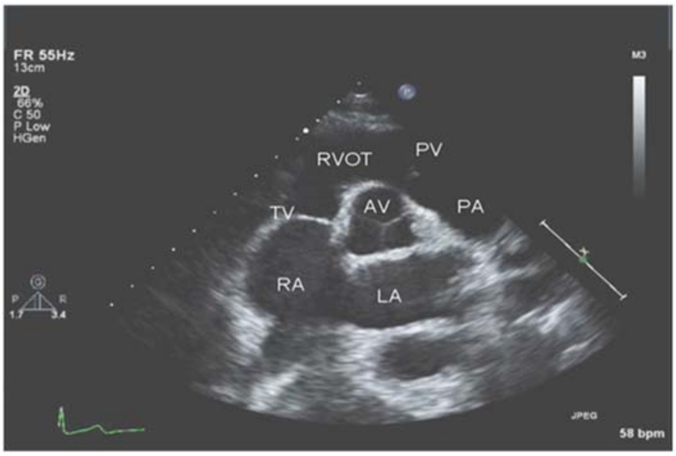

Figure 4. Parasternal short axis (PSAX)

Apical four and five chamber view (AP4CH and AP5CH)

The apical echographic window is located at the apex of the left ventricle (apical impulse).

Criteria of quality for a good view (figure 5, 6):

- Maximized view of endocardial border;
- Frames per second > 40;
- the entire endocardium is within scan sector in *both end-diastole and end-systole*;
- avoid apical foreshortening.

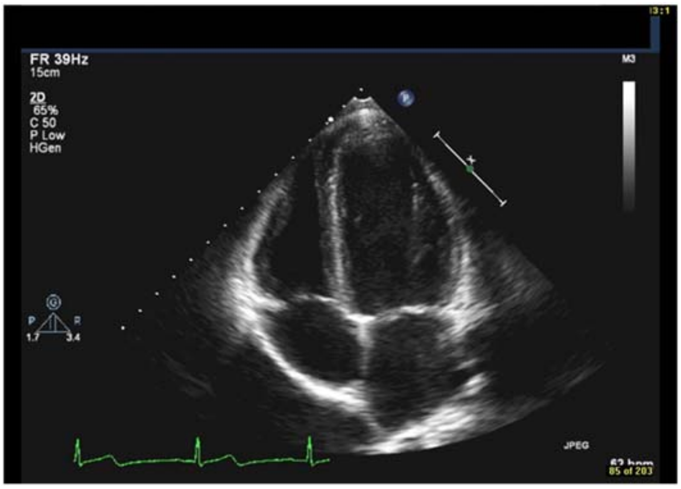

Figure 5. Apical four chamber view (AP4CH)

From the four chamber view the probe will be tilted caudally to obtain the apical five chamber view. In the apical five chamber view the velocity

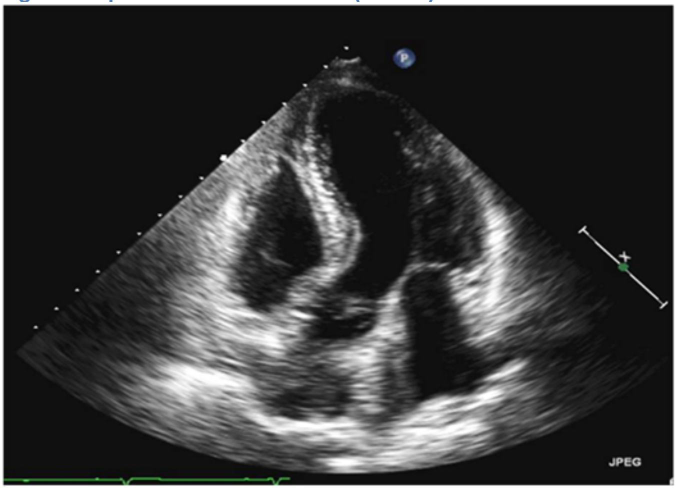

Figure 6. Apical five chamber view (AP5CH)

time integral will be measured using the pulse wave Doppler signal from the LVOT. Of both views an image will be saved for validation.

Measuring the left ventricular outflow tract (LVOT) and the velocity time integral (VTI)

The LVOT diameter changes very little through systole and diastole and is assumed to be constant and closely approximating a circle in shape. The LVOT diameter will be measured in 2D in the parasternal long axis view in systole (figure 7). If a clear image cannot be obtained through this view, the LVOT will be measured in the parasternal short axis or the AP5CH view.

The LVOT velocity time integral (LVOT-VTI) provides information regarding blood flow velocity across the time period of systole and is in the units of cm. Typical values are close to 2 cm. Blood flow velocity will be measured just above the aortic valve in the apical five chamber view by using pulse wave Doppler. The velocity time integral will be traced out on the ultrasound machine (figure 7). In case of an irregular rhythm such as atrial fibrillation, the average VTI of several beats will be used. Images of both measures will be saved for validation.

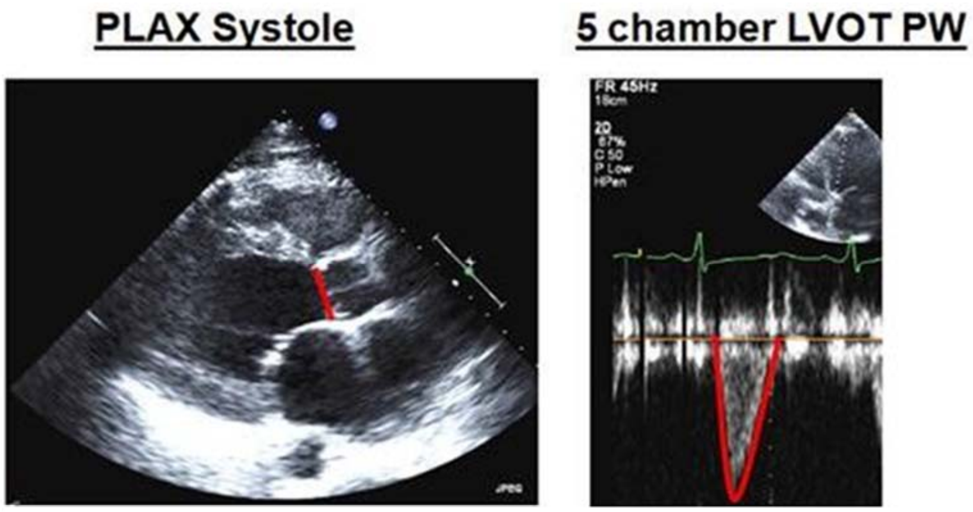

Figure 7. ***Left:*** **the left ventricular outflow tract (LVOT).** ***Right:*** **the velocity time integral (VTI).**

Calculating cardiac output and cardiac index

Cardiac output will be automatically calculated on the ultrasound machine after measuring the LVOT, VTI and heart rate. Cardiac index will be automatically derived using patient length and weight.

Measuring the tricuspid annular plane systolic excursion (TAPSE)

TAPSE is assessed in M-mode, after placing the cursor on the junction of the tricuspid valve and the RV free wall in the AP4CH view. The distance of tricuspid annular movement between end-diastole and end-systole in millimeters is measured.

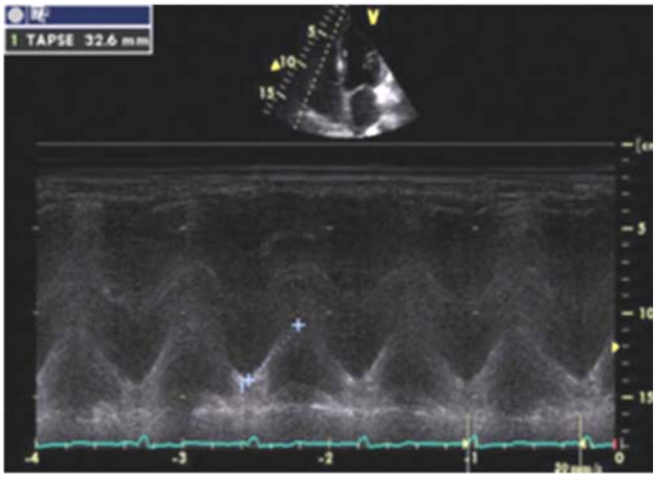

Figure 8. Assessment of TAPSE using the M-mode view

Measuring the systolic excursion velocity (RV S')

RV S` was also assessed in the AP4CH view, with a tissue velocity imaging mode highlighting the area of interest. The pulsed Doppler sample volume was placed at the tricuspid level of the RV free wall and the longitudinal velocity of excursion was measured. A image quality with at least 150 frames per second is required for reliable measurements. This can be obtained by narrowing the echocardiography window and dept of the probe. Optimal image orientation must be ensured in order to avoid underestimating velocities.

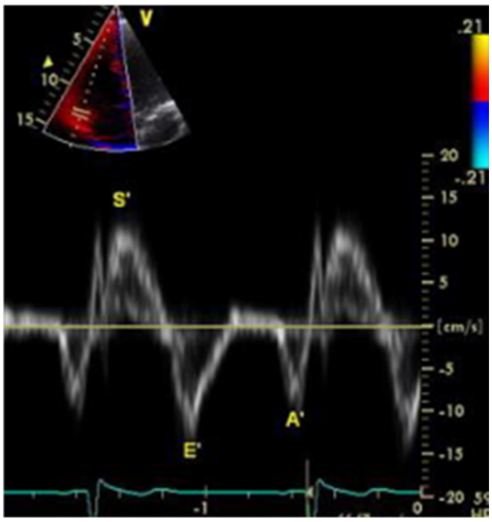

Figure 9. Assessment of the RV S' using the pulsed Doppler sample volume in the tissue velocity imaging mode

Data management

The echocardiographic images will be saved on the internal hard disk of the echo Doppler machine. This is required for later validation. The images can be used for patient management as soon as data collection or the obtained images have been supervised by a cardiologist. After the measurements the measurements will be entered in the data management system (OpenClinica). For validation, a USB drive or extern harddisk will be used to transfer images to the echolaboratory technician (corelab) for external validation and anonymization. The anonymized images and measurements will be stored on the central secure server of our department.

Appendix 4: NIRS-protocol

General outline

Peripheral tissue saturation (StO2) will be measured using a Near-infrared Spectroscopy (NIRS) device, the InSpectraTM tissue oxygenation monitor, model 650 (Hutchinson Technology, Inc., Hutchinson, MN). This is a non-invasive easy-to-use instrument. No additional training for researchers is necessary.

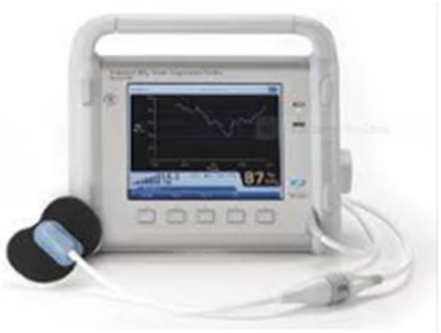

Figure 10. The InSpectraTM tissue oxygenation monitor

Procedure

NIRS will be measured during physical examination. No specific patient position is required. After a signal has been obtained, 1 minute of stabilization must precede a 30 second recording of the StO2. A steady signal should not fluctuate more than 5% during the measurement. If more deviations occur, the probe position and stability will be checked and a new measurement recorded.

The order of measurements will be:

1. Thenar (15 mm probe, left)
2. Knee (15 mm probe, right)

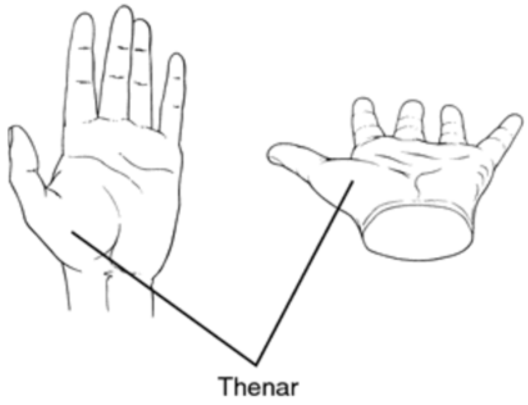

Figure 11A. The thenar region used for NIRS.

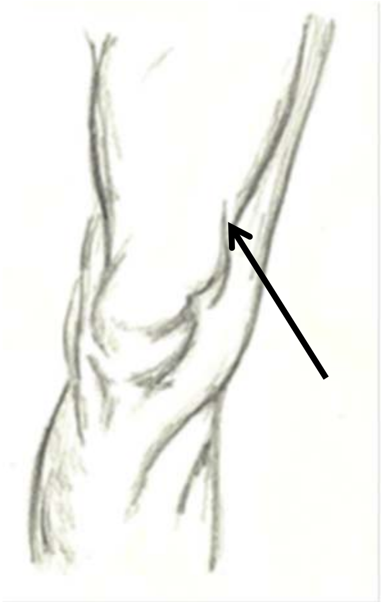

Figure 11B. Insertion of vastus medialis muscle (arrow) for NIRS

Appendix 4: Pulmonary ultrasound

General outline

The lungs will be imaged using pulmonary ultrasound. Similar to the transthoracic echocardiography training, researchers will receive a short session on how to obtain accurate pulmonary images. The will learn how to obtain pulmonary ultrasound images on six predefined points.

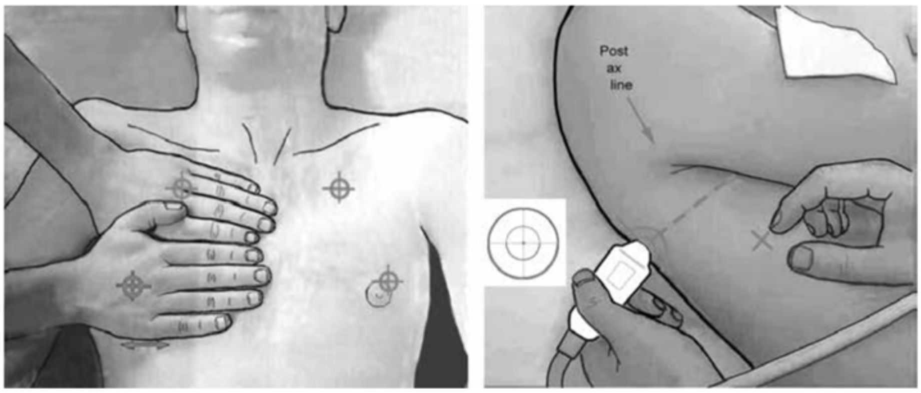

Figure 12. *Left:* the four BLUE-points. *Right:* the FALLS points.

Procedure

Upon finishing the transthoracic echocardiographic exam (appendix 2), pulmonary ultrasound will be performed subsequently with the same mobile ultrasonic machine (General Electric Vivid-S6) and cardiac probe (M3S of M4S). Default cardiac imaging settings were used combined with the maximal frequency setting (3.6 MHz). Patients will be investigated in the supine position. Images from each view are saved and stored on a secured UMCG server.

Views and images

Longitudinal scans were obtained from the intercostal space of each of the six BLUE points (figure 3) (25):

- Superior anterior midclavicular (bilateral)
- Inferior anterior midclavicular (bilateral)
- Lateral-inferior or mid-axial (bilateral)

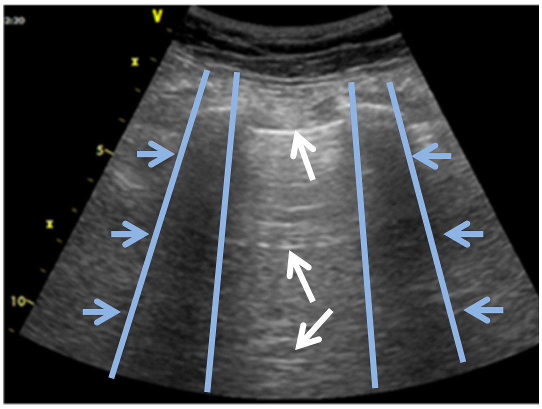

Figure 13. The bat sign (white arrows) and the horizontal A-lines (blue arrows)

The intercostal space was located when the shadows of the upper rib and lower rib enclosed the pleural line (called the 'bat sign'). After localization of the bat sign, we checked for horizontal lines (A lines) arising from the pleural line

repeating at regular intervals with a distance equal to that between the skin and the pleural line. A lines are indicative of normal lung surface. In addition, we sought vertical lines (B lines) obliterating the A lines. These lines arise from the pleural line, are hyperechoic, well defined and spread up to the lower edge of the screen. This phenomenon reflects the coexistence of elements with a major acoustic impedance gradient, i.e., fluid and air. The number of B lines in a single view was noted for each BLUE-point.

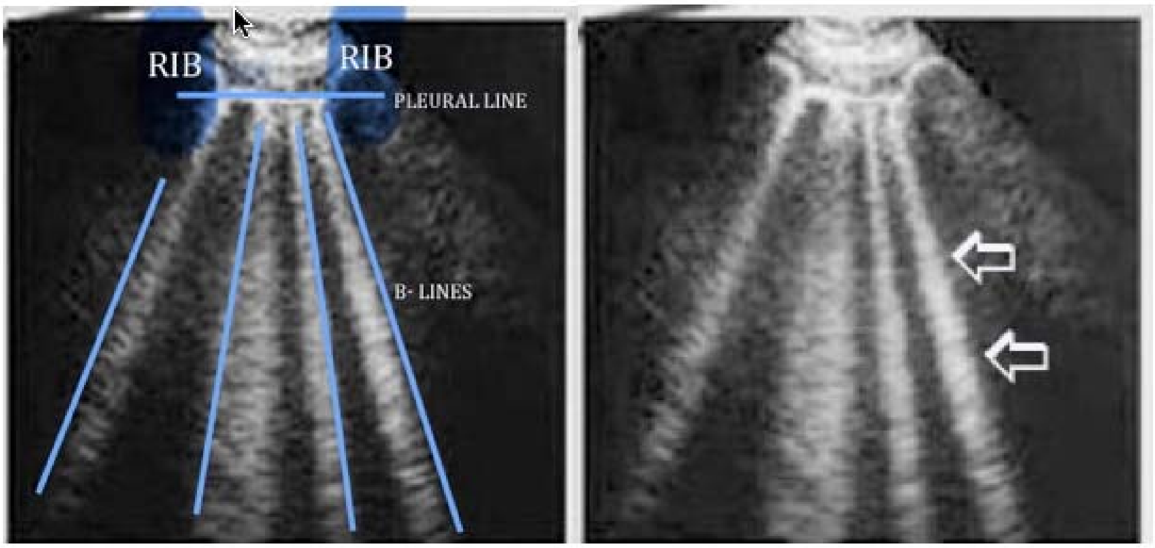

Figure 14. *Left:* B-lines indicated by blue stripes. *Right:* B-lines indicated by white arrows

Measuring B-lines and determining a B profile

Per view, the presence of B lines in three different interlobular septae is indicative of interstitial lung edema. This is objectified with ultrasound when three or more B lines with an intervening distance of at least 7 mm per scan are present, because thickened interlobular septae are typically 7 mm apart (25). When all the four anterior sites show three or more B lines this is called the B profile. This profile indicates diffuse alveolar interstitial edema. If three of the four sites show three or more B lines (technically three-quarters of a B-profile) this examination was also scored as B profile. Two-quarters of a B profile at the lower BLUE points are found in 5% cases of hemodynamic pulmonary edema and can therefore be considered as a B profile. For our purposes of using lung ultrasound for assessment of filling status we converted the number of B lines per site to ‘presence’ or ‘absence’ of B profile.

Appendix 5: Inclusion procedure (Dutch)

Screening op nieuwe patiënten

- ’s Ochtends worden de nieuwe opnames op de algemene ICV overdracht van 08:30 besproken;
- de research verpleegkundigen maken dagelijks een overzicht van de nieuw opgenomen patiënten van die ochtend;
- update het research overzicht zelf ’s middags met behulp van Poliplus data.

Inclusie

Acuut opgenomen patiënten, die zijn gescreend volgens bovenstaand schema en die voldoen aan de inclusiecriteria, worden binnen 24 uur na opname op de IC geïncludeerd. Het beste moment om te includeren is ‘s ochtends van **10:00 tot 12:00 uur** en ’s middags **vanaf 16:00 a 17:00 uur**. Voor 10:00 is de visite en wordt de patiënt gewassen door de verpleging. Van 12:00 tot 16:00 is bezoekuur. Na 16:00 is de patiënt vaak weer beschikbaar. Consent wordt verkregen van de patiënt zelf; als deze niet aanspreekbaar is, wordt consent in de eerste instantie van naasten verkregen en vervolgens van de patiënt.

Data invoer

Data wordt ingevoerd in OpenClinica, een online database die alleen vanaf de ICV server bereikbaar is. In de tutorial hieronder wordt beschreven hoe je een patiënt toevoegt en koppelt aan een meetmoment.

Database

Alle bestanden die worden geüpload in OpenClinica komen terecht in **dezelfde** map. Om deze reden moeten ze systematisch genummerd worden. Hieronder staat een kort overzicht van de naamgeving. Zie ook onder 17 datamanagement.

Onderdeel	Naam
Echo beelden	SICS1_0001_01_LVOT
	SICS1_0001_01_VTI
	etc
Informed consent	SICS1_0001_Consent family/patient

ECG	SICS1_0001_01_ECG
X-thorax	SICS1_0001_01_Xthorax

OpenClinica

1. **Inloggen**

   Gebruik je UMCG username en password.

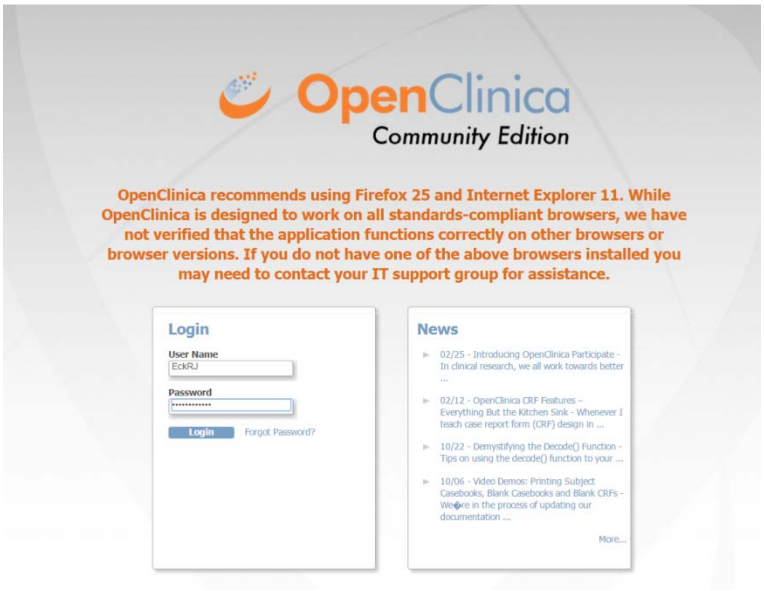

2. **Hoofdscherm**

   Hier zie je een overzicht van de ‘subject matrix’, oftewel je geïnludeerde patiënten.

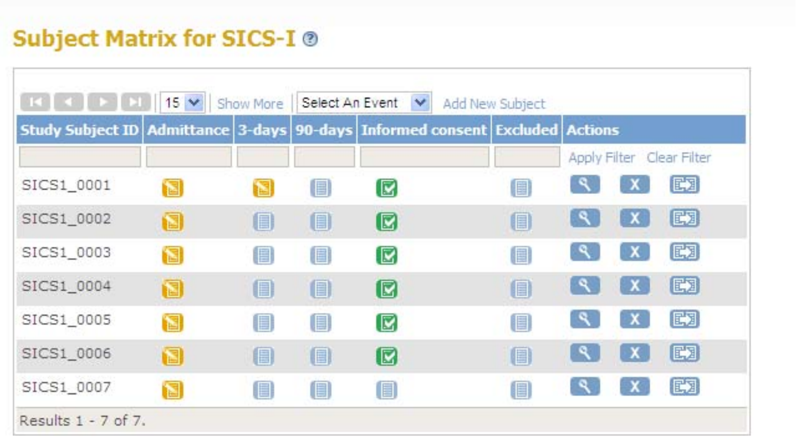

3. Patiënten toevoegen (1)

Klik op ‘tasks’ en vervolgens op ‘add subject’.

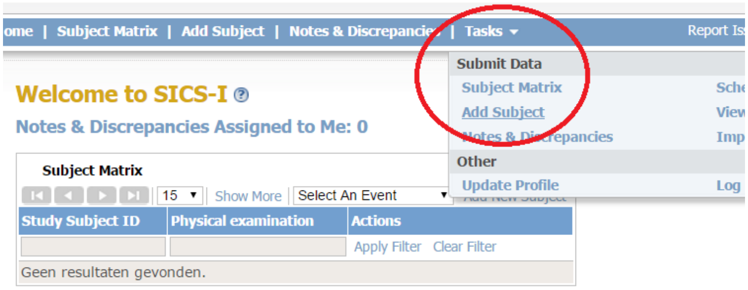

4. Patiënten toevoegen (2)

- *Study subject ID:* patiënt coderingsnummer (zie ook 17: Data management)
- *Person ID:* UMCG nummer
- *Secondary ID:* niet invullen
- Date of enrollment: huidige datum
- *Sex*: geslacht
- *Date of birth*: geboortedatum
- Als je alles hebt ingevuld: klik op ''save and assign study event''

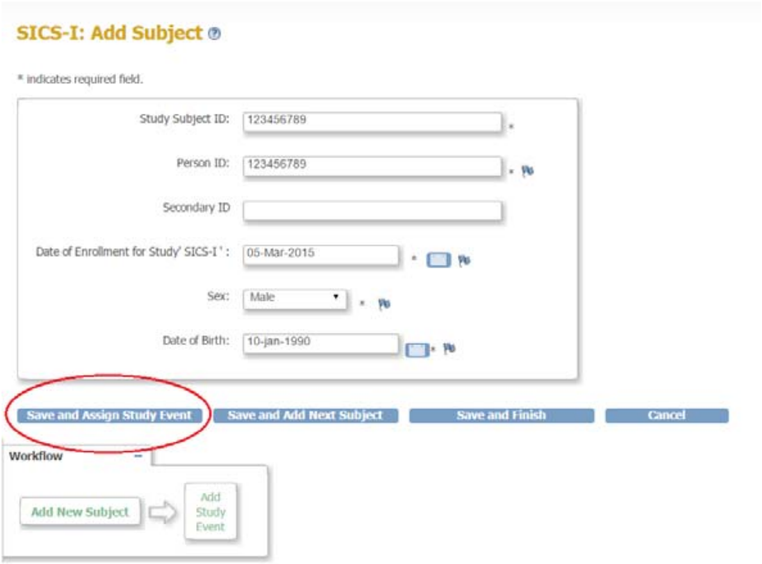

5. Een patiënt aan een 'Study Event' toevoegen

- OpenClinica noemt het moment binnen de studie waarop data wordt verzameld een 'Study Event'. De SICS-I studie heeft 5 evens: admittance, 3-days, 90-days, informed consent en excluded. Hieronder volgt meer uitleg over de momenten:

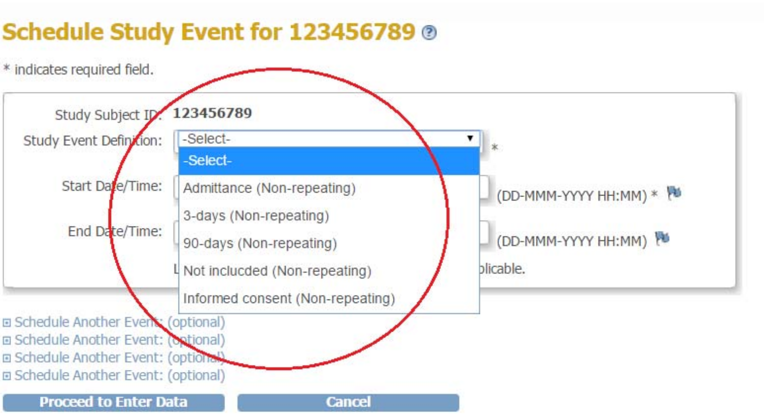

- Als een patiënt wordt <u>geïncludeerd</u>, dan wordt uiteindelijk ingevuld:

1. Admittance: hier worden data van lichamelijk onderzoek en echografie ingevoerd.
2. 3-days: hier worden (binnen 72 uur) patiëntdata uit Poliplus ingevoerd. Dit zijn bloedwaarden, ECG's, etc.
3. 90-days: hier worden (na 90 dagen) mortaliteit en Mediscore data ingevoerd.
4. Informed consent: hier wordt het informed consent formulier geüpload.

- Als een patiënt niet wordt geïncludeerd, dan wordt alleen ingevuld:

  1. Excluded: als een patient gescreend wordt en voldoet aan exclusiecriteria, of voldoet aan inclusiecriteria, maar niet wordt geïncludeerd (bijvoorbeeld om logistieke redenen).

1. ‘Start Date/Time’ en ‘End Date/Time’ kunnen leeg blijven.

6. Data invoeren (1)
   - Nadat je een event gekozen hebt, kan je hiervoor data invullen in het ‘Case Report Form (CRF)’.
   - Hiernaast is het event ‘Admittance’ gekozen. Het versienummer kan verschillen.
   - Onder ‘CRF name’ staat de bijbehorende CRF. Klik onder actions op de knop ‘enter data’ (kleine rode cirkel) om data in te voeren.

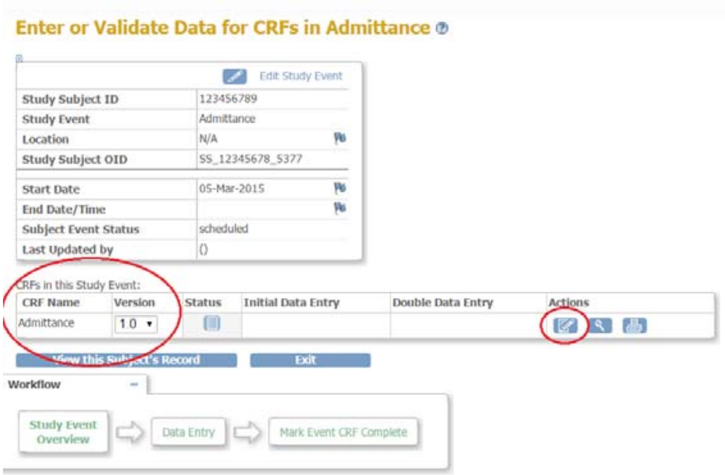

7. Data invoeren (2)

   Als je een CRF opent verschijnt het scherm hiernaast: je ziet de ‘CRF Header Info’ en de CRF zelf. Een korte toelichting:

   1. Study subject ID
   2. Vul hier jouw naam in
   3. Vul hier de huidige datum in
   4. Algemene invulinstructies; let hierop bij het invullen
   5. Als je de pagina hebt ingevuld, klik op ‘save’

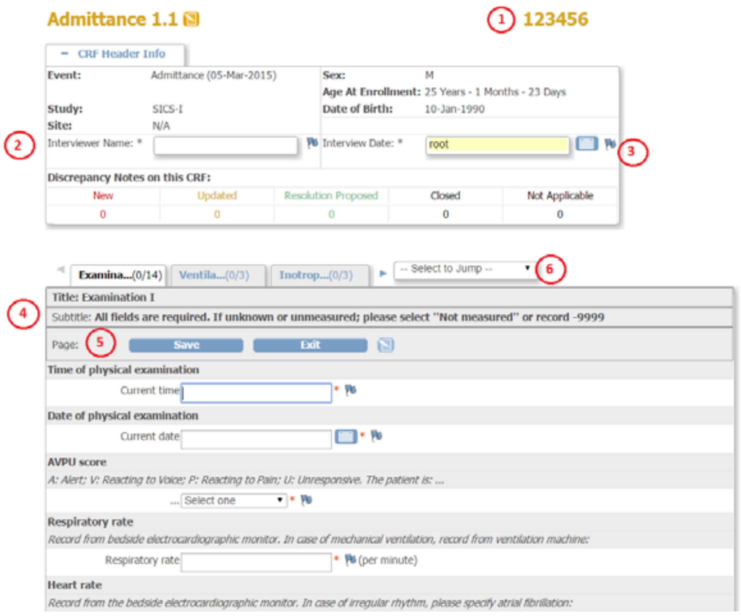

6. De CRF bestaat uit meerdere tabbladen (je ziet er hiernaast al 3). Klik hier om snel van het ene naar het andere tabblad te gaan.

8. Data invoeren (3)

Als *data niet verzameld werd*: vul dan '-9999' in. Klik op het vlaggetje om een verklaring te geven voor de missing value. Als er *geen data verzameld kon worden (patiënt heeft bijvoorbeeld steunkousen)*: vul dan '-8888' in. Geef wederom een verklaring.

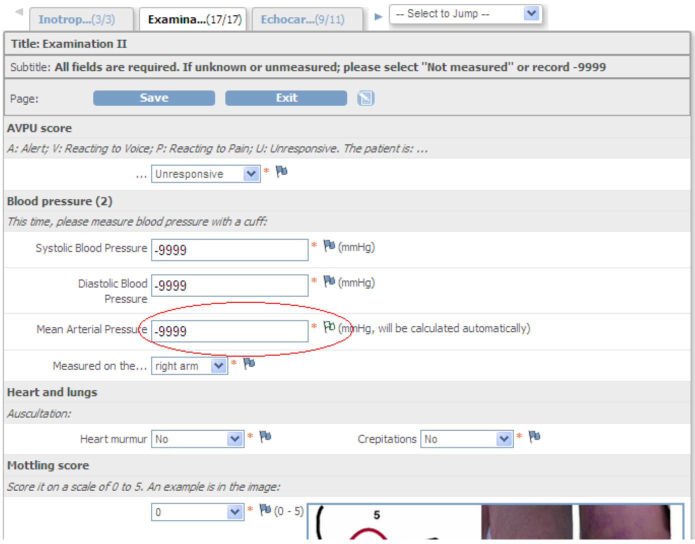

9. Data invoeren (4)

Als de data is ingevoerd en je niets meer hoeft toe te voegen, klik dan op 'Mark CRF Complete'. Vanaf dat moment kun je geen aanpassingen meer maken, **doe dit dus pas als de CRF echt compleet is ingevuld!**

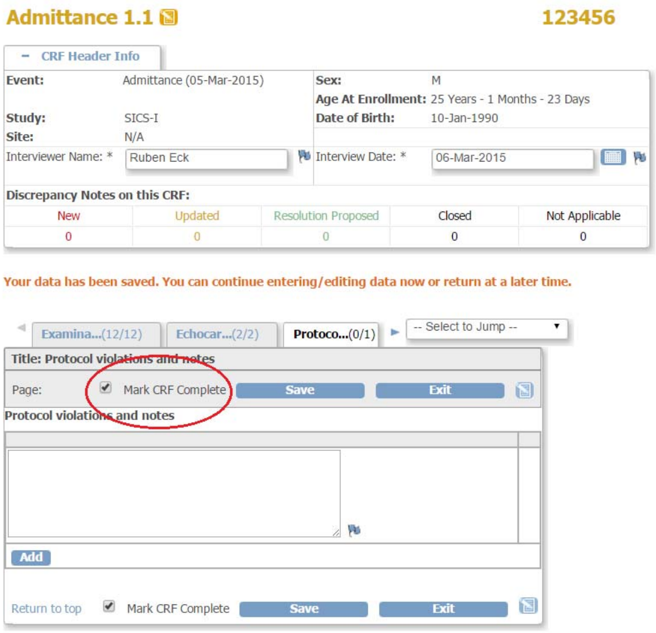

10. Nieuwe meetmomenten toevoegen

Om data voor een volgend meetmoment toe te voegen, ga je naar de subject matrix.

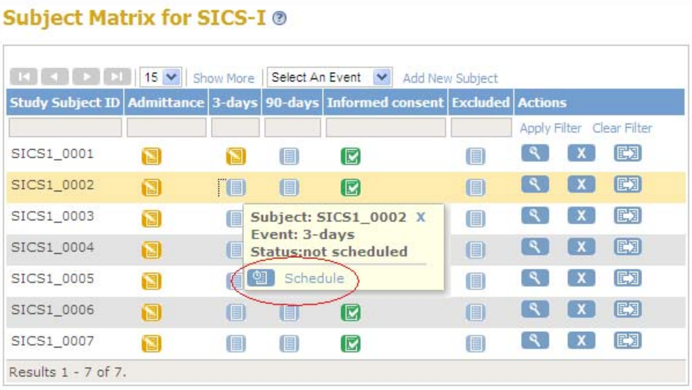

Klik op het icoontje van het meetmoment dat je wilt toevoegen. In het voorbeeld hiernaast wil je voor de patiënt met study subject ID ‘SICS1_0002’ data toevoegen in meetmoment ‘3-days’. Doe dit door te klikken op schedule en vervolgens het stappenplan vanaf 5 te herhalen.

[20] References

(1) Gaudard P, Mourad M, Eliet J, Zeroual N, Culas G, Rouviere P, et al. Management and outcome of patients supported with Impella 5.0 for refractory cardiogenic shock. Crit Care 2015 Oct 9;19:363-015-1073-8.

(2) Martin C, Papazian L, Perrin G, Saux P, Gouin F. Norepinephrine or dopamine for the treatment of hyperdynamic septic shock? Chest 1993 Jun;103(6):1826-1831.

(3) Thorlund K, Imberger G, Walsh M, Chu R, Gluud C, Wetterslev J, et al. The number of patients and events required to limit the risk of overestimation of intervention effects in meta-analysis--a simulation study. PLoS One 2011;6(10):e25491.

(4) Rajaram SS, Desai NK, Kalra A, Gajera M, Cavanaugh SK, Brampton W, et al. Pulmonary artery catheters for adult patients in intensive care. Cochrane Database Syst Rev 2013 Feb 28;(2):CD003408. doi(2):CD003408.

(5) Yavuz S, Ayabakan N, Goncu MT, Ozdemir IA. Effect of combined dopamine and diltiazem on renal function after cardiac surgery. Med Sci Monit 2002 May;8(5):PI45-50.

(6) Patel GP, Grahe JS, Sperry M, Singla S, Elpern E, Lateef O, et al. Efficacy and safety of dopamine versus norepinephrine in the management of septic shock. Shock 2010 Apr;33(4):375-380.

(7) Copenhagen Trial Unit. TSA—Trial Sequential Analysis. 2011; Available at: http://ctu.dk/tsa/.

(8) Johansson SG, Bieber T, Dahl R, Friedmann PS, Lanier BQ, Lockey RF, et al. Revised nomenclature for allergy for global use: Report of the Nomenclature Review Committee of the World Allergy Organization, October 2003. J Allergy Clin Immunol 2004 May;113(5):832-836.

(9) Dellinger RP, Levy MM, Rhodes A, Annane D, Gerlach H, Opal SM, et al. Surviving Sepsis Campaign: international guidelines for management of severe sepsis and septic shock, 2012. Intensive Care Med 2013 Feb;39(2):165-228.

(10) Koster G, Wetterslev J, Gluud C, Zijlstra JG, Scheeren TW, van der Horst IC, et al. Effects of levosimendan for low cardiac output syndrome in critically ill patients: systematic review with meta-analysis and trial sequential analysis. Intensive Care Med 2015 Feb;41(2):203-221.

(11) Salminen PR, Dahle GO, Moen CA, Wergeland A, Jonassen AK, Haaverstad R, et al. Reperfusion therapy with low-dose insulin or insulin-like growth factor 2; myocardial function and infarct size in a porcine model of ischaemia and reperfusion. Basic Clin Pharmacol Toxicol 2014 Nov;115(5):438-447.

(12) van der Horst IC, Timmer JR, Ottervanger JP, Bilo HJ, Miedema K, Gans RO, et al. Glucose and potassium derangements by glucose-insulin-potassium infusion in acute myocardial infarction. Neth Heart J 2006 Mar;14(3):89-94.

(13) Brok J, Thorlund K, Wetterslev J, Gluud C. Apparently conclusive meta-analyses may be inconclusive--Trial sequential analysis adjustment of random error risk due to repetitive testing of accumulating data in apparently conclusive neonatal meta-analyses. Int J Epidemiol 2009 Feb;38(1):287-298.

(14) Thorlund K, Engstrom J, Wetterslev J, Brok J, Imberger G, Gluud C. User manual for Trial Sequential Analysis (TSA). 2011; Available at: http://ctu.dk/tsa/files/tsa_manual.pdf.

(15) International Conference on Harmonisation Expert Working Group. International conference on harmonisation of technical requirements for registration of pharmaceuticals for human use, ed. ICH harmonised tripartite guideline; guideline for good clinical practice. Available at: http://www.ich.org/fileadmin/Public_Web_Site/ICH_Products/Guidelines/Efficacy/E6/E6_R1_Guideline.pdf.

(16) Cove ME, MacLaren G. Clinical review: mechanical circulatory support for cardiogenic shock complicating acute myocardial infarction. Crit Care 2010;14(5):235.

(17) Aissaoui N, Puymirat E, Simon T, Bonnefoy-Cudraz E, Angoulvant D, Schiele F, et al. Long-term outcome in early survivors of cardiogenic shock at the acute stage of myocardial infarction: a landmark analysis from the French registry of Acute ST-elevation and non-ST-elevation Myocardial Infarction (FAST-MI) Registry. Crit Care 2014 Sep 19;18(5):516-014-0516-y.

(18) Sakr Y, Reinhart K, Vincent JL, Sprung CL, Moreno R, Ranieri VM, et al. Does dopamine administration in shock influence outcome? Results of the Sepsis Occurrence in Acutely Ill Patients (SOAP) Study. Crit Care Med 2006 Mar;34(3):589-597.

(19) Kosiborod M, Inzucchi SE, Krumholz HM, Masoudi FA, Goyal A, Xiao L, et al. Glucose normalization and outcomes in patients with acute myocardial infarction. Arch Intern Med 2009 Mar 9;169(5):438-446.

(20) Poss J, Kriechbaum S, Ewen S, Graf J, Hager I, Hennersdorf M, et al. First-in-man analysis of the i-cor assist device in patients with cardiogenic shock. Eur Heart J Acute Cardiovasc Care 2015 Oct;4(5):475-481.

(21) Oba Y, Lone NA. Mortality benefit of vasopressor and inotropic agents in septic shock: a Bayesian network meta-analysis of randomized controlled trials. J Crit Care 2014 Oct;29(5):706-710.

(22) Povoa PR, Carneiro AH, Ribeiro OS, Pereira AC, Portuguese Community-Acquired Sepsis Study Group. Influence of vasopressor agent in septic shock mortality. Results from the Portuguese Community-Acquired Sepsis Study (SACiUCI study). Crit Care Med 2009 Feb;37(2):410-416.

(23) von Scheidt W, Pauschinger M, Ertl G. Long-term intravenous inotropes in low-output terminal heart failure? Clin Res Cardiol 2016 Jun;105(6):471-481.

(24) Diaz A, Humeres C, Gonzalez V, Gomez MT, Montt N, Sanchez G, et al. Insulin/NFkappaB protects against ischemia-induced necrotic cardiomyocyte death. Biochem Biophys Res Commun 2015 Nov 13;467(2):451-457.

(25) Lichtenstein DA, Meziere GA. Relevance of lung ultrasound in the diagnosis of acute respiratory failure: the BLUE protocol. Chest 2008 Jul;134(1):117-125.

(26) Geerts BF, Aarts LP, Groeneveld AB, Jansen JR. Predicting cardiac output responses to passive leg raising by a PEEP-induced increase in central venous pressure, in cardiac surgery patients. Br J Anaesth 2011 Aug;107(2):150-156.

(27) Rudski LG, Lai WW, Afilalo J, Hua L, Handschumacher MD, Chandrasekaran K, et al. Guidelines for the echocardiographic assessment of the right heart in adults: a report from the American Society of Echocardiography endorsed by the European Association of Echocardiography, a registered branch of the European Society of Cardiology, and the Canadian Society of Echocardiography. J Am Soc Echocardiogr 2010 Jul;23(7):685-713; quiz 786-8.

(28) Monnet X, Anguel N, Naudin B, Jabot J, Richard C, Teboul JL. Arterial pressure-based cardiac output in septic patients: different accuracy of pulse contour and uncalibrated pressure waveform devices. Crit Care 2010;14(3):R109.

(29) Tejedor A, Rivas E, Rios J, Arismendi E, Martinez-Palli G, Delgado S, et al. Accuracy of Vigileo/Flotrac monitoring system in morbidly obese patients. J Crit Care 2015 Jun;30(3):562-566.

(30) Valley TS, Sjoding MW, Goldberger ZD, Cooke CR. ICU Use and Quality of Care for Patients With Myocardial Infarction and Heart Failure. Chest 2016 Sep;150(3):524-532.

(31) Champion HR, Sacco WJ, Hannan DS, Lepper RL, Atzinger ES, Copes WS, et al. Assessment of injury severity: the triage index. Crit Care Med 1980 Apr;8(4):201-208.

(32) Bohadana A, Izbicki G, Kraman SS. Fundamentals of lung auscultation. N Engl J Med 2014 Feb 20;370(8):744-751.

(33) Knaus WA, Draper EA, Wagner DP, Zimmerman JE. APACHE II: a severity of disease classification system. Crit Care Med 1985 Oct;13(10):818-829.

(34) Le Gall JR, Lemeshow S, Saulnier F. A new Simplified Acute Physiology Score (SAPS II) based on a European/North American multicenter study. JAMA 1993 Dec 22-29;270(24):2957-2963.

(35) Zimmerman JE, Kramer AA, McNair DS, Malila FM. Acute Physiology and Chronic Health Evaluation (APACHE) IV: hospital mortality assessment for today's critically ill patients. Crit Care Med 2006 May;34(5):1297-1310.

(36) ARDS Definition Task Force, Ranieri VM, Rubenfeld GD, Thompson BT, Ferguson ND, Caldwell E, et al. Acute respiratory distress syndrome: the Berlin Definition. JAMA 2012 Jun 20;307(23):2526-2533.
